# Novel High Affinity Sigma-1 Receptor Ligands from Minimal Ensemble Docking-Based Virtual Screening

**DOI:** 10.3390/ijms22158112

**Published:** 2021-07-29

**Authors:** Szabolcs Dvorácskó, László Lázár, Ferenc Fülöp, Márta Palkó, Zita Zalán, Botond Penke, Lívia Fülöp, Csaba Tömböly, Ferenc Bogár

**Affiliations:** 1 Biological Research Centre, Institute of Biochemistry, Eötvös Loránd Research Network (ELKH), H-6726 Szeged, Hungary; dvoracsko.szabolcs@brc.hu (S.D.); tomboly.csaba@brc.hu (C.T.); 2 Institute of Pharmaceutical Chemistry, University of Szeged, H-6720 Szeged, Hungary; lazar.laszlo@szte.hu (L.L.); fulop@pharm.u-szeged.hu (F.F.); palko.marta@szte.hu (M.P.); lazarne.zalan.zita@szte.hu (Z.Z.); 3 Department of Medical Chemistry, University of Szeged, H-6720 Szeged, Hungary; penke.botond@med.u-szeged.hu; 4 MTA-SZTE Biomimetic Systems Research Group, Eötvös Loránd Research Network (ELKH), H-6720 Szeged, Hungary

**Keywords:** novel sigma-1 receptor ligands, virtual screening protocol, ensemble docking, radioligand binding assay

## Abstract

Sigma-1 receptor (S1R) is an intracellular, multi-functional, ligand operated protein that also acts as a chaperone. It is considered as a pluripotent drug target in several pathologies. The publication of agonist and antagonist bound receptor structures has paved the way for receptor-based in silico drug design. However, recent studies on this subject payed no attention to the structural differences of agonist and antagonist binding. In this work, we have developed a new ensemble docking-based virtual screening protocol utilizing both agonist and antagonist bound S1R structures. This protocol was used to screen our in-house compound library. The S1R binding affinities of the 40 highest ranked compounds were measured in competitive radioligand binding assays and the sigma-2 receptor (S2R) affinities of the best S1R binders were also determined. This way three novel high affinity S1R ligands were identified and one of them exhibited a notable S1R/S2R selectivity.

## 1. Introduction

Sigma receptor was first identified in 1976 by Martin et al. as an opioid receptor subtype [1]. However it turned out in the early eighties that the pharmacological character of sigma receptor diverges from the other opioid receptors [2,3]. This and the subsequent scientific efforts finally led to the identification of the sigma non-opioid intracellular receptor family [4] with its two members, sigma-1 (S1R) and sigma-2 (S2R) receptors [5]. 

S1Rs are broadly spread in the whole organism (central nervous system (CNS), heart, liver, kidney, lung, muscles [6]). It localizes mainly in the mitochondria-associated endoplasmic reticulum (ER) membrane (MAM). The receptor can also be dynamically translocated inside the cells. It is an intracellular, multi-functional, ligand operated protein that also acts as a chaperone [6].

S1R has a unique amino acid sequence with no mammalian homologues, the canonical isoform contains 223 amino acid residues [7]. Its ligand bound form showed a homotrimeric structure with only one transmembrane helix for each monomeric subunit [8].

The other member of sigma receptor family, S2R is primarily implicated in cancer. It is used as a biomarker for proliferation and its agonists are potent anticancer agents [9]. Sequence of S2R has only been identified recently [10], but its structure is not known yet. In spite of their sequential and structural dissimilarities, S1 and S2 receptors often have common ligands with similar receptor affinities. This can be demonstrated using the S2RSLDB (Sigma 2 Receptor Selective Ligand Database [11]). More than half (392) of the 651 S2R selective ligands in the database have a binding affinity that is only less than 10 times larger to S2R than to S1R. Therefore, finding highly selective ligands of either sigma receptor has remained a challenging task [12,13].

Although S1R might participate in a variety of specific physiological functions, its genuine role has been yet poorly understood. Interestingly, the S1R knock-out mice are viable and fertile and do not show any apparent phenotype changes compared to wild-type mice [14]. The exact physiological role of S1R is difficult to determine as many S1R ligands also bind to other receptor targets (e.g., kappa-receptor, NMDA receptor). S1R interacts with a large range of client proteins [15,16], e.g., with several voltage- and ligand-gated ion channels, modulating their activity. The main functions of S1R are probably modulation of Ca^2+^ release, modulation of cardiac myocyte contractility and inhibition of voltage gated K^+^ channels [17]. The receptor apparently co-localizes with the IP3 receptors on the ER membrane and is responsible for the calcium exchange between ER and mitochondria [18]. S1R was found together with voltage gated K^+^ channels in the membranes and thus it was considered to be an auxiliary subunit of the protein complex [19]. S1R antagonists show GTP-sensitive high affinity binding indicating an existing link between S1R and G-proteins [20]. S1 receptors are considered to be linked to a wide variety of signal transduction pathways. S1R may be involved in the prevention of ER-stress [21]. S1R possesses profound effect on neuronal excitatibility and neurotransmission [22].

The molecular functions of S1R and the role in cellular stress signaling have been recently reviewed in details [16,23]. The multiplicity of intracellular partners and signalization pathways may explain the multiplicity of actions of S1R in different types of cells as well as the involvement of the receptor in a series of physiopathological processes [21]. S1R has been considered as a ‘pluripotent modulator of the living system’ [24], an intracellular signal transduction amplifier [15].

The biochemical pharmacology and pharmacological profile have been reviewed recently in detail [16,23,25,26]. Although S1Rs are spread both in the CNS and the periphery, CNS is the primary site of S1R activity and effects [6]. S1Rs are expressed abundantly in neurons, oligodendrocytes, and ependymocytes. S1R accumulates to nuclear inclusions in neurons in neurodegenerative diseases [27]. S1R is the most important factor for fine-tuning of the cellular calcium homeostasis and thus it is the most promising therapeutic target [21]. Experimental results demonstrated that neuronal S1Rs show protective roles in neurodegeneration and are involved in neurorestauration [28,29,30,31]. S1R activity is necessary for physiological brain plasticity [32]. S1R ligands (e.g., dimethyl tryptamine, DMT) can induce psychotomimetic effects and antidepressant activity. Drug screening experiments demonstrated that S1R may be a target for the therapy of VWM leukodystrophy [33]. S1R activation can protect neurons against ER-stress mediated apoptosis with cerebral ischemia/reperfusion injury [34]. Upregulation of S1Rs ameliorates cell death signaling and execute protective function in ER-stress [35]. As neuroinflammation is a key abnormality in the progression of Alzheimer’s disease (AD), the anti-inflammatory activity of S1R might control the disease-related inflammatory process. Activation of S1R promotes the repair of microglia and restores their physiological functions [36,37].

Cardiac S1Rs regulate response to ER stress and modulate Ca signaling in cardiomyocytes and thus are potential novel targets for special treatment of cardiovascular diseases [38].

S1R ligands are historically classified as agonists, antagonists, and allosteric modulators, although this classification differs from the canonical pharmacological nomenclature. The receptor has a unique property: it does not possess identified intrinsic activity, thus the concept of S1R ‘agonism’ and ‘antagonism’ has no consensual definition [39]. Therefore, the pharmacological character of S1R ligands is determined not only by their inherent properties, but also by the choice and setup of the in vitro or in vivo experiment applied. According to the conventionally accepted classification, ligands inducing characteristic pharmacological responses typical of known agonists or S1R overexpression are characterized as agonists. The identification of the characteristic responses goes back to SKF-10047, the first identified S1R ligand of this kind [40]. S1R antagonists are ligands without inherent neurophysiological effects, but inhibit the effects induced by agonists, or ligands causing similar consequences that appear in S1R knockout animals. Agreeing with Oyer et al. [39], instead of agonist/antagonist classification the term positive and negative ‘modulator’ might be more accurate for defining substances with affinity to S1R.

S1R ligands regulate the self-association of the receptor molecules: agonists preferring the lower level of association; antagonists shift the equilibrium towards larger aggregates [16,26]. Agonists act generally neuroprotective, pro-survival and anti-apoptotic via different signaling pathways [15].

The amino acid sequence of S1R was identified in 1996 [7]. It showed no significant homology with any known mammalian proteins. Therefore, besides the traditional methods, dominantly the ligand based computational design tools could be used for the identification of novel S1R ligands. Design of small molecular S1R ligands was performed first by using the Glennon-model for the receptor binding site and pharmacophore identification [41,42]. Several other pharmacophore models were built later that are reviewed in [43].

**Figure 1:**
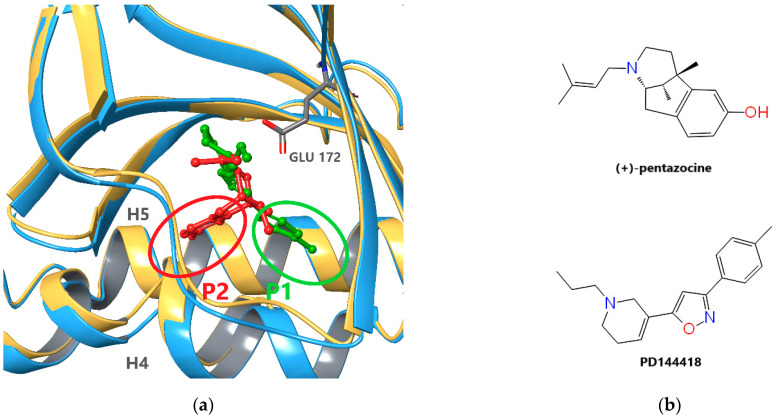
Ligand binding of S1R: (**a**) Superposition of agonist- ((+)-pentazocine, red) and antagonist-(PD144418, green) bound S1R monomers (PDB IDs: 6DK1 chain C (blue) and 5HK1 chain A (gold)). Backbone atoms of residues 170–176 and 197–217 were used if fitting. PD144418 and (+)-pentazocine occupy two different subpockets, P1 and P2, between the C-terminal helixes H4 and H5. (**b**) Chemical structure of ligands (+)-pentazocine and PD144418.

The first X-ray structures of S1R (Protein Data Bank (PDB) [44] IDs: 5HK1 (Figure 1a), 5HK2) were published by Schmidt el al. in 2016 [4]. The experiments uncovered a trimeric structure of the ligand-bound receptor, each monomeric unit being anchored with a single helix in the membrane. The ligands in these complexes were PD144418 (Figure 1b), a high-affinity selective S1R antagonist and 4IBP with a not well-defined pharmacological character. The binding pocket of the receptor is situated in a beta barrel-like unit which shows high structural similarity to oligomeric enzymes from the cupin family [4].

The general scheme of ligand binding in accordance with the Glennon-model involves a positively charged nitrogen, which forms an electrostatic interaction with residue E172. This charged site sits between two dominantly hydrophobic regions: a longer one that occupies the region of the β-barrel that is proximal to the membrane and a shorter one that occupies space near the other end of the β-barrel.

In 2018, Schmidt et al. published the X-ray structures of an agonist ((+)-pentazocine, PDB: 6DK1, see Figure 1) and two antagonists (haloperidol and NE-100, PDB: 6DK0 and 6DJZ, respectively) bound to S1R [45]. Interestingly, the trimeric structure was preserved even in the case of agonist binding. These structures showed high similarity to the previous ones but also revealed slight differences between agonist and antagonist binding. The longer hydrophobic regions of the antagonists (PD144418, haloperidol, and NE-100) and agonist ((+)-pentazocine) occupy two different subpockets, P1 and P2, between the C-terminal helixes H4 and H5 siting on the ER membrane surface (see Figure 1a). The different binding poses also induce structural differences primarily in these helices. Unfortunately, only a single agonist bound X-ray structure ((+)-pentazocine) is available to date. Schmidt et al. [45] presumed that structurally similar agonists, such as (+)-SKF-10,047 adopt a similar binding pose to (+)-pentazocine. In addition, based on their docking studies they suggested that the agonist PRE-084 also adopt a similar pose occupying subpocket P2. However, there is no experimental evidence that connects generally the occupation either of the subpockets and the pharmacological character of the ligand. The binding mode is not known for such structurally divergent S1R agonists like fluvoxamine, cutamesine, or donepezil. The existence of ligands occupying both P1 and P2 subpockets also cannot be excluded.

These structural data made possible the rational design of novel S1R ligands. Pasqual et al. developed a new pharmacophore model based on the PDB structure 5HK1 [22]. Recently, a new docking-based protocol was proposed to predict the affinity of small compounds against S1R [46], which used the 5HK2 structure. Greenfield et al. published an 5HK1-based virtual screening (VS) protocol for the development of novel positive modulators of S1R with neuroprotective effects [47]. These studies were based on X-ray structures with ligands of antagonist-like binding and therefore the ligands that resulted from their VS probably also prefer the same binding mode.

Our aim was to develop a new VS protocol without this bias; using both agonist- (PDB: 6DK1) and antagonist-bound (PDB: 5HK1) X-ray structures in an ensemble docking-based VS protocol. Ensemble docking procedure was introduced by Carlson et al. [48] in 1999 and intended to include the dynamic fluctuations of a protein in computer-aided drug design (for a recent review, see [49]). In this method structures usually collected from molecular dynamics simulations form an ‘ensemble’ of receptor conformations that is used in ligand docking and can improve the performance of virtual screening (see e.g., [50]). Here we use a different method, which selects an optimized minimal ensemble (with only two receptor structures), one from the agonist and another from the antagonist bound X-ray structures. This approach is closely related to the “slow heuristic” knowledge-based ensemble optimization procedure proposed by Swift et al. [51]. For the validation of this protocol, we used high-affinity ligands collected from the BindingDB [52] and a corresponding decoy set generated from the DUD-E [53] database. We have screened our in-house compound library of ~4000 substances with this new method. The S1R binding affinity of the top-ranked ~1% of the racemic compounds was measured with competitive radioligand binding assay. Finally, the binding affinity and the S1R/S2R selectivity of the enantiomers of the best-ranked racemic compounds were also quantified. We have identified three high-affinity compounds that are putative neuroprotective drug candidates for treating AD and other neurodegenerative diseases.

## 2. Results and Discussion

### 2.1. Receptor Model Selection and Validation

From the available ligand-bound S1R structures, we selected two, 5HK1 and 6DK1 [8,45]. In the former complex, the antagonist PD144418 and in the latter the agonist (+)-pentazocine are bound to S1R. The selection of 5HK1 is based on two recent virtual screening studies mentioned earlier [46,47] where this structure performed satisfactorily. The selection of 6DK1 has no alternatives, as this is the only structure where the hydrophobic pocket, P2 is occupied. Both structures are trimeric, therefore we evaluated the performance of each monomers applying the Glide XP docking protocol of the Schrödinger program suite [54]. For this purpose, we used an active set of twenty diverse compounds selected from 190 S1R ligands with subnanomolar affinity collected from the BINDINGDB database [52]. The corresponding decoy set of 1000 singly charged compounds were generated at the on-line surface of the DUD-E database [53].

Unified active and decoy sets were docked to each of the six S1R monomers, and the enrichment of the actives were characterized with three measures suitable for the evaluation of the early enrichment (RIE, BEDROC, enrichment factor (EF) in the top 1% and 2%). The chain A of the 5HK1 structure (5HK1A) outperforms the other two chains in the trimer with a RIE value of 6.08 and a BEDROC(α = 160.9) of 0.315 (see Table 1). In the case of 6DK1, the best values belong to chain C (6DK1C) being 5.77 and 0.318, respectively. The enrichment factor calculated for the top 1% (EF_1%_) shows the same tendency for 6DK1C but cannot distinguish the best performer for the three chains of 5HK1A. Following the slow heuristic method of Swift et al. [51] we selected the chain 6DK1C as the first member of the ensemble and calculated the measures for the three pairs formed with the chains of the 5HK1A structure.

**Figure 2:**
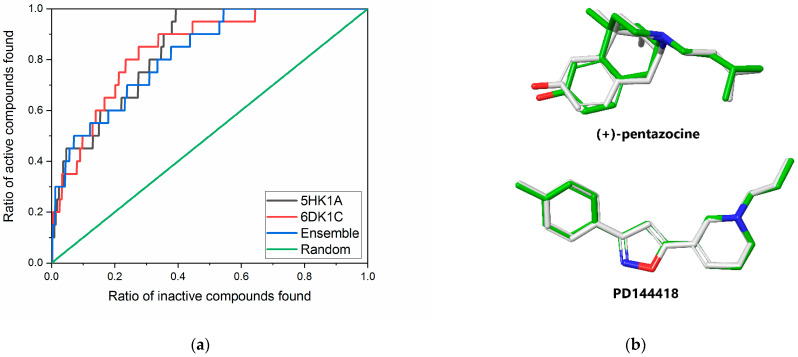
(**a**) Global performance of ensemble docking characterized by the ROC-curve for 5HK1A (black), 6DK1C (red) and ensemble (blue) models compared to random selection (green). (**b**) Comparison of top ranked poses of (+)-pentazocine and PD144418 of ensemble docking (green) to their X-ray structure (gray).

The pair 6DK1C-5HK1A showed the best early enrichment (BEDROC(α = 160.9) = 0.328) although its EF_1%_ is somewhat worse than that obtained for the 6DK1C alone (see Table 1). These structures were added to our minimal ensemble in order to simultaneously increase the early enrichment of our VS and extend the chemical space of the compounds resulted from it. The ensemble ranking provided better early enhancement measures, but the global performance of ensemble docking became slightly weaker than the contributing models alone, as it is shown by the ROC curves in Figure 2a and ROC values in Table 1. 

The calculated measures could be successfully used to select the best receptor combinations but they provide no information on the target preference of the compounds of the test set. To demonstrate the importance of involving both receptor models instead of either of them, we identified the target for each of the best poses obtained from ensemble docking. For 9 of the 20 active and 594 of 1000 decoy compounds were hosted by the 6DK1C and the remaining ones by the 5HK1A S1R structure. These numbers indicate a well-balanced sharing between the two target-structures involved in our minimal ensemble docking setup. In addition, two of the active (A9 and A14) and more than 100 of the decoy compounds from those having their highest ranked pose in 6DK1C entered the hydrophobic pocket, P2. However, none of the actives and only around 30 of the decoys occupied partially the pocket P2 from the compounds preferring 5HK1. This shows that agonist-like binding poses can be accommodated almost equally in both receptor models; however, poses similar to that of (+)-pentazocine appear more frequently in the case of 6DK1C.

After validating our minimal ensemble screening protocol, we also measured the accuracy with which it reproduces the X-ray pose of the original ligands. Both (+)-pentazocine as well as PD144418 were docked with our method. The top ranked poses of both ligands were hosted by their corresponding X-ray protein structure. The RMSD values of the heavy atoms of these ligands in their X-ray and docked poses (Figure 2b) are 0.151 Å for PD144418 and 0.448 Å for (+)-pentazocine.

### 2.2. Setup and Validation of In Vitro Competitive Binding Assays

To measure the binding affinities of selected ligands to S1R and S2R we performed competition binding experiments in guinea pig and rat liver membrane homogenates. Guinea pig liver tissue preparation abundantly contains sigma-1 receptor as compared to other tissues; therefore, it is an appropriate model to investigate the sigma-1 receptor binding properties of synthetic compounds [55,56,57,58,59,60]. The expression level of sigma-2 receptors is higher in rat liver than in guinea pig liver or rat brain [61], therefore, rat liver membrane homogenate was established as the tissue model in sigma-2 receptor binding assays [5,60,61,62,63].

The selective S1R agonist [^3^H]-(+)-pentazocine possessed a saturable, high affinity binding to a single class of sites with an equilibrium dissociation rate constant (K_d_) of 1.8 nM and the maximal density of binding sites (B_max_) of 1072 fmol/mg protein in guinea pig liver membrane preparations at 37 °C. We also determined the K_d_ of the non-selective sigma receptor ligand, [^3^H]-DTG in rat liver membranes in the presence of (+)-pentazocine (100 nM) to mask S1R sites. The K_d_ value was found to be 47 nM (see Figure S1 in Supplementary Information).

**Figure 3:**
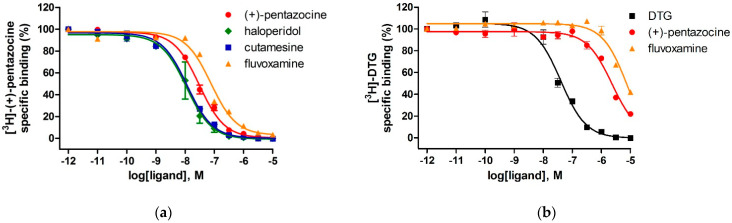
Displacement curves for reference compounds against (**a**) [^3^H]-(+)-pentazocine binding to S1R in guinea pig liver and (**b**) [^3^H]-DTG binding to S2R sites in rat liver membranes. Data are mean percentage of specific binding ± SEM from minimum three independent experiments, each performed in duplicate.

The binding affinities of the compounds for S1R and S2R were determined using in vitro competitive binding assays. The assay conditions were validated with the following S1R and S2R ligands: (+)-pentazocine, fluvoxamine, haloperidol, cutamesine, and DTG. The displacement curves and the measured binding parameters are presented in Figure 3 and Table 2, respectively. Competition binding assays in guinea pig liver membrane homogenate against the S1R specific radioligand [^3^H](+)-pentazocine revealed that all four compounds exhibited nanomolar S1R affinities and induced a similar maximal displacement (100%). The order of potencies of the prototypic sigma ligands were as (+)-pentazocine > haloperidol > cutamesine > fluvoxamine that is consistent with previous S1R pharmacology findings [40,64,65,66,67]. In homologous displacement experiments for the S2R, the non-selective S1R and S2R ligand, DTG exhibited a K_i_ value of 29 nM in rat liver membranes. In order to mask the S1R binding sites of the rat liver membrane preparation 100 nM (+)-pentazocine was applied [5,60].

The S1R ligands, (+)-pentazocine and fluvoxamine were able to compete with [^3^H]DTG with apparently high inhibitory constants in the micromolar range; however, they only partially (c.a. 70–80%) displaced [^3^H]DTG. Furthermore, in our experimental model, (+)-pentazocine and fluvoxamine displayed high overall selectivities as S1R ligands, which is in a good agreement with the results of Narita et al. [65] and Lever et al. [66].

### 2.3. Screening of Our in-House Library

According to our results the minimal ensemble docking procedure described above provided high early enrichment in our test set. This validates our method for virtual screening of large compound libraries, as well. Our in-house compound library contains ~4000 dominantly drug-like molecules. The compound library contains mainly various derivatives of 1,2- and 1,3-bifunctional compounds (derivatives of amino acids, amino alcohols, hydroxy acids, diamines etc. with acyclic, aromatic, alicyclic and heterocyclic scaffolds). Based on the screening, the highest ranked 40 molecules (top 1%) were selected for experimental testing. These molecules had their docking scores between −9.38 kcal/mol and −13.76 kcal/mol.

As a first step of the experimental evaluation, competition binding experiments were carried out by incubating guinea pig liver membranes with 2.4 nM of [^3^H](+)-pentazocine in the presence of increasing concentrations (between 10^−11^ M and −10^−5^ M) of the selected unlabeled ligands.

Table 3 lists twelve compounds that showed activity in this range of concentration. Corresponding displacement curves can be found in the Supplementary Materials (Supplemental Figure S2). The best compounds L1, L2, and L3 have inhibitory constants of 32 nM, 91 nM, and 110 nM, respectively.

**Table 3:** Sigma-1 receptor binding parameters for racemic ligands in guinea pig liver membrane homogenates and the corresponding docking scores.

ID	Measured K_i_ (nM)	Docking Score (kcal/mol)	Chemical Name	Structure of Best Docked Stereoisomer
L1 ^1^	32.0	−11.62	*N*-Benzyl-6,7-dimethoxy-1,2,3,4-tetrahydro-1-isoquinolineethanamine dihydrochloride	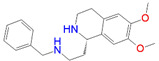
L2 ^1^	91.0	−12.41	3-Amino-*N*-(2-fluoro-3-(trifluoromethyl)benzyl]-3-phenylpropanamide hydrochloride	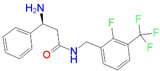
L3 ^2^	110.0	−11.95	1-[(4-Methoxyphenoxy)methyl]-2-(1,2,3,4-tetrahydroisoquinolin-2-yl)ethanol	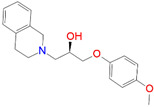
L4 ^1^	420.0	−12.47	3-Amino-*N*-(3-fluoro-5-(trifluoromethyl)benzyl]-3-phenylpropanamide hydrochloride	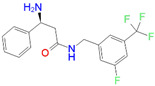
L5 ^1^	463.0	−10.74	(±)-*diendo*-3′-amino-1-benzyl-5′,8′-methano-4′a,5′,8′,8′a-tetrahydrospiro[piperidine-4,2′(1′*H*)-quinazolin]-4′(3′*H*)-one	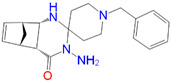
L6 ^2^	1036.0	−11.12	*N*-Benzyloxycarbonyl-(9-methyl-2,3,4,9-tetrahydro-1*H*-pyrido[3,4-*b*]indol-1-yl)methanamine	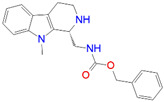
L7 ^2^	1381.0	−11.56	(1*S*,3*R*,4*R*,6*R*)-3-(benzylamino)methyl)-7,7-dimethylbicyclo[4.1.0]heptane-3,4-diol	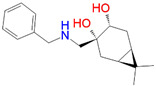
L8 ^2^	1534.0	−10.68	3-Amino-*N*-(2-fluoro-3-(trifluoromethyl)benzyl]-2-methylpropanamide trifluoroacetate	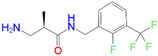
L9 ^1^	1716.0	−10.87	*N*-Benzyloxycarbonyl-(2,3,4,9-tetrahydro-1*H*-pyrido[3,4-*b*]indol-1-yl)methanamine	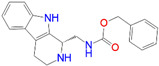
L10 ^1^	2154.0	−11.58	(4R*,11bR*)-9,10-Diethoxy-4-[4-(dimethylamino)phenyl]-1,3,4,6,7,11b-hexahydro-2*H*-pyrimido[6,1-*a*]isoquinoline	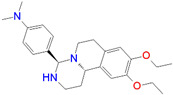
L11 ^1^	2381.0	−10.55	1-{[(Benzyloxycarbonyl)amino]methyl}-6,7-dimethoxy-1,2,3,4-tetrahydroisoquinoline	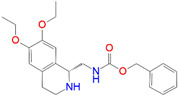
L12 ^2^	3266.0	−9.69	(1R*,9bR*)-1-{[(4-Chlorophenyl)thio]methyl}-7,8-diethoxy-1,4,5,9b-tetrahydro-2*H*-azeto[2,1*a*]-isoquinoline	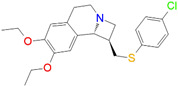

^1^ Preferred target: 6DK1C. ^2^ Preferred target: 5HK1A.

These ligands have common structural elements: a basic amine site (present in all high affinity S1R ligands), that is necessary for the formation of an electrostatic bond with Glu172 of the receptor. It is typically flanked by two different hydrophobic groups. This common structure matches to the Glennon pharmacophore model (amine site together with a primary and a secondary hydrophobic region which is necessary for the proper fitting into the binding site of S1R [42]). The hydrophobic parts are mostly aromatic and/or rigid heterocyclic sites.

Five of the 40 selected compounds possessed nanomolar activities, the remaining ones bound weaker. We also investigated the preferred receptor models for these compounds (see footnotes in Table 3). In case of five molecules, the top ranked pose belongs to 5HK1A and in case of seven ones to 6DK1C. Interestingly, only one of the latter set (L7) entered the P2 pocket preferred by the agonist (+)-pentazocine (see supplemental Figure S3). It has a binding constant in the low micromolar range (1310 nM). The experimental binding constants show moderate correlation with docking scores (see supplemental Figure S4) the corresponding coefficient of determination is R^2^ = 0.41.

### 2.4. Binding Affinity of the Enantiomers of the Best Compounds to S1R and S2R

**Figure 4:**
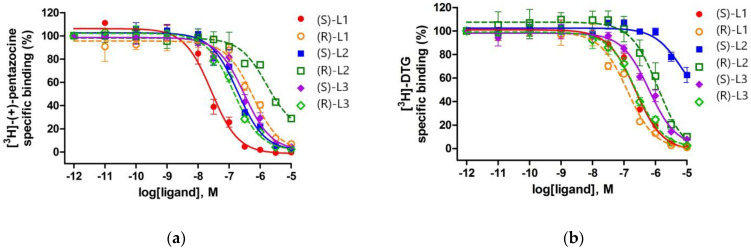
Displacement curves for corresponding ligands against (**a**) [^3^H]-(+)-pentazocine binding to S1R sites in guinea pig liver membranes and (**b**) [^3^H]-DTG binding to S2R sites in rat liver brain membranes using (+)-pentazocine (100 nM) to mask S1R sites. Data are mean percentage of specific binding ± SEM from minimum three independent experiments, each performed in duplicate.

The three highest ranked compounds are chiral molecules, which have two enantiomeric forms. The enantiomers were separated and their binding affinities to S1R and S2R were measured separately (Table 4 and Figure 4). The binding constants to S1R were determined similarly to those of the racemic compounds. In the case of S2R, competition binding experiments were carried out by incubating rat liver membranes with 17 nM of [^3^H]DTG (K_d_ = 47 nM) in the presence of 100 nM (+)-pentazocine to mask S1R binding sites.

(*S*)-L1 (K_i_ = 11 nM) showed much higher affinity for S1R than S2R sites and exhibited a similar, 15-fold S1R selectivity, as was previously shown by the fluoroethyl analog of S1R agonist SA4503 (cutamesine), where the ratio was K_i_S2R/K_i_S1R = 14.1 [66]. (*R*)-L3 (K_i_ = 58 nM) possessed a good binding affinity towards S1R and displayed a 3-fold lower affinity to S2R. Compound (*S*)-L2 (K_i_ = 81 nM) had a moderate affinity for the S1R site and the most favorable S1R selectivity (K_i_S2R/K_i_S1R = 63). Compounds (*S*)-L3 (K_i_ = 132 nM), (*R*)-L1 (K_i_ = 252 nM), and (*R*)-L2 (K_i_ = 699 nM) showed modest affinities towards S1R, and weak S2R bindings, with the exception of compound (*R*)-L1 which is weakly S2R selective.

### 2.5. S1R Binding Poses of Enantiomers of the New Compounds

**Figure 5:**
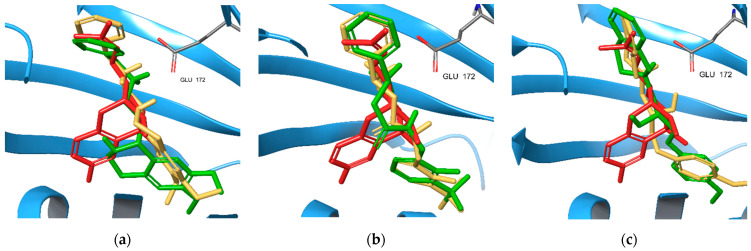
Comparison of the binding poses of the (*S*) (gold) and (*R*) (green) enantiomers of the highest ranked new S1R ligands (**a**) L1, (**b**) L2, and (**c**) L3. The binding pose of (+)-pentazocine (red) is also shown.

The highest ranked binding poses of the enantiomer pairs of the new compounds L1, L2, and L3 were extracted from Glide XP docking results (Figure 5). Each of them resembles the known binding pose of the antagonist. Only (*R*)-L1 enters, at least partially, the binding pocket P2 occupied by (+)-pentazocine in the original X-ray structure. In the case of L1, these poses belong to the complex formed with the 6DK1A S1R model. (*S*)-L1 has a better docking score (−11.62 kcal/mol) than (*R*)-L1 (−10.97 kcal/mol) in accordance with the measured K_i_ values (Table 4). The most preferred binding mode of (*R*)/(*S*)-L2 belongs to chain 5HK1A/6DK1C, respectively, with a docking score of −12.41/−12.28 kcal/mol. Their order is opposite to that obtained from our measurements. Finally, both enantiomers of L3 compound prefer 5HK1A binding and docking score values of −10.88 kcal/mol and −11.95 kcal/mol for the (*S*) and (*R*) enantiomers properly reflect their experimental binding affinities.

## 3. Materials and Methods

### 3.1. Virtual Screening

#### 3.1.1. Receptor Structures

Two receptor structures were selected for our virtual screening studies: the agonist-bound 6DK1 with (+)-pentazocine [45], and the antagonist bound 5HK1 with PD144418 [8]. These structures were downloaded from PDB database [44] and cleaned up using the Protein Preparation Wizard of Schödinger’s package [29].

#### 3.1.2. Model Validation Libraries

In our enrichment study, the compounds of the active set were collected from the BindingDB [52]. We selected 190 high affinity S1R ligands with K_i_ ≤ 1 nM. From this set we extracted a diverse subset of 20 compounds (see Supplemental Figure S5). The pairwise similarity matrix was calculated using the Tanimoto measure from the radial binary fingerprints (extended connectivity fingerprints) with four iterations (ECFP4) within the Canvas program of the Schrödinger Suite [54]. The similarity matrix of the selected compounds is presented in Supplemental Figure S6. A diverse set of 20 compounds was selected using the sphere exclusion method with a sphere radius of 0.5. Fifty molecules were selected to each active compound using the web interface of DUD-E database [53] resulting in a decoy set of 1000 members. A search algorithm of DUD-E selects drug-like compounds from its ZINC-based dataset having similar physicochemical properties (molecular weight, estimated water−octanol partition coefficient, number of rotatable bonds, hydrogen bond acceptors, and hydrogen bond donors and net charge at physiological pH) to the actives. The dissimilarity to actives is guaranteed by a selection method based on the Tanimoto similarity calculated from ECFP4 fingerprints.

#### 3.1.3. Docking Protocol

The compounds of the active and decoy sets were docked to the targets using the Glide-based Virtual Screening Workflow from the Schrödinger package [54]. Prior to docking, the necessary fields were calculated on a uniform, 1Å-spaced grid inside a cube with ~32 Å-long edges. The grid centers were located at the center of the ligands from the X-ray structures used. Compounds were prepared for docking with the Ligprep [54] and used in an extra precision (XP) docking protocol [54] applying OPLS3e force-field [68] for parametrization.

In ensemble docking, the compounds were docked to each receptor conformation and the best score obtained this way was selected as final score for each ligand.

#### 3.1.4. Model Evaluation

To prove the effectivity of the active compound selection we used well-established metrics. Having a unified set (S_AD_) with N compounds made of an active set (S_A_) with A and a decoy set (S_D_) with N-A molecules, the average ratio of the molecules from S_A_ and S_D_ in an arbitrary random sample taken from S_AD_ is A/N. The goal of a VS is to find a proper ordering of these compounds that enriches the actives among the highest ranked compounds. The enrichment factor EF_x%_ gives the ratio of the actives (a_x%_) in the top x% ($n_{x\%}$) of the ordered S_AD_, as a multiple of that calculated in a random sample (A/N), i.e.,

(1) EFx%=ax%nx%AN.

This quantity is unsuitable for comparison of VS studies with different databases and highly dependent on the A/N ratio. However, it is a proper measure for ranking the effectiveness of VS-s with different target models using the same compound set (Truchon and Bayly, 2007) [69]. In our study we used EF values corresponding to the top 1% and 2%.

The overall performance of the VS procedure can be visualized using the receiver operating characteristics (ROC) curve. The ROC curve is drawn by moving across the ranked list of compounds, and for each rank k, the ratio of cumulative count of actives (sensitivity, true positive rate) are plotted as a function of the ratio of cumulative count of inactives (1-specificity, false positive rate). Area under the ROC curve (ROC-AUC or simply ROC) measures the overall performance of VS. In the optimal case (all the actives are highest ranked) its value is 1.0, in random case it is equal to 0.5.

Our goal was to use only the top 1% of the ranked compound library in radioligand binding tests therefore we applied measures that characterize the early enrichment of our screening process. Such a score is the Boltzmann-enhanced Discrimination of ROC (BEDROC) [69]. The relative ranks of actives ($x_{i}=\frac{r_{i}}{N}$; $r_{i}$ being the rank of *i*-th active compound) are weighted using Boltzmann distribution function with an α parameter in the exponent. With a proper choice α it can be used to emphasize the role of the highest-ranked active compounds The larger the α is, the smallest set of the top ranked compounds dominates the score value, e.g., if α = 160.9 (80.5) the top 1% (2%) of the ranked compounds account for the 80% of the BEDROC value. Larger positive BEDROC values indicate better screen performance. BEDROC is a linear function of Robust Initial Enhancement (RIE) [69] transforming its values to the [0,1] interval.

$$\mathrm{RIE}=\frac{N}{A}\sum_{i=1}^{A}e^{-{\alpha x}_{i}}\;\frac{e^{\frac{\alpha }{N}-1}}{1-e^{-\alpha }},$$

BEDROC=RIE1Nsinh(∝2)cosh(∝2)−cosh(∝2−∝AN)+(1−e−αN−AN)−1

### 3.2. Binding Assays

#### 3.2.1. Materials

(+)-Pentazocine, haloperidol and buffer components (Tris-HCl, inhibitors), 1,3-di(2-tolyl)guanidine were purchased from Sigma-Aldrich Kft. (Budapest, Hungary). The radioligand [^3^H]-(+)-pentazocine (s.a., 1.98 TBq/mmol) and [^3^H]-DTG (s.a., 363 Gbq/mmol) were prepared in the Laboratory of Chemical Biology (BRC, Hungary). Tritium labeling was carried out in a self-designed vacuum manifold [70] and radioactivity was measured with a Packard Tri-Carb 2100 TR liquid scintillation analyzer using Insta Gel scintillation cocktail of PerkinElmer. Drugs were dissolved at 1 mM in dimethyl sulfoxide (DMSO) and were stored at −20 °C, and then diluted in the binding buffer.

The sigma receptor ligands in Table 3 were synthesized with the common methods of the preparative organic chemistry; details of the syntheses and the characterization of the compounds are summarized in Supplementary Information.

#### 3.2.2. Preparation of Membrane Homogenates for S1R and S2R Binding Assays

Male Wistar-Harlan rats (n = 10, 225–250 g) and guinea pigs (n = 10, 350–400 g) were handled according to the European Communities Council Directive (2010/63/EU) and to the Regulations on Animal Protection (40/2013. (II. 14.) Korm. r.) of Hungary. The researchers did the best effort to minimize the number of animals and their suffering. Preparation of guinea pig and rat liver homogenates was performed according to a previous method [60] with a slight modification. Euthanized male guinea pig and rats were killed by decapitation, and their livers were removed rapidly. Minced fresh livers were homogenized in ice-cold homogenization buffer (10 mM NaH_2_PO_4_ pH 7.4, 0.32 M sucrose, 1 mM MgSO_4_, 10 μg/mL leupeptin, 1 μg/mL pepstatin A, 5 μg/mL soybean trypsin inhibitor, 0.5 mM EGTA, 1 mM AEBSF) using a Braun Teflon-glass homogenizer at the highest rpm for 30 s. The homogenate was centrifuged at 17,000× *g* for 10 min (4 °C). Supernatant was recentrifuged at 100,000× *g* for 60 min at 4 °C. The resulting pellet was resuspended in 10 volumes homogenization buffer, homogenized by a glass homogenizer and stored in aliquots at −80 °C. The protein content of the samples was measured by the Bradford method [71] and samples were diluted in assay buffer to obtain the appropriate amount for the assay.

#### 3.2.3. Saturation Binding Experiments

The equilibrium dissociation constant (K_d_) and the maximum number of binding sites (B_max_) were determined by saturation binding experiments. It was performed at 37 °C for 90 min in 50 mM Tris–HCl binding buffer (pH 8.0) with increasing concentrations of [^3^H](+)-pentazocine (0.05–235 nM) or [^3^H]DTG (0.2–343 nM) in the absence (total binding) or presence (non-specific binding) of 10 µM haloperidol. [^3^H]DTG is a non-selective sigma receptor ligand, and the addition of unlabeled 100 nM (+)-pentazocine is required to mask the S1R sites. The incubation was terminated by diluting the samples with an ice-cold wash buffer (50 mM of Tris–HCl, pH 8.0), followed by repeated washing and rapid filtration through Whatman GF/B glass fiber filters (Whatman Ltd., Maidstone, UK) presoaked with 0.1% polyethyleneimine. Filtration was performed with a 24-well Brandel Cell Harvester (Gaithersburg, MD, USA). Filters were air-dried and immersed into Ultima Gold MV scintillation cocktail, and then radioactivity was measured with a TRI-CARB 2100TR liquid scintillation analyzer (Packard, Perkin Elmer, Waltham, MA, USA).

#### 3.2.4. Radioligand Binding Assays of Sigma-1 Receptor

Binding assays for the sigma-1 receptor were performed at 37 °C for 90 min in a 50 mM Tris–HCl binding buffer (pH 8.0) in plastic tubes in a total assay volume of 1 mL that contained 0.5 mg/mL of a membrane protein. Competition binding experiments were carried out by incubating guinea pig liver membranes with 2.4 nM of [^3^H](+)-pentazocine (K_d_ = 1.8 nM) in the presence of increasing concentrations (10^−11^–10^−5^ M) of various competing unlabeled ligands. Non-specific binding was determined in the presence of 10 µM of haloperidol. The incubation was terminated by diluting the samples with an ice-cold wash buffer (50 mM of Tris–HCl, pH 8.0), followed by repeated washing and rapid filtration through Whatman GF/B glass fiber filters (Whatman Ltd., Maidstone, UK) presoaked with 0.1% polyethyleneimine. Filtration was performed with a 24-well Brandel Cell Harvester (Gaithersburg, MD, USA). Filters were air-dried and immersed into Ultima Gold MV scintillation cocktail, and then radioactivity was measured with a TRI-CARB 2100TR liquid scintillation analyzer (Packard, Perkin Elmer, Waltham, MA, USA).

#### 3.2.5. Radioligand Binding Assays of Sigma-2 Receptor

Binding assays for the sigma-2 receptor were performed at 37 °C for 120 min in a 50 mM Tris–HCl binding buffer (pH 8.0) in plastic tubes in a total assay volume of 1 mL that contained 0.6 mg/mL of a membrane protein. Competition binding experiments were carried out by incubating rat liver membranes with 17 nM of [^3^H]DTG (K_d_ = 47 nM) in the presence of 100 nM (+)-pentazocine to mask S1R binding sites. Competing ligands were used at 10^−11^–10^−5^ M concentrations. Non-specific binding was determined in the presence of 10 µM of haloperidol. The incubation was terminated by diluting the samples with an ice-cold wash buffer (50 mM of Tris–HCl, pH 8.0), followed by same steps as described sigma-1 binding assay.

#### 3.2.6. Data Analysis

The results of the competition binding studies are reported as means ± S.E.M. of at least three independent experiments each performed in duplicate. In competition binding studies, the inhibitory constants (K_i_) were calculated from the inflection points of the displacement curves using nonlinear least-square curve fitting and the Cheng–Prusoff equation, K_i_ = EC_50_/(1 + [ligand]/K_d_). All data and curves were analyzed by GraphPad Prism 5.0 (San Diego, CA, USA).

## 4. Conclusions

Applying an enrichment-based model selection procedure, we have established a minimal ensemble VS protocol based on the chains with the best enrichment from the antagonist bound-5HK1 and the agonist-bound 6DK1 S1R structures. This ensemble VS protocol uses only two target structures, chain A from 5HK1 and chain C from 6DK1 models. The protocol could properly reproduce the expected binding modes of the original ligands obtained from the X-ray structures. The validity of our new minimal ensemble screening setup is supported by the well-balanced distribution of the compounds of the test set between the two receptor models. In addition, in case of about 10 percent of the members from active and decoy sets, the top ranked poses entered the hydrophobic pocket, P2 that is also preferred by (+)-petazocine according to the X-ray structure (PDB:6DK1 [45]). Our in-house molecular library was screened using this method and the selected molecules were tested in competitive radioligand binding assay. The experimental results proved that this novel protocol is suitable to select the active compounds with a high success rate. This may indicate that screening of larger libraries could provide new ligands either with agonist- or with antagonist-like binding mode. In addition, we found new high-affinity S1R ligands, among which one compound possessed a notable S1R/S2R selectivity as well.

## Figures and Tables

**Table 1:** Calculated measures of the VS efficiency using a protocol based on single chains from structures 5HK1 or 6DK1 and ensemble docking to the chain pairs 6DK1C-5HK1A, 6DK1C-5HK1B, and 6DK1C-5HK1C.

Model	RIE	BEDROC (α = 160.9)	BEDROC (α = 80.5)	EF_1%_	EF_2%_	ROC
6DK1A	5.00	0.290	0.245	15.3	10.2	0.82
6DK1B	4.74	0.261	0.219	10.2	7.6	0.81
**6DK1C**	**5.77**	**0.318**	**0.291**	**20.4**	**10.2**	**0.84**
**5HK1A**	**6.08**	**0.315**	**0.284**	**10.2**	**10.2**	**0.84**
5HK1B	5.92	0.174	0.201	5.1	7.6	0.86
5HK1C	5.30	0.212	0.203	10.2	7.6	0.85
**6DK1C-5HK1A**	**6.37**	**0.328**	**0.316**	**15.3**	**15.3**	**0.82**
6DK1C-5HK1B	5.89	0.179	0.221	10.2	10.2	0.81
6DK1C-5HK1C	6.11	0.208	0.238	5.1	20.2	0.85

**Table 2:** Sigma-1 and sigma-2 receptor binding affinity (K_i_) and S2R/S1R selectivity for known sigma receptor ligands.

Ligand	S1R K_i_ ± S.E.M. (nM)	S2R K_i_ ± S.E.M. (nM)	Selectivity (S2R/S1R)
(+)-pentazocine	4.8 ± 0.4	1698 ± 103	354
DTG	n.d.	29 ± 4	n.d.
fluvoxamine	31 ± 3	6187 ± 296	200
haloperidol	5.2 ± 1.3	n.d.	n.d.
cutamesine	5.5 ± 1.1	n.d.	n.d.

n.d.: not determined.

**Table 4:** Sigma-1 and Sigma-2 receptor binding parameters and S2R/S1R selectivity for a panel of ligands.

Ligand	S1R K_i_ ± S.E.M. (nM)	S2R K_i_ ± S.E.M. (nM)	Selectivity (S2R/S1R)
(*S*)-L1	11 ± 3	169 ± 15	15.4
(*R*)-L1	252 ± 12	94 ± 5.5	0.4
(*S*)-L2	81 ± 3	5108 ± 960	63
(*R*)-L2	699 ± 57	920 ± 25	1.3
(*S*)-L3	132 ± 23	463 ± 33	3.5
(*R*)-L3	58 ± 3	176 ± 21	3

## Data Availability

External data sources used in this study are cited in article. The extracted data is available in Supplementary Material.

## Supplementary Materials

The following are available online at https://www.mdpi.com/article/10.3390/ijms22158112/s1.
