# Supplementary material for: Novel High Affinity Sigma-1 Receptor Ligands from Minimal Ensemble Docking-Based Virtual Screening
Source: Int J Mol Sci. 2021 Jul 29;22(15):8112. doi: 10.3390/ijms22158112 (PMC8347176; doi:10.3390/ijms22158112)

Supplementary Information to:

Novel high affinity sigma-1 receptor ligands found by a minimal ensemble docking-based virtual screening protocol

Sz. Dvorácskó[1], L. Lázár[2], F. Fülöp[2†], M. Palkó[2], Z. Zalán[2], B. Penke[3], L. Fülöp[3*], C. Tömböly[1]. F. Bogár[3,4*]

1 Institute of Biochemistry, Biological Research Centre, Eötvös Loránd Research Network (ELKH), H-6726 Szeged, Temesvári krt. 62, Hungary
2 Institute of Pharmaceutical Chemistry, University of Szeged, H-6720 Szeged, Eötvös u. 6, Hungary
3 Department of Medical Chemistry, University of Szeged, H-6720 Szeged, Dóm Square 8, Hungary
4 MTA-SZTE Biomimetic Systems Research Group, Eötvös Loránd Research Network (ELKH), H-6720 Szeged, Dóm Square 8, Hungary
* Correspondence: FB: bogar@sol.cc.u-szeged.hu; LF: fulop.livia@med.u-szeged.hu

Contents

1. **Synthetic procedures** **3–14**
2. **^{1}H and ^{13}C NMR spectra of new compounds** **15–32**
3. **Chiral HPLC chromatograms of compounds (*R*)-3, (*S*)-3, (*R*)-(9) and (*S*)-9** **34–35**

1. Synthetic procedures

1.1. General methods

Commercially available compounds were used as obtained from suppliers (Merck Ltd., Budapest, Hungary, and VWR International Ltd., Debrecen, Hungary). The (*R*)- and (*S*) enantiomers of *O*-(4-methoxyphenyl)glycidol were purchased from Chemspace Europe Office (Riga, Latvia) and Synnovator, Inc. (Cary, NC, USA), respectively. Chromatographic separations were carried out on Merck Kieselgel 60 (0.040-0.063 mm) and Merck Aluminum oxide 90 (active neutral, 0.063-0.200 mm) (Merck Ltd., Budapest, Hungary). Merck Kieselgel $60F_{254}$ plates were used for TLC.

Melting points were measured on a Hinotek X-4 melting point apparatus (Hinotek, Ningbo, China). Elemental analyses were performed with a Perkin-Elmer 2400 CHNS elemental analyzer (PerkinElmer Inc., Waltham, MA, USA). Merck Kieselgel 60F254 plates were used for TLC.

Optical rotations were determined on a Perkin-Elmer 341 polarimeter (PerkinElmer Inc., Shelton, CT, USA).

The ^{1}H and ^{13}C-NMR spectra were recorded in DMSO-d_6, $CDCl_3$ and D_2O solutions in 5 mm tubes at room temperature, on a Bruker AVANCE DRX 400 and DRX-500 spectrometers (Bruker Biospin, Karlsruhe, Baden Württemberg, Germany) at 500 (^{1}H) and 125 (^{13}C) MHz, with the deuterium signal of the solvent as the lock and TMS as internal standard (^{1}H, ^{13}C). Chemical shifts are expressed in ppm (δ) relative to TMS as the internal reference. *J* values are given by Hz. NOESY spectrum of (±)-**24** was obtained by using the standard Bruker pulse program noesygpphpp (2D phase-sensitive NOESY with gradient pulses in mixing time and purge pulses before relaxation delay d1).

The synthesis of the following ligands are provided in the Supplement:

L1, ***N*-Benzyl-6,7-dimethoxy-1,2,3,4-tetrahydro-1-isoquinolineethanamine dihydrochloride**

L2, **3-Amino-*N*-(2-fluoro-3-(trifluoromethyl)benzyl]-3-phenylpropanamide hydrochloride**

L3, **1-[(4-Methoxyphenoxy)methyl]-2-(1,2,3,4-tetrahydroisoquinolin-2-yl)ethanol**

L4, **3-Amino-*N*-(3-fluoro-5-(trifluoromethyl)benzyl]-3-phenylpropanamide hydrochloride**

L6, ***N*-Benzyloxycarbonyl-(9-methyl-2,3,4,9-tetrahydro-1*H*-pyrido[3,4-*b*]indol-1-yl)methanamine**

L8, **3-Amino-*N*-(2-fluoro-3-(trifluoromethyl)benzyl]-2-methylpropanamide trifluoroacetate**

L9, ***N*-Benzyloxycarbonyl-(2,3,4,9-tetrahydro-1*H*-pyrido[3,4-*b*]indol-1-yl)methanamine ~~[(±)-21]~~**

L10, **(4*R**,11b*R**)-9,10-Diethoxy-4-[4-(dimethylamino)phenyl]-1,3,4,6,7,11b-hexahydro-2*H*-pyrimido-[6,1-*a*]isoquinoline**

L11, **1-{[(Benzyloxycarbonyl)amino]methyl}-6,7-dimethoxy-1,2,3,4-tetrahydroisoquinoline**

L12,**(1*R**,9b*R**)-1-{[(4-Chlorophenyl)thio]methyl}-7,8-diethoxy-1,4,5,9b-tetrahydro-2*H*-azeto[2,1-*a*]isoquinoline**

The syntheses of

L5, **(±)-*diendo*-3′-amino-1-benzyl-5′,8′-methano-4′a,5′,8′,8′a-tetrahydrospiro[piperidine-4,2′(1′*H*)-quinazolin]-4′(3′*H*)-one** and

L7, **(1*S*,3*R*,4*R*,6*R*)-3-(benzylamino)methyl)-7,7-dimethylbicyclo[4.1.0]heptane-3,4-diol**

were described earlier [1], [2].

1.2. Synthesis of racemic ligand-1 (L1, compound 5) and its enantiomers [(*R*)-5 and (*S*)-5]

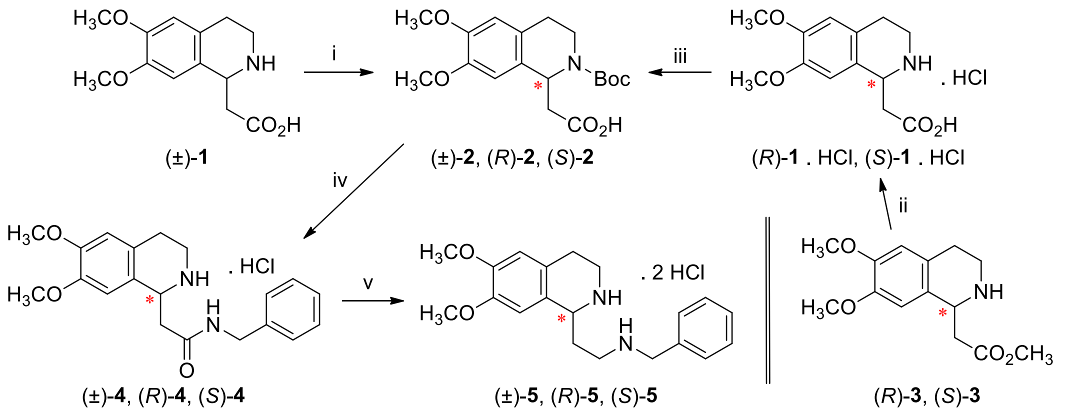

Scheme S1. Synthesis of compounds (±)-**5**, (*R*)-**5,** and (*S*)-**5**.

Reagents and conditions (i) Boc_2O (1.1 equiv), NaOH (1 equiv), H_2O–1,4-dioxane, 0 °C, 3 h, then r.t., 3 h (77%); (ii) HCl, H_2O, 110°C, 12 h (97-98%); (iii) (i) Boc_2O (1.1 equiv), NaOH (2 equiv), H_2O–1,4-dioxane, 0 °C, 3 h,

then r.t., 3 h (74-80%); (iv) 1. $ClCO_2Et$ (1 equiv), Et_3N (1 equiv), $BnNH_2$ (1 equiv), toluene–CH_2Cl_2, –10 °C to reflux, 2. 22% HCl in EtOH, MeOH, Et_2O (57-65%); (v) 1. NaOH, 2. $LiAlH_4$ (3.3 equiv), THF, reflux, 5 h, 3. 22% HCl in EtOH, MeOH, Et_2O (51-54%).

(*R*)-6,7-Dimethoxy-1,2,3,4-tetrahydro-1-isoquinolineacetic acid hydrochloride [compound **(*R*)-1**]
A mixture of methyl (*R*)-6,7-dimethoxy-1,2,3,4-tetrahydro-1-isoquinolineacetate [compound (*R*)-**3**, 12.50 g, 47 mmol)], conc. hydrochloric acid (25 mL), and H_2O (125 mL) was stirred at 110 °C for 12 h. The solution was evaporated, and acetone (100 mL) was added to the solid residue. Crystals were filtered off and washed with acetone (3 × 50 mL). The crude product was used for further transformations without purification. For analytical purposes, a sample was recrystallized from AcOH–acetone.
Yield: 13.20 g (97%), pale beige crystals, m.p.: 215-218 °C. ^1H NMR (500 MHz, D_2O): δ = 2.90-3.16 (4H, m), 3.32-3.40 (1H, m), 3.44-3.55 (1H, m), 3.72 (6H, s), 4.78-4.84 (1H, m), 6.73 (1H, s), 6.77 (1H, s). ^{13}C NMR (125 MHz, D_2O): δ = 24.3, 37.0, 39.5, 51.4, 55.6, 55.8, 108.8, 111.8, 122.5, 124.6, 147.4, 148.1, 173.8. Elemental analysis: calcd. (%) for $C_{13}H_{18}ClNO_4$ (287.74): C, 54.26; H, 6.31; Cl, 12.32; N, 4.87; found: C, 54.15; H, 6.19; Cl, 12.47; N, 4.66.

(*S*)-6,7-Dimethoxy-1,2,3,4-tetrahydro-1-isoquinolineacetic acid hydrochloride [compound **(*S*)-1**]
Compound (*S*)-**1** was prepared from (*S*)-6,7-dimethoxy-1,2,3,4-tetrahydro-1-isoquinolineacetate [compound (*S*)-**3**] according to the above procedure described for compound (*R*)-**1**.
Yield: 13.35 g (98%), pale beige crystals, m.p.: 217-219 °C. The ^1H and ^{13}C NMR spectra of compound (*S*)-**1** were identical to those of (*R*)-**1**. Elemental analysis: calcd. (%) for $C_{13}H_{18}ClNO_4$ (287.74): C, 54.26; H, 6.31; Cl, 12.32; N, 4.87; found: C, 54.38; H, 6.22; Cl, 12.08; N, 4.67.

6,7-Dimethoxy-2-(*tert*-butoxycarbonyl)-1,2,3,4-tetrahydro-1-isoquinolineacetic acid [compound **(±)-2**]
A solution of di-*tert*-butyl dicarbonate (Boc_2O, 9.60 g, 44 mmol) in 1,4-dioxane (40 mL) was added in small portions to a stirred and ice-cooled solution of 6,7-dimethoxy-1,2,3,4-tetrahydro-1-isoquinolineacetic acid [3] [compound (±)-**1**, 10.05 g, 40 mmol] and NaOH (1.60 g, 40 mmol) in H_2O (40 mL). The mixture was stirred under ice-cooling for 3 h and at room temperature for 3 h and allowed to stand at room temperature overnight. Then it was evaporated to 1/3 volume, and $CHCl_3$ (100 mL) was added. The mixture was cooled, and its pH was adjusted to 2.5 by using 10% H_2SO_4. The organic phase was separated, and the aqueous phase was extracted with $CHCl_3$ (2 × 100 mL). The combined organic phases were dried (Na_2SO_4) and evaporated under reduced pressure to give a pale beige solid, which was applied for further transformations without purification. Yield: 10.84 g (77%).

(*R*)-6,7-Dimethoxy-2-(*tert*-butoxycarbonyl)-1,2,3,4-tetrahydro-1-isoquinolineacetic acid [compound **(*R*)-2**]
Compound (*R*)-**2** was prepared from (*R*)-6,7-dimethoxy-1,2,3,4-tetrahydro-1-isoquinolineacetic acid hydrochloride [(*R*)-**1** . HCl] according to the above procedure described for (±)-**2**, but using 2 equiv of NaOH. (*R*)-**2** was obtained as a pale beige solid (yield: 74%), which was applied for further transformations without purification.

(*S*)-6,7-Dimethoxy-2-(*tert*-butoxycarbonyl)-1,2,3,4-tetrahydro-1-isoquinolineacetic acid [compound **(*S*)-2**]
Compound (*S*)-**2** was prepared from (*S*)-6,7-dimethoxy-1,2,3,4-tetrahydro-1-isoquinolineacetic acid hydrochloride [(*S*)-**1** . HCl] according to the above procedure described for compound (±)-**2**, but using 2 equiv of NaOH. (*S*)-**2** was obtained as a pale beige solid (yield: 80%), which was applied for further transformations without purification.

***N*-Benzyl-6,7-dimethoxy-1,2,3,4-tetrahydro-1-isoquinolineacetamide hydrochloride** [compound **(±)-4**]
To a stirred and ice-salt bath-cooled solution of 6,7-dimethoxy-2-(*tert*-butoxycarbonyl)-1,2,3,4-tetrahydro-1-isoquinolineacetic acid [(±)-**2**, 2.11 g, 6 mmol)] and triethylamine (0.61 g, 6 mmol) in anhydrous toluene (25 mL), ethyl chloroformate (0.65 g, 6 mmol) was added dropwise at a rate to keep the internal temperature below –10 °C. After 5 min, a solution of benzylamine (0.64 g, 6 mmol) in anhydrous CH_2Cl_2 (15 mL) was added dropwise, while the internal temperature was kept below 0 °C. After the addition, the reaction mixture was warmed to boiling and heated under reflux for 5 min. When the mixture was cooled down to room temperature, EtOAc (80 mL) and water (40 mL) were added. The separated organic phase was washed successively with cold 5% HCl solution (2 × 40 mL), saturated $NaHCO_3$ solution (2 x 40 mL), and water (2 x 40 mL). After drying (Na_2SO_4), the solvent was evaporated in vacuo to yield a white solid residue, which was dissolved in MeOH (20 mL), and 22% ethanolic HCl (25 mL) was added. The mixture was stirred at room temperature for 12 h then evaporated. The crystalline residue was filtered off and recrystallized from MeOH–Et_2O
Yield: 1.47 g (65%), white crystalline substance, m.p.: 195-198 °C. ^1H NMR (500 MHz, D_2O): δ = 2.81-2.89 (1H, m), 2.93-3.04 (2H, m), 3.12-3.24 (2H, m), 3.53 (3H, s), 3.60-3.67 (1H, m), 3.78 (3H, s), 3.93 (1H, d, *J* = 15.2 Hz), 4.39 (1H, d, *J* = 15.2 Hz), 4.77-4.82 (1H, m), 6.66 (1H, s), 6.73-6.78 (3H, m), 7.09-7.21 (3H, m). ^{13}C NMR (125 MHz, D_2O): δ = 24.7,

36.7, 40.9, 42.6, 52.7, 55.5, 55.6, 108.6, 111.7, 121.6, 125.4, 126.8, 127.3, 128.5, 137.4, 147.3, 148.0, 171.5. Elemental analysis: calcd. (%) for $C_{20}H_{25}ClN_2O_3$ (376.88): C, 63.74; H, 6.69; Cl, 9.41; N, 7.43; found: C,63.60; H, 6.75; Cl, 9.27; N, 7.22.

(*R*)-*N*-Benzyl-6,7-dimethoxy-1,2,3,4-tetrahydro-1-isoquinolineacetamide hydrochloride [compound **(*R*)-4**]
Compound (*R*)-**4** was prepared from (*R*)-6,7-dimethoxy-2-(*tert*-butoxycarbonyl)-1,2,3,4-tetrahydro-1-isoquinoline-acetic acid [(*R*)-**3**] according to the above procedure described for compound (±)-**4**.
Yield: 1.29 g (57%), white crystalline substance, m.p.: 225-227 °C. The ^{1}H and ^{13}C NMR spectra of (*R*)-**4** were identical to those of (±)-**4**. Elemental analysis: calcd. (%) for $C_{20}H_{25}ClN_2O_3$ (376.88): C, 63.74; H, 6.69; Cl, 9.41; N, 7.43; found: C, 63.69; H, 6.52; Cl, 9.24; N, 7.31.

(*S*)-*N*-Benzyl-6,7-dimethoxy-1,2,3,4-tetrahydro-1-isoquinolineacetamide hydrochloride [compound **(*S*)-4**]
Compound (*S*)-**4** was prepared from (*S*)-6,7-dimethoxy-2-(*tert*-butoxycarbonyl)-1,2,3,4-tetrahydro-1-isoquinoline-acetic acid [(*S*)-**3**] according to the above procedure described for compound (±)-**4**.
Yield: 1.33 g (59%), white crystalline substance, m.p.: 226-228 °C. The ^{1}H and ^{13}C NMR spectra of (*R*)-**4** were identical to those of (±)-**4**. Elemental analysis: calcd. (%) for $C_{20}H_{25}ClN_2O_3$ (376.88): C, 63.74; H, 6.69; Cl, 9.41; N, 7.43; found: C, 63.83; H, 6.52; Cl, 9.37; N, 7.26.

***N*-Benzyl-6,7-dimethoxy-1,2,3,4-tetrahydro-1-isoquinolineethanamine dihydrochloride [**L1, compound **(±)-5**]
N-Benzyl-6,7-dimethoxy-1,2,3,4-tetrahydro-1-isoquinolineacetamide hydrochloride [(±)-**4**, 1.13 g, 3 mmol) was dissolved in water (15 mL), and the solution was made alkaline using 20% NaOH and extracted with $CHCl_3$ (4 × 20 mL). The combined organic extracts were dried (Na_2SO_4) and evaporated. The crude base was dissolved in THF (10 mL), and this solution was added dropwise to a cooled and stirred suspension of $LiAlH_4$ (0.38 g, 10 mmol) in THF (40 mL). The mixture was stirred under reflux for 5 h and then cooled. The excess of $LiAlH_4$ was decomposed by adding a mixture of water (0.8 mL) and THF (15 mL). The inorganic salts were filtered off and washed with EtOAc (3 × 50 mL). The combined organic filtrate and washings were dried (Na_2SO_4) and evaporated under reduced pressure to give a yellow oil. It was dissolved in MeOH and converted to a crystalline dihydrochloride salt using an excess of 22% ethanolic HCl and Et_2O. The crystalline product was filtered off and recrystallized from MeOH–Et_2O.
Yield: 0.65 g (54%), yellowish-white crystalline substance, m.p.: 254-256 °C. ^{1}H NMR (500 MHz, D_2O): δ = 2.31-2.46 (2H, m), 2.97-3.13 (3H, m), 3.16-3.25 (1H, m), 3.41-3.49 (1H, m), 3.50-3.59 (1H, m), 3.73 (3H, s), 3.84 (3H, s), 4.18-4.30 (2H, m), 4.60 (1H, t, *J* = 6.2 Hz), 6.66 (1H, *s*), 6.90 (1H, s), 7.34-7.47 (5H, m). ^{13}C NMR (125 MHz, D_2O): δ = 23.8, 29.3, 38.7, 42.3, 50.7, 52.2, 55.8, 56.0, 109.5, 112,3, 122.0, 124.5, 129.3, 129.8, 129.9, 147.2, 148.4. Elemental analysis: calcd. (%) for $C_{20}H_{28}Cl_2N_2O_2$ (399.35): C, 60.15; H, 7.07; Cl, 17.76; N, 7.01; found: C, 60.02; H, 6.95; Cl, 17.80; N, 6.86.

(*R*)-*N*-Benzyl-6,7-dimethoxy-1,2,3,4-tetrahydro-1-isoquinolineethanamine dihydrochloride [compound **(*R*)-5**]
Compound (*R*)-**5** was prepared from (*R*)-*N*-benzyl-6,7-dimethoxy-1,2,3,4-tetrahydro-1-isoquinolineacetamide hydrochloride [(*R*)-**4**] according to the above procedure described for (±)-**5**.
Yield: 0.62 g (52%), yellowish-white crystalline substance, m.p.: 242-246 °C, $[\alpha]_D^{20}$= +6.5 (c = 0.2, MeOH). The ^{1}H and ^{13}C NMR spectra of (*R*)-**5** were identical to those of (±)-**5**. Elemental analysis: calcd. (%) for $C_{20}H_{28}Cl_2N_2O_2$ (399.35): C, 60.15; H, 7.07; Cl, 17.76; N, 7.01; found: C, 59.91; H, 7.03; Cl, 17.54; N, 6.82.

(*S*)-*N*-Benzyl-6,7-dimethoxy-1,2,3,4-tetrahydro-1-isoquinolineethanamine dihydrochloride [compound **(*S*)-5**]
Compound (*S*)-**5** was prepared from (*S*)-*N*-benzyl-6,7-dimethoxy-1,2,3,4-tetrahydro-1-isoquinolineacetamide hydrochloride [(*S*)-**4**] according to the above procedure described for (±)-**5**.
Yield: 0.61 g (51%), yellowish-white crystalline substance, m.p.: 240-244 °C, $[\alpha]_D^{20}$= –7,0 (c = 0.2, MeOH). The ^{1}H and ^{13}C NMR spectra of (*S*)-**5** were identical to those of (±)-**5**. Elemental analysis: calcd. (%) for $C_{20}H_{28}Cl_2N_2O_2$ (399.35): C, 60.15; H, 7.07; Cl, 17.76; N, 7.01; found: C, 60.29; H, 6.86; Cl, 17.48; N, 7.14.

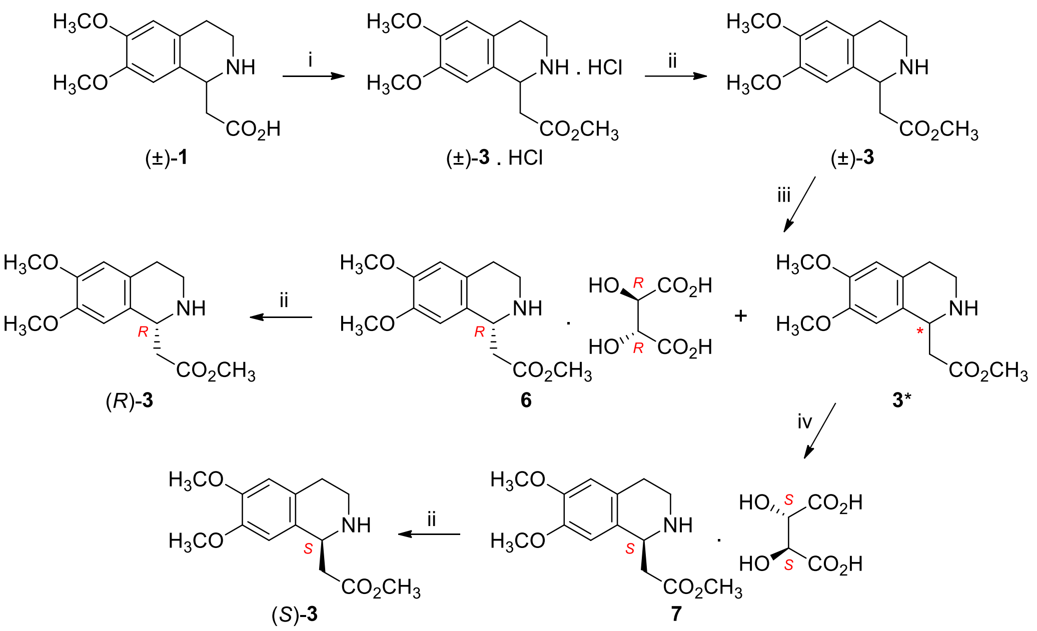

Scheme S2. Synthesis and resolution of compound (±)-**3**.
Reagents and conditions (i) $SOCl_2$ (1.1 equiv), MeOH, 0 °C, 2 h, then r.t., 2 h, then reflux, 6 h (96%); (ii) Na_2CO_3 (89-97%); (iii) L-(+)-tartaric acid (1 equiv), MeOH, r.t., 5 d (32%); (iv) D-(–)-tartaric acid (1 equiv), MeOH, r.t., 5 d (37%).

Methyl 6,7-dimethoxy-1,2,3,4-tetrahydro-1-isoquinolineacetate hydrochloride [compound (±)-3 . HCl]
Under stirring and ice-salt cooling, $SOCl_2$ (14.07 g, 118.7 mmol) was added dropwise to methanol (250 mL) at a rate to keep the temperature below –10 ° C. To the further stirred and ice-salt cooled mixture, 6,7-dimethoxy-1,2,3,4-tetrahydro-1-isoquinolineacetic acid [3] [compound (±)-**1**, 27.10 g, 107.8 mmol] was added in small portions. After the addition, the mixture was stirred in an ice-water bath for 2 h, at room temperature for 2 h, finally under reflux for 6 h. After adding Et_2O (150 mL), the mixture was allowed to stand at room temperature for 1 day. The separated crystals were filtered off and washed with a 1:1 mixture of MeOH and Et_2O. For analytical purposes, a sample was recrystallized from MeOH–Et_2O.
Yield: 31.33 g (96 %), yellowish white, shining crystals, m.p.: 217-220 °C (MeOH–Et_2O). ^{1}H NMR (500 MHz, D_2O): δ = 3.07-3.12 (2H, *m*), 3.26-3.32 (2H, m), 3.49-3.57 (1H, *m*), 3.64-3.73 (1H, *m*), 3.85 (3H, *s*), 3.88 (3H, *s*), 3.90 (3H, s), 4.98-5.04 (1H, *m*), 6.88 (1H, *s*), 6.96 (1H, *s*). ^{13}C NMR (125 MHz, D_2O): δ = 24.4, 37.3, 39.7, 51.6, 53.0, 56.0, 56.1, 109.2, 112.2, 122.6, 124.9, 147.7, 148.4, 172.6. Elemental analysis: calcd. (%) for $C_{14}H_{20}ClNO_4$ (301.77): C, 55.72; H, 6.68; Cl, 11.75; N, 4.64; found: C, 55.58; H, 6.51; Cl, 11.63; N, 4.50.

Methyl 6,7-dimethoxy-1,2,3,4-tetrahydro-1-isoquinolineacetate [compound (±)-3]
A solution of methyl 6,7-dimethoxy-1,2,3,4-tetrahydro-1-isoquinolineacetate hydrochloride [(±)-**3** . HCl, 30.00 g, 99.4 mmol] in water (150 mL) was made alkaline using a conc. aqueous Na_2CO_3 solution and was extracted with EtOAc (4 × 150 mL). The combined organic extracts were dried (Na_2SO_4) and evaporated. Yield: 25,49 g (97 %), beige solid. The crude base (±)-**3** was used for the resolution process without further purification.

Methyl (*R*)-6,7-dimethoxy-1,2,3,4-tetrahydro-1-isoquinolineacetate (2*R*,3*R*)-tartrate (compound 6)
Methyl 6,7-dimethoxy-1,2,3,4-tetrahydro-1-isoquinolineacetate [(±)-**3**, 25.88 g, 97.5 mmol) and (2*R*,3*R*)-(+)-tartaric acid (14.64 g, 97.5 mmol) were dissolved in methanol (250 mL) with heating, and the solution was allowed to stand at room temperature. The precipitated crystals were filtered off after 5 days and washed with methanol. For analytical purposes, a sample was recrystallized from MeOH. For further transformations, crude **6** was used without purification.
Yield: 12.83 g (32%), white crystalline substance, m.p: 152-156 °C (MeOH). ^{1}H NMR (500 MHz, D_2O): δ 2.94-3.08 (2H, m), 3.14-3.22 (2H, m), 3.36-3.45 (1H, m), 3.50-3.59 (1H, m), 3.70 (3H, s), 3.77 (3H, s), 3.79 (3H, s), 4.45 (2H, s), 4.86-4.93 (1H, m), 6.77 (1H, s), 6.85 (1H, s). ^{13}H NMR (125 MHz, D_2O): δ = 24.4, 37.2, 39.7, 51.6, 53.0, 56.0, 56.1, 73.0, 109.2, 112.2, 124.6, 124.9, 147.6, 148.4, 172.6, 176.4. Elemental analysis: calcd. (%) for $C_{18}H_{25}NO_{10}$ (415.39): C, 52.05; H, 6.07; N, 3.37; found: C, 52.00; H, 5.89; N, 3.16.

Methyl (*R*)-6,7-dimethoxy-1,2,3,4-tetrahydro-1-isoquinolineacetate [compound (*R*)-3]
A solution of methyl (*R*)-6,7-dimethoxy-1,2,3,4-tetrahydro-1-isoquinolineacetate (2*R*,3*R*)-tartrate [**6**, 10.00 g, 24 mmol] in water (100 mL) was made alkaline by using a conc. aqueous Na_2CO_3 solution and was extracted with EtOAc (4 × 100 mL). The combined organic extracts were dried (Na_2SO_4) and evaporated to yield a beige solid, which was applied for further transformations without purification. For analytical purposes, a sample was recrystallized from *i*Pr_2O.
Yield: 5.75 g (90 %), beige crystals, m.p.: 102-105 °C (lit. [4] m.p.: 106-107 °C), $[\alpha]_D^{20}$= +48,8 (c = 1.0, EtOH) (lit.[4] $[\alpha]_D^{20}$= +50,05 (c = 0.7, 95% EtOH)), *ee* ≥ 99%. The ^1H and ^{13}C NMR spectra ($CDCl_3$) of (*R*)-**3** were identical to that of its antipode [(*S*)-**3**] published [5] earlier. Elemental analysis: calcd. (%) for $C_{14}H_{19}NO_4$ (265.30): C, 63.38; H, 7.22; N, 5.28; found: C, 63.46; H, 7.11; N, 5.08.

Methyl (*S*)-6,7-dimethoxy-1,2,3,4-tetrahydro-1-isoquinolineacetate (2*S*,3*S*)-tartrate (compound 7)
After filtering off the(2*R*,3*R*)-tartrate **6**, the mother liquor was evaporated, and the residual solid was dissolved in water (200 mL). The solution was made alkaline (Na_2CO_3), and the base **3***, being rich in the (*S*)-isomer, was extracted with EtOAc (4 × 100 mL). After drying (Na_2SO_4) and evaporation, 16.99 g of **3*** was obtained, which was used directly for the preparation of the D-tartrate **7**.
The above-described base being rich in the (*S*)-isomer (**3***, 16,99 g, 64 mmol) and (2*S*,3*S*)-(–)-tartaric acid (9.61 g, 64 mmol) was dissolved in methanol (170 mL) with heating, and the solution was allowed to stand at room temperature. The precipitated crystals were filtered off after 5 days and washed with methanol. For analytical purposes, a sample was recrystallized from MeOH. For further transformations, crude **7** was used without purification.
Yield: 14.94 g [37%, starting from (±)-**3**], white crystalline substance, m.p: 153-156 °C (MeOH). The ^1H and ^{13}C NMR spectra of 7 were identical to those of **6**. Elemental analysis: calcd. (%) for $C_{18}H_{25}NO_{10}$ (415.39): C, 52.05; H, 6.07; N, 3.37; found: C, 51.93; H, 5.99; N, 3.26.

Methyl (*S*)-6,7-dimethoxy-1,2,3,4-tetrahydro-1-isoquinolineacetate [compound (*S*)-3]
Compound (*S*)-**3** was obtained from the (2*S*,3*S*)-tartrate **7** in the same manner as described above for the liberation of (*R*)-**3** from the (2*R*,3*R*)-tartrate **6**. (*S*)-**3** was obtained as a beige solid which was used for further transformations without purification. For analytical purposes, a sample was recrystallized from *i*Pr_2O.
Yield: 4.65 g (89 %), beige crystals, m.p.: 104-106 °C, $[\alpha]_D^{20}$= –48,8 (c = 1.0, EtOH), *ee* ≥ 99%. The ^1H and ^{13}C NMR spectra ($CDCl_3$) of (*S*)-**3** were identical to that published [5] earlier. Elemental analysis: calcd. (%) for $C_{14}H_{19}NO_4$ (265.30): C, 63.38; H, 7.22; N, 5.28; found: C, 63.17; H, 7.04; N, 5.11.

The *ee* values of (*R*)-**3** and (*S*)-**3** were determined by HPLC using a ChiralPak ODH column. The analytical conditions were as follows: eluent: a 90:10 mixture of *n*-hexane and *i*PrOH containing 0.1% Et_2N, flow rate: 0.5 mL min^{-1}, detection at 240 nm, retention times: (*S*)-**3**: ca. 45 min, (*R*)-**3**: ca. 60 min (Figure S21).

1.3. Synthesis of racemic Ligand 2 [L2, compound (±)-10] and its enantiomers [(*R*)-10and (*S*)-10]

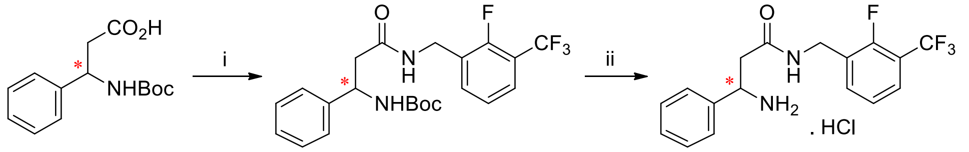

(±)-**8**, (*R*)-**8**, (*S*)-**8** (±)-**9**, (*R*)-**9**, (*S*)-**9** (±)-**10**, (*R*)-**10**, (*S*)-**10**

Scheme S3. Synthesis of compounds (±)-**10**, (*R*)-**10,** and (*S*)-**10**.
Reagents and conditions (i) 2-fluoro-3-(trifluoromethyl)benzylamine (1 equiv), HOBt (1.2 equiv), DIC (1.2 equiv), DMF, r.t, 16 h (82-86%); (ii) 22% HCl in EtOH, reflux, 4 h (83-85%).

***N*-(2-Fluoro-3-(trifluoromethyl)benzyl]-3-phenyl-3-[(*tert*-butoxycarbonyl)amino]propanamide [compound (±)-9]**
A mixture of (±)-3-(*tert*-butoxycarbonylamino)-3-phenylpropanoic acid [6] [(±)-**8**, 265 mg, 1 mmol], hydroxybenzo-triazole (HOBt, 162 mg, 1.2 mmol), *N,N'*-diisopropylcarbodiimide (DIC, 151 mg, 1.2 mmol), and 2-fluoro-3-(tri-fluoromethyl)benzylamine (193 mg, 1 mmol) was stirred in DMF (15 mL) at room temperature for 16 h. The solvent was evaporated, and the residue was purified by column chromatography over silica gel using EtOAc as eluent.

Yield: 378 mg (86%), white solid, m.p. = 170–173 °C. ^{1}H NMR (500 MHz, $CDCl_3$): δ = 1.39 (9H, s), 2.71-2.74 (2H, m), 4.35 (1H, dd, *J* = 5.7, 15.7 Hz), 4.43 (1H, dd, *J* = 6.2, 15.5 Hz), 4.98-5.07 (1H, m), 5.91 (1H, brs), 5.98 (1H, brs), 7.1 (1H, t, *J* = 7.9 Hz), 7.18-7.27 (6H, m), 7.48 (1H, t, *J* = 7.2 Hz). ^{13}C NMR (125 MHz, $CDCl_3$): δ = 28.3, 36.7, 36.8, 42.9, 79.7, 121.4, 124.0, 124.0, 126.0, 126.2, 126.3, 126.7, 126.8, 127.5, 128.6, 133.9, 134.0, 141.3, 155.3, 158.8, 170.4. Elemental analysis: calcd. (%) for $C_{22}H_{24}F_4N_2O_3$ (440,43): C, 59.99; H, 5.49; N, 6.36; found: C, 60.11; H, 5.28; N, 6.23.

(*R*)-*N*-(2-Fluoro-3-(trifluoromethyl)benzyl]-3-phenyl-3-[(*tert*-butoxycarbonyl)amino]propanamide [compound (*R*)-9]

Compound (*R*)-**9** was prepared starting from (*R*)-3-(*tert*-butoxycarbonylamino)-3-phenylpropanoic acid [7] [(*R*)-**8**] according to the above procedure described for the racemic analog (±)-**9**.

Yield: 374 mg (85%), white solid, m.p: 171-174 °C, $[\alpha]^{20}_{D}$= +22.3 (c = 0.5, MeOH), *ee* ≥ 99%. The ^{1}H and 13C NMR spectra of (*R*)-**9** were identical to those of (±)-**9**. Elemental analysis: calcd. (%) for $C_{22}H_{24}F_4N_2O_3$ (440,43): C, 59.99; H, 5.49; N, 6.36; found: C, 60.15; H, 5.33; N, 6.27.

(*S*)-*N*-(2-Fluoro-3-(trifluoromethyl)benzyl]-3-phenyl-3-[(*tert*-butoxycarbonyl)amino]propanamide [compound (*S*)-9]

Compound (*S*)-**9** was prepared starting from (*S*)-3-(*tert*-butoxycarbonylamino)-3-phenylpropanoic acid [8] [(*S*)-**8**] according to the above procedure described for the racemic analog (±)-**9**.

Yield: 352 mg (82%), white solid, m.p: 170-174 °C, $[\alpha]^{20}_{D}$= –19.6 (c = 0.5, MeOH), *ee* ≥ 99%. The ^{1}H and 13C NMR spectra of (*R*)-**9** were identical to those of (±)-**9**. Elemental analysis: calcd. (%) for $C_{22}H_{24}F_4N_2O_3$ (440,43): C, 59.99; H, 5.49; N, 6.36; found: C, 60.22; H, 5.41; N, 6.38.

The *ee* values of (*R*)-**9** and (*S*)-**9** were determined by HPLC using Phenomenex-IA column. The analytical conditions were as follows: eluent: a mixture of *n*-hexane and *i*PrOH (95:5), flow rate: 0.5 mL min^{-1}, detection at 210 nm, retention times: (*R*)-**9**: 24.49 min, (*S*)-**9**: 25.73 min) (Figure S22).

3-Amino-*N*-(2-fluoro-3-(trifluoromethyl)benzyl]-3-phenylpropanamide hydrochloride [compound (±)-10]

N-(2-Fluoro-3-(trifluoromethyl)benzyl]-3-phenyl-3-[(*tert*-butoxycarbonyl)amino]propanamide [(±)-**9**, 440 mg, 1 mmol] was refluxed in 22% ethanolic HCl (15 mL) for 4 h. The solvent was evaporated, and the crystalline residue was filtered off from Et_2O.

Yield: 321 mg (85%), white solid, m.p. 180–183 °C. ^{1}H NMR (500 MHz, D_2O): δ = 3.09 (1H, dd, *J* = 9.3, 14.7 Hz), 3.16 (1H, dd, *J* = 5.3, 14.4 Hz), 4.29 (1H, d, *J* = 15.2 Hz), 4.49 (1H, d, *J* = 15.7 Hz), 4.78-4.83 (1H, m), 7.13 (1H, t, *J* = 7.3 Hz), 7.24 (1H, t, *J* = 7.2 Hz), 7.42-7.52 (4H, m), 7.68 (1H, t, *J* = 7,4 Hz). ^{13}C NMR (125 MHz, D_2O): δ = 36.6, 36.6, 39.7, 52.6, 124.5, 124.5, 126.0, 126.1, 126.5, 126.6, 127.1, 129.4, 129.9, 133.8, 133.9, 134.5. 170.8. Elemental analysis: calcd. (%) for $C_{17}H_{17}ClF_4N_2O$ (376.78): C, 54.19; H, 4.55; Cl, 9.41; N, 7.44; found: C, 54.28; H, 4.47; Cl, 9.62, N, 7.63.

(*R*)-3-Amino-*N*-(2-fluoro-3-(trifluoromethyl)benzyl]-3-phenylpropanamide hydrochloride [compound (*R*-10]

Compound (*R*)-**10** was prepared starting from (*R*)-*N*-(2-fluoro-3-(trifluoromethyl)benzyl]-3-phenyl-3-[(*tert*-butoxy-carbonyl)amino]propanamide [(*R*)-**9**] according to the above procedure described for the racemic analog (±)-**10**.

Yield: 316 mg (84%), white solid, m.p.: 186-189 °C, $[\alpha]^{20}_{D}$= +2.3 (c = 0.5, MeOH). The ^{1}H and ^{13}C NMR spectra of (*R*)-**10** were identical to those of (±)-**10**. Elemental analysis: calcd. (%) for $C_{17}H_{17}ClF_4N_2O$ (376.78): C, 54.19; H, 4.55; Cl, 9.41; N, 7.44; found: C, C, 54.25; H, 4.62; Cl, 9.57, N, 7.31.

(*S*)-3-Amino-*N*-(2-fluoro-3-(trifluoromethyl)benzyl]-3-phenylpropanamide hydrochloride [compound (*S*-10]

Compound (*S*)-**10** was prepared starting from (*S*)-*N*-(2-fluoro-3-(trifluoromethyl)benzyl]-3-phenyl-3-[(*tert*-butoxy-carbonyl)amino]propanamide [(*S*)-**9**] according to the above procedure described for the racemic analog (±)-**10**.

Yield: 312 mg (83%), white solid, m.p.: 185-189 °C, $[\alpha]^{20}_{D}$= –1.8 (c = 0.5, MeOH) The ^{1}H and ^{13}C NMR spectra of (*S*)-**10** were identical to those of (±)-**10** Elemental analysis: calcd. (%) for $C_{17}H_{17}ClF_4N_2O$ (376.78): C, 54.19; H, 4.55; Cl, 9.41; N, 7.44; found: C, 54.26; H, 4.61; Cl, 9.52, N, 7.58.

1.4. Synthesis of racemic Ligand 3 [L3, compound (±)-12] and its enantiomers [(*R*)-12 and (*S*)-12]

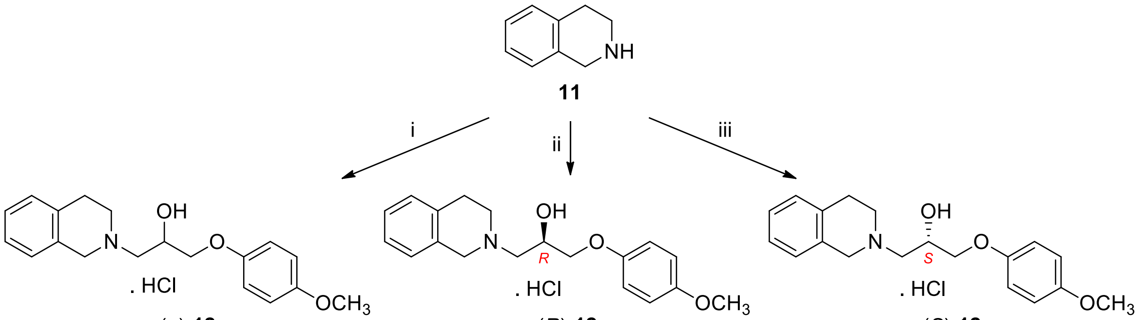

(±)-**12** (*R*)-**12** (*S*)-**12**

Scheme S4. Synthesis of compounds (±)-**12**, (*R*)-**12,** and (*S*)-**12**.
Reagents and conditions: (i) 1. *O*-(4-methoxyphenyl)glycidol (1 equiv), EtOH, reflux, 20 h, 2. 22% HCl in EtOH, MeOH, Et_2O (60%); (ii) 1. (*R*)-*O*-(4-methoxyphenyl)glycidol (1 equiv), EtOH, reflux, 20 h, 2. 22% HCl in EtOH, MeOH, Et_2O (48%); (iii) (*S*)-*O*-(4-methoxyphenyl)glycidol (1 equiv), EtOH, reflux, 20 h, 2. 22% HCl in EtOH, MeOH, Et_2O (45%)

1-[(4-Methoxyphenoxy)methyl]-2-(1,2,3,4-tetrahydroisoquinolin-2-yl)ethanol [compound (±)-12]
A solution of 1,2,3,4-tetrahydroisoquinoline (**11**, 133 mg, 1 mmol) and *O*-(4-methoxyphenyl)glycidol (180 mg, 1 mmol) in EtOH (10 mL) was heated under reflux for 20 h. Then the solution was evaporated, and the crude base was converted to its hydrochloride by adding 22% ethanolic HCl and Et_2O. The crystals were filtered off and recrystallized from EtOH.
Yield: 210 mg (60%), white crystalline substance, m.p. = 150-152 °C. ^1H NMR (500 MHz, D_2O): δ = 3.16-3.23 (2H, m), 3.43-3.52 (2H, m), 3.67 (2H, brs), 3.76 (3H, s), 4.03 (1H, dd, *J* = 10.3, 5.1 Hz), 4.08 (1H, dd, *J* = 10.3, 4.2 Hz), 4.44-4.56 (3H, m), 6.91-6.98 (4H, m), 7.19 (1H, d, *J* = 7.4 Hz), 7.26-7.36 (m, 3H). ^{13}C NMR (125 MHz, D_2O): δ = 24.3, 55.9, 57.2, 64.0, 70.4, 115.2, 116.2, 126.8, 127.1, 128.4, 128.8, 130.7, 152.3, 153.7. Elemental analysis: calcd. (%) for $C_{19}H_{24}ClNO_3$ (349.85): C, C, 65.23; H, 6.91; Cl, 10.13; N, 4.00; found: C, 65.02; H, 6.77; Cl, 9.98; N, 3.86.

(*R*)-1-[(4-Methoxyphenoxy)methyl]-2-(1,2,3,4-tetrahydroisoquinolin-2-yl)ethanol [compound (*R*)-12]
Compound (*R*)-**12** was prepared according to the above procedure described for the racemic analog (±)-**12** by using (*R*)-*O*-(4-methoxyphenyl)glycidol instead of the racemic epoxide. The crude base was purified by column chromatography on aluminum oxide using a 1:1 mixture of *n*-hexane and EtOAc as eluent before converting to its hydrochloride.
Yield: 168 mg (48%), white crystalline substance, m.p. = 171-173 °C. $[\alpha]_D^{20}$= +20.0 (c = 0.5, MeOH). The ^1H and ^{13}C NMR spectra of (*R*)-**12** were identical to those of (±)-**12**. Elemental analysis: calcd. (%) for $C_{19}H_{24}ClNO_3$ (349.85): C, C, 65.23; H, 6.91; Cl, 10.13; N, 4.00; found: C, 64.98; H, 7.06; Cl, 10.12; N, 3.79.

(*S*)-1-[(4-Methoxyphenoxy)methyl]-2-(1,2,3,4-tetrahydroisoquinolin-2-yl)ethanol [compound (*S*)-12]
Compound **(***S***)**-**12** was prepared according to the above procedure described for the **(***R***)-12** by using (*S*)-*O*-(4-methoxyphenyl)glycidol instead of the racemic epoxide.
Yield: 157 mg (45%), white crystalline substance, m.p. = 171-174 °C. $[\alpha]_D^{20}$= –20.0 (c = 0.5, MeOH). The ^1H and ^{13}C NMR spectra of (*S*)-**12** were identical to those of (±)-**12**. Elemental analysis: calcd. (%) for $C_{19}H_{24}ClNO_3$ (349.85): C, C, 65.23; H, 6.91; Cl, 10.13; N, 4.00; found: C, 65.13; H, 6.84; Cl, 10.09; N, 4.07.

1.5. Synthesis of Ligand 4 [L4, compound (±)-14]

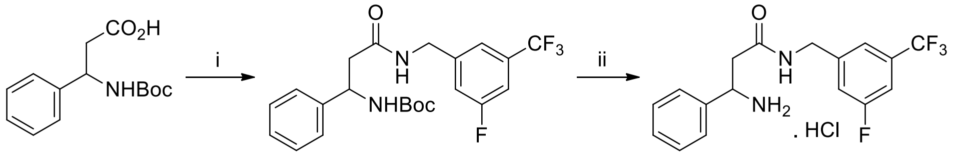

(±)-**8** (±)-**13** (±)-**14**

Scheme S5. Synthesis of compound (±)-**14**.
Reagents and conditions (i) 3-fluoro-5-(trifluoromethyl)benzylamine (1 equiv), HOBt (1.2 equiv), DIC (1.2 equiv), DMF, r.t, 16 h (81%); (ii) 22% HCl in EtOH, reflux, 4 h (85%).

N-(3-Fluoro-5-(trifluoromethyl)benzyl]-3-phenyl-3-[(*tert*-butoxycarbonyl)amino]propanamide [compound (±)-13]

Compound(±)-**13** was prepared according to the procedure described for the regioisomeric analog (±)-**9** by using 3-fluoro-5-(trifluoromethyl)benzylamine instead of the 2-fluoro-3-(trifluoromethyl) isomer.
Yield: 356 mg (81%), white solid, m.p. 175–178 °C. ^{1}H NMR (500 MHz, $CDCl_3$): δ = 1.39 (9H, s), 2.66-2,87 (2H, m), 4.29 (1H, dd, *J* = 5.7, 15.5 Hz), 4.41 (1H, dd, *J* = 5.6, 15.8 Hz), 5.05 (1H, brs), 5.91 (1H, d, *J* = 6.9 Hz), 6.14 (1H, brs), 6.92 (1H, d, *J* = 8.9 Hz), 7.13-7.34 (2H, m), 7.23-7.34 (6H, m). ^{13}C NMR (125 MHz, $CDCl_3$): δ = 28.3, 42.6, 43.0, 111.7, 111.8, 111.9, 112.0, 117.9, 118.0, 119.9, 120.0, 126.0, 127.7, 128.8, 141.9, 142.0, 155.5, 161.6, 163.6. Elemental analysis: calcd. (%) for $C_{22}H_{24}F_4N_2O_3$ (440.43): C, 59.99; H, 5.49; N, 6.36; found: C, 60.29; H, 5.41; N, 6.61.

3-Amino-*N*-(3-fluoro-5-(trifluoromethyl)benzyl]-3-phenylpropanamide hydrochloride [compound (±)-14]

Compound(±)-**14** was prepared according to the procedure described for the regioisomeric analog (±)-**10**.
Yield: 320 mg (85%), white solid, m.p. 180–182 °C. ^{1}H NMR (500 MHz, D_2O): δ = 2.98 (1H, dd, *J* = 9.5, 15.0 Hz), 3.16 (1H, dd, *J* = 5.7, 14.4 Hz), 4.12 (1H, d, *J* = 15.5 Hz), 4.33 (1H, d, *J* = 15.5 Hz), 4.67 (1H, m), 6.80 (1H, d, *J* = 9.2 Hz), 7.18 (1H, s), 7.26-7.47 (6H, m). ^{13}C NMR (125 MHz, D_2O): δ = 36.6, 36.7, 39.7, 52.6, 124.5, 124.6, 126.0, 126.1, 126.5, 126.6, 127.1, 129.4, 129.9, 133.8, 133.9, 134.5. 170.8. Elemental analysis: calcd. (%) for $C_{17}H_{17}ClF_4N_2O$ (376.78): C, 54.19; H, 4.55; Cl, 9.41; N, 7.44; found: C, 54.27; H, 4.51; Cl, 9.62; N, 7.31.

1.6. Synthesis of Ligand 6 [L6, compound (±)-16]

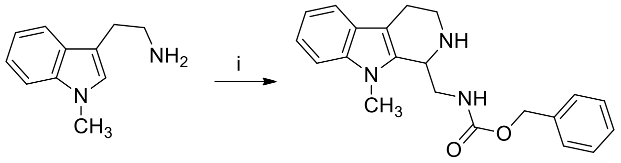

15 (±)-**16**

Scheme S6. Synthesis of compound (±)-**16**.
Reagents and conditions (i) $(MeO)_2CHCH_2NHCbz$ (0.75 equiv), AcOH, H_2O, reflux under N_2, 2 h (76%).

***N*-Benzyloxycarbonyl-(9-methyl-2,3,4,9-tetrahydro-1*H*-pyrido[3,4-*b*]indol-1-yl)methanamine [compound (±)-16]**

A mixture of 4.36 g (16 mmol) (1-methyl-1*H*-indol-3-yl)ethanamine [9] (**15**) and 4.47 g (12 mmol) benzyl *N*-(2,2-dimethoxyethyl)carbamate [10] in AcOH (65 mL) and H_2O (28 mL) was refluxed under N_2 for 2 h and then evaporated. The crude oily product was purified by column chromatography on silica using a 4:1 mixture of toluene and MeOH as eluent. For analytical purposes, a sample was recrystallized from *i*Pr_2O–EtOAc.
Yield: 4.98 g (76%), pale brown powder, m.p.: 118.5-119.5 °C. ^{1}H NMR (500 MHz, $CDCl_3$): δ = 1.75 (2H, s), 2.72-2,73 (2H, m), 3.02-3.08 (1H, m), 3.15-3.21 (2H, m), 3.74 (3H, s), 4.11 (1H, d, *J* = 7.9 Hz), 5.11-5.17 (2H, m), 5.65 (1H, s), 7.09 (1H, t, *J* = 7.3 Hz), 7.21 (1H, t, *J* = 7.3 Hz), 7.28-7.38 (6H, m), 7.48 (1H, d, *J* = 7.3 Hz). ^{13}C NMR (125 MHz, $CDCl_3$): δ = 22.6, 29.9, 38.1, 43.0, 50.7, 66.8, 108.8, 108.9, 118.1, 119.1, 121.6, 126.7, 128.1, 128.2, 128.6, 134.2, 136.5, 137.2, 156.7. Elemental analysis: calcd. (%) for $C_{21}H_{23}N_3O_2$ (349.43): C, 72.18; H, 6.63; N, 12.03; found: C, 71.95; H, 6.38; N, 11.91.

1.7. Synthesis of Ligand 8 [L8, compound (±)-19]

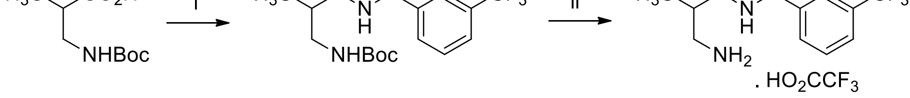

(±)-**17** (±)-**18** (±)-**19**

Scheme S7. Synthesis of compound (±)-**19**.
Reagents and conditions (i) 2-fluoro-3-(trifluoromethyl)benzylamine (1 equiv), HOBt (1.2 equiv), DIC (1.2 equiv), DMF, r.t., 16 h; (ii) TFA, CH_2Cl_2, r.t., 3 h (85%, 2 steps).

***N*-(2-Fluoro-3-(trifluoromethyl)benzyl]-2-methyl-3-[(*tert*-butoxycarbonyl)amino]propanamide [compound (±)-18]**

3-[(*tert*-Butoxycarbonyl)amino]-2-methylpropanoic acid [11] [(±)-**17**] was converted to the corresponding 2-fluoro-3-(trifluoromethyl)benzylamide [(±)-**18** according to the procedure described for the phenyl-substituted analog (±)-**9**. The crude product (±)-**18** was used in the next step without purification.

3-Amino-*N*-(2-fluoro-3-(trifluoromethyl)benzyl]-2-methylpropanamide trifluoroacetate [compound (±)-19]
A mixture of the Boc-protected amide [([(±)-**18**, 400 mg, 0.9 mmol] in CH_2Cl_2 (30 mL) with TFA (2 mL) was stirred at room temperature for 3 h. The solvent was evaporated, and the residue was crystallized with Et_2O.
Yield: 300 mg (85%), white crystalline substance, m.p. 140-143 °C. ^1H NMR (500 MHz, D_2O): δ =1.19 (3H, d, *J* = 7.1 Hz), 2.74-2.84 (1H, m), 3.00-3.10 (1H, m), 4.38 (1H, d, *J* = 15.6 Hz), 4.53 (1H, d, *J* = 15.4 Hz), 7.28 (1H, t, *J* = 7.3 Hz), 7.56 (1H, t, *J* = 7.0 Hz), 7.63 (1H, t, *J* = 7.0 Hz). ^{13}C NMR (125 MHz, D_2O): δ = 15.1, 36.9, 37.0, 37.7, 41.4, 124.4, 124.5, 126.6, 126.7, 133.9, 134.0, 176.8. Elemental analysis: calcd. (%) for $C_{14}H_{15}F_7N_2O_3$ (392.27): C, 42.83; H, 3.82; N, 7.13; found: C, 42.67; H, 4.01; N, 7.32.

1.8. Synthesis of Ligand 9 [L9, compound (±)-21]

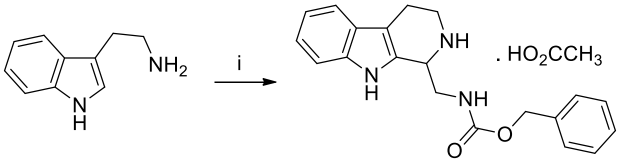

20 (±)-**21**

Scheme S8. Synthesis of compound (±)-**21**.
Reagents and conditions (i) $(MeO)_2CHCH_2NHCbz$ (0.75 equiv), AcOH, H_2O, reflux under N_2, 2 h (48%).

***N*-Benzyloxycarbonyl-(2,3,4,9-tetrahydro-1*H*-pyrido[3,4-*b*]indol-1-yl)methanamine [(±)-21]**
A mixture of tryptamine (**20**, 4.01 g, 25 mmol) and benzyl *N*-(2,2-dimethoxyethyl)carbamate [10] (4.47 g, 18.7 mmol) in AcOH (65 mL) and H_2O (28 mL) was refluxed under N_2 for 2 h. A dark solution was formed, which was evaporated, and the residue was treated with a 1:1 mixture of EtOH and H_2O (100 mL) to give a brown precipitate which was filtered off and recrystallized from MeOH.
Yield: 3.55 g (48%), white powder. m.p.: 145-148 °C. ^1H NMR (400 MHz, D_2O): δ = 1.91 (3H, s), 2.6 (2H, t, *J* = 5.2 Hz), 2.85-2.91 (1H, m), 3.10-3.25 (2H, m), 3.61-3.66 (3H, m), 4.05-4.07 (1H, m), 5.06 (2H, s), 6.93-6.97 (1H, dt, *J* = 7.5, 1.0 Hz), 7.03 (1H, dt, *J* = 7.5, 1.0 Hz), 7.22 (1H, brs), 7.28-7.38 (7H, m), 10.76 (1H, s). ^{13}C NMR (100 MHz, DMSO-d_6): δ = 22.0, 22.9, 41.6, 44.9, 52.8, 66.2, 109.0, 111.9, 118.3, 119.1, 121.4, 127.7, 128.6, 129.2, 134.9, 136.7, 138.1, 157.2, 172.9. Elemental analysis: calcd. (%) for $C_{22}H_{25}N_3O_4$ (395.45): C, 66.82; H, 6.37; N, 10.63; found: C, 66.69; H, 6.15; N, 10.48.

1.9. Synthesis of Ligand 10 [L10, compound (±)-24]

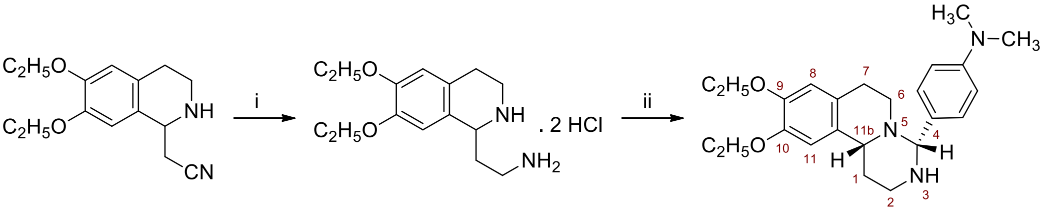

(±)-**22** (±)-**23** (±)-**24**

Scheme S9. Synthesis of compound (±)-**24**.
Reagents and conditions (i) H_2, RaNi, NH_3, MeOH, 50 °C, 50 atm, 8 h, 2. 22% HCl in EtOH, MeOH, Et_2O (73%); (ii) 1. NaOH, 2. 4-$(Me_2N)C_6H_4CHO$, MeOH, r.t., 3 h (64%)

6,7-Diethoxy-1,2,3,4-tetrahydro-1-isoquinolineethanamine dihydrochloride [compound **(±)-23**]
A mixture of 6,7-diethoxy-1,2,3,4-tetrahydro-1-isoquinolineacetonitrile [12] [(±)-**22**, 13.02 g, 50 mmol], MeOH (120 mL), 25% NH_3 in MeOH (120 mL), and Raney-Ni (5.0 g) was hydrogenated in an autoclave at 50 °C and 50 atm for 8 h. The catalyst was then filtered off, and the solvent evaporated. The oily residue was dissolved in MeOH (20 mL) and converted to dihydrochloride salt with 22% ethanolic HCl (25 mL) and Et_2O (120 mL). The crystals were filtered off and recrystallized from a mixture of MeOH–Et_2O.

Yield: 12.30 g (73%), white crystalline substance, m.p.: 245–248 °C. ^{1}H NMR (500 MHz, D_2O): δ = 1.34 (6H, t, *J* = 7.0 Hz), 2.33-2.43 (2H, m), 2.95-3.10 (2H, m), 3.11-3.24 (2H, m), 3.39-3.47 (1H, m), 3.52-3.60 (1H, m), 4.04-4.15 (4H, m), 4.63 (1H, t, *J* = 6.2 Hz), 6.83 (1H, s), 6.90 (1H, s). ^{13}C NMR (125 MHz, D_2O): δ = 13.7, 13.8, 24.0, 30.9, 35.9, 38.9, 52.4, 64.9, 65.3, 111.1, 113.4, 122.5, 114.7, 146.6, 147.7. Elemental analysis: calcd. (%) for $C_{15}H_{26}Cl_2N_2O_2$ (337.29): C, 53.41; H, 7.77; Cl, 21.02; N, 8.31; found: C, 53.23; H, 7.68; Cl, 20.84; N, 8.09.

(4*R,11b*R**)-9,10-Diethoxy-4-[4-(dimethylamino)phenyl]-1,3,4,6,7,11b-hexahydro-2*H*-pyrimido[6,1-*a*]isoquinoline** [compound **(±)-24**]

The above dihydrochloride [(±)-**23**] was converted to the corresponding diamine base in the usual manner (treatment with 10% NaOH, extraction with CH_2Cl_2, drying, and evaporation under reduced pressure). The diamine base (793 mg, 3 mmol) and 4-(dimethylamino)benzaldehyde (447 mg, 3 mmol) were dissolved in abs. MeOH (25 mL), and the mixture was allowed to stand at room temperature for 3 h. The solvent was then evaporated off and the oily product crystallized on treatment with Et_2O. The crystals were filtered off and recrystallized from *i*Pr_2O–EtOAc.

Yield: 760 mg (64%), white needles, m.p.: 113-116°C. ^{1}H NMR (500 MHz, $CDCl_3$): δ = 1.38-1.45 (6H, m), 1.65-1.78 (2H, m), 2.12-2.24 (2H, m), 2.43-2.54 (1H, m), 2.79-2.90 (2H, m), 2.94 (6H, s), 2.99-3.10 (1H, m), 3.30 (1H, d, *J* = 12.2 Hz), 3.57 (1H, d, *J* = 10.6 Hz), 4.00-4.10 (4H, m), 4.12 (1H, s), 6.57 (1H, s), 6.69-6.77 (3H, m), 7.33 (2H, d, *J* = 8.1 Hz). ^{13}C NMR (125 MHz, $CDCl_3$): δ = 14.9, 15.0, 29.1, 32.1, 40.6, 45.8, 46.3, 62.6, 64.5, 65.2, 81.4, 111.3, 112.6, 113.9, 127.7, 128.0, 130.1, 130.8, 146.8, 147.5, 150.4. Elemental analysis: calcd. (%) for $C_{24}H_{33}N_3O_2$ (395.54): C, 72.88; H, 8.41; N, 10.62; found: C, 73.01; H, 8.26; N, 10.57.

1.10. Synthesis of Ligand 11 [L11, compound (±)-28]

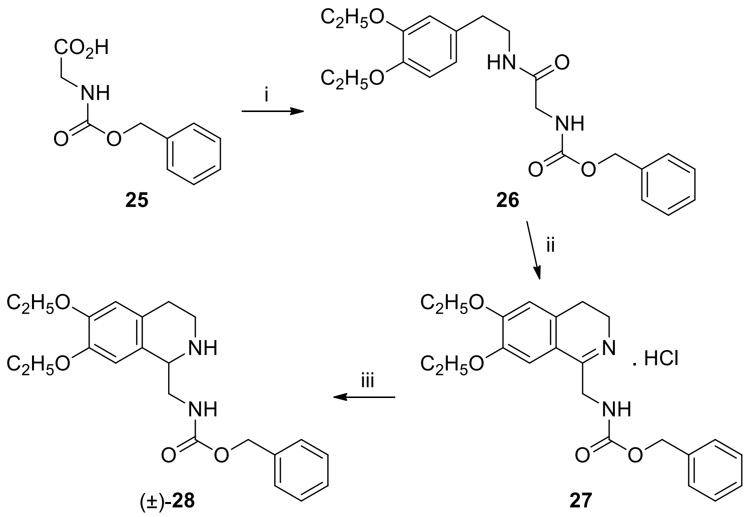

Scheme S10. Synthesis of compound (±)-**27**.

Reagents and conditions (i) 1. $ClCO_2Et$ (1 equiv), Et_3N (1 equiv), 3,4-$(EtO)_2C_6H_3(CH_2)_2NH_2$, (1 equiv), toluene–$CH_2Cl_2$, −10 °C to reflux (78%); (ii) 1. $POCl_3$, (3 equiv), $CHCl_3$, reflux, 3 h, 2. 22% HCl in EtOH, MeOH, Et_2O (72%); (iii) $NaBH_4$ (4 equiv), MeOH, r.t., 6 h (79%).

2-[(Benzyloxycarbonyl)amino]-*N*-[2-(3,4-diethoxyphenyl)ethyl]acetamide (26)

To a stirred and salt-ice cooled solution of *N*-(benzyloxycarbonyl)glycine (**25**, 2.09 g, 10 mmol) and triethylamine (1.01 g, 10 mmol) in anhydrous toluene (30 mL), ethyl chloroformate (1.09 g, 10 mmol.) was added dropwise at a rate to keep the internal temperature below −10 °C. After 5 min, a solution of 2-(3,4-diethoxyphenyl)ethylamine (2.09 g, 10 mmol) in dry CH_2Cl_2 (15 mL) was added dropwise to the stirred reaction mixture, while the internal temperature was kept below 0 °C. Then the mixture was heated to reflux and refluxed for 5 min. The mixture was allowed to cool to room temperature and washed with saturated aqueous $NaHCO_3$ solution (50 mL) and water (2 × 50 mL) after the addition of $CHCl_3$ (75 mL). The combined organic phases were dried (Na_2SO_4) and evaporated in vacuo to give an oily product, crystallized on treatment with Et_2O (30 mL). Crystals were filtered off and recrystallized from *i*Pr_2O–EtOAc.

Yield: 3.12 g (78%), white solid, m.p.: 79-81 °C. ^{1}H NMR (500 MHz, $CDCl_3$): δ = 1.39-1.44 (6H, m), 2.72 (2H, t, *J* = 6.4 Hz), 3.47-3.51 (2H, m), 3.80 (2H, d, *J* = 5.7 Hz), 4.03-4.09 (4H, m), 5.11 (2H, s), 5.35 (1H, brs), 5.97 (1H, brs), 6.66-6.70 (2H, m), 6.79 (1H, d, *J* = 8.1 Hz), 7.30-7.37 (5H, m). ^{13}C NMR (125 MHz, $CDCl_3$): δ = 14.9, 35.1, 40.7, 44.7, 64.6, 64.7,

67.2, 113.9, 114.2, 120.8, 128.1, 128.3, 131.2, 136.1, 147.5, 148.9, 168.7. Elemental analysis: calcd. (%) for $C_{22}H_{28}N_2O_5$ (400.47): C, 65.98; H, 7.05; N, 7.00; found: C, 65.72; H, 6.88; N, 6.81.

1-{[(Benzyloxycarbonyl)amino]methyl}-6,7-dimethoxy-3,4-dihydroisoquinoline hydrochloride (27)

To the stirred solution of 2-[(benzyloxycarbonyl)amino]-*N*-[2-(3,4-diethoxyphenyl)ethyl]acetamide (**26**, 3.00 g, 7.5 mmol) in dry $CHCl_3$ (40 mL), $POCl_3$ (3.45 g, 22.5 mmol) was added, then the solution was refluxed for 3 hours. After cooling to room temperature, the solvent was evaporated in vacuo, and the oily residue was dissolved carefully in H_2O (40 mL). The solution was made alkaline with 20% aqueous NaOH and extracted with $CHCl_3$ (3 × 50 mL). The combined organic phases were dried (Na_2SO_4) and evaporated in vacuo to give a beige solid, which was dissolved in MeOH (10 mL) and converted to hydrochloride salt with 22% ethanolic HCl (5 mL) and Et_2O (50 mL). The crystals were filtered off and recrystallized from a mixture of MeOH–Et_2O.

Yield: 2.26 g (72%), white crystalline substance, m.p.: 208-210 °C. ^1H NMR (500 MHz, D_2O): δ = 1.34 (3H, t, *J* = 7.1 Hz), 1.40 (3H, t, *J* = 7.1 Hz), 2.78 (1H, brs), 3.00 (2H, t, *J* = 7.4 Hz), 3.63 (1H, s), 3.72-3.82 (2H, m), 4.01-4.05 (2H, m), 4.70 (4H, s), 5.08 (2H, brs), 6.93-7.32 (7H, m). ^{13}C NMR (125 MHz, D_2O): δ = 13.6, 13.7, 24.5, 41.1, 65.4, 65.6, 67.7, 112.1, 112.7, 115.3, 127.8, 128.3, 128.5, 128.7, 135.1, 135.9, 146.7, 155.8. Elemental analysis: calcd. (%) for $C_{22}H_{27}ClN_2O_4$ (418.91): C, 63.08; H, 6.50; Cl, 8.46; N, 6.69; found: C, 63.23; H, 6.25; Cl, 8.31; N,6.46.

1-{[(Benzyloxycarbonyl)amino]methyl}-6,7-dimethoxy-1,2,3,4-tetrahydroisoquinoline [(±)-28]

To the stirred and ice-cooled suspension of 1-{[(benzyloxycarbonyl)amino]methyl}-6,7-dimethoxy-3,4-dihydroiso-quinoline hydrochloride (**27**, 2.09 g, 5 mmol) in MeOH (30 mL), $NaBH_4$ (0.76 g, 20 mmol) was added in small portions. The reaction mixture was stirred at room temperature for 6 h and then evaporated. The solid residue was dissolved in 3% aqueous HCl (30 mL), and the solution was extracted with $CHCl_3$ (3 × 50 mL) after made alkaline with a concentrated aqueous solution of NaOH. An orange oily product was obtained, which crystallized on treatment with 1:1 mixture of *n*-hexane and Et_2O (25 mL). The crystals were filtered off and recrystallized from *i*Pr_2O–EtOAc.

Yield 1.52 g (79%) white crystalline substance, m.p.: 90-92 °C. ^1H NMR δ (500 MHz, $CDCl_3$): δ = 1.39-1.44 (6H, m), 2.60-2.68 (2H, m), 2.95-3.10 (2H, m), 3.25-3.31 (1H, m), 3.60-3.69 (1H, m), 3.90-4.11 (5H, m), 5.10 (2H, s), 5.40 (1H, brs), 6.57 (1H, s), 6.65 (1H, s), 7.28-7.35 (5H, m). ^{13}C NMR (125 MHz, $CDCl_3$): δ = 14.9, 29.2, 39.7, 45.1, 54.7, 64.6, 64.8, 66.6, 111.6, 114.2, 127.9, 128.1, 128.5, 136.6, 147.2, 147.6, 156.6. Elemental analysis: calcd. (%) for $C_{22}H_{28}N_2O_4$ (384.47): C, 68.73; H, 7.34; N, 7.29; found: C, 68.49; H, 7.17; N, 7.04.

1.11. Synthesis of Ligand 12 [L12, compound (±)-30]

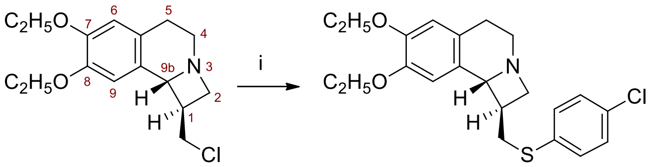

(±)-**29** (±)-**30**

Scheme S11. Synthesis of compound (±)-**30**.

Reagents and conditions (i) 4-ClC_6H_4SH, NaOH (1 equiv), MeOH, reflux, 2 h (85%)

(1*R,9b*R**)-1-{[(4-Chlorophenyl)thio]methyl}-7,8-diethoxy-1,4,5,9b-tetrahydro-2*H*-azeto[2,1-*a*]isoquinoline [(±)-30]**

To a solution of 1.47 g (5 mmol) (1*R**,9b*R**)-1-(chloromethyl)-7,8-diethoxy-2,4,5,9b-tetrahydro-1*H*-azeto[2,1-*a*]iso-quinoline [(±)-**29**] [13] in abs. EtOH (30 mL), methanolic NaOH (0.2 g, 5 mmol in 4 mL MeOH), and 4-chloro-thiophenol (0.72 g, 5 mmol) were added and the mixture was refluxed for 2 h. After cooling, the formed NaCl was filtered off, and the filtrate was evaporated. The oily residue was crystallized on treatment with Et_2O (20 mL). The crystals were filtered off and recrystallized from *i*Pr_2O.

Yield: 1,72 g (85%), beige crystals, m.p. 75-78 °C ^1H NMR (500 MHz, $CDCl_3$): δ = 1.39-1.46 (6H, m), 2.23-2.33 (1H, m), 2.3-2.46 (1H, m), 2.70-2.78 (1H, m) 2.82-2.88 (1H, m), 2.91-3.00 (1H, m), 3.15 (1H, dd, *J* = 3.4, 8.3 Hz), 3.34-3.54 (3H, m), 3.92-4.03 (2H, m), 4.06 (2H, q, *J* = 6.99 Hz), 4.47 (1H, d, *J* = 2.3 Hz), 6.55 (1H, s) 6.62 (1H, s), 7.20-7.30 (4H, m). ^{13}C NMR (125 MHz, $CDCl_3$): δ =14.8, 14.9, 22.4, 38.8, 40.8, 40.8, 44.7, 50.9, 63.8, 64.6, 64.7, 111.6, 113.7, 126.6, 129.1, 129.7, 130.7, 132.1, 134.6, 147.3, 147.9. Elemental analysis: calcd. (%) for $C_{22}H_{26}ClNO_2S$ (403.97): C, 65.41; H, 6.49; Cl, 8.78; N, 3.47; found. C, 65.73; H, 6.21; Cl, 8.53; N, 3.39.

References

1. Miklós, F.; Fülöp, F. Preparation of Spiropiperidines in Water. *Acta Chim. Slov.* **2009**, *56*, 674–679.
2. Szakonyi, Z.; Csillag, K.; Fülöp, F. Stereoselective Synthesis of Carane-Based Aminodiols as Chiral Ligands for the Catalytic Addition of Diethylzinc to Aldehydes. *Tetrahedron Asymmetry* **2011**, *22*, 1021–1027, doi:10.1016/j.tetasy.2011.06.013.
3. Pelletier, J.C.; Cava, M.P. The Reaction of 3,4-Dihydroisoquinolines with Malonic Acid and Its Derivatives. *Synthesis* **1987**, *1987*, 474–477, doi:10.1055/s-1987-33431.
4. Yao, Zhang-yu; Wu, Xiao-ming; Sun, Hong-bin Attempted Asymmetric Synthesis of Optically Pure Tetrabenazine. *J. China Pharm. Univ.* **2010**, *41*, 321–325.
5. Murahashi, S.-I.; Imada, Y.; Kawakami, T.; Harada, K.; Yonemushi, Y.; Tomita, N. Enantioselective Addition of Ketene Silyl Acetals to Nitrones Catalyzed by Chiral Titanium Complexes. Synthesis of Optically Active *β*-Amino Acids. *J. Am. Chem. Soc.* **2002**, *124*, 2888–2889, doi:10.1021/ja0176649.
6. Kumar, V.; Jaggi, M.; Singh, A.T.; Madaan, A.; Sanna, V.; Singh, P.; Sharma, P.K.; Irchhaiya, R.; Burman, A.C. 1,8-Naphthyridine-3-Carboxamide Derivatives with Anticancer and Anti-Inflammatory Activity. *Eur. J. Med. Chem.* **2009**, *44*, 3356–3362, doi:10.1016/j.ejmech.2009.03.015.
7. Thornburg, C.K.; Walter, T.; Walker, K.D. Biocatalysis of a Paclitaxel Analogue: Conversion of Baccatin III to *N*-Debenzoyl- *N*-(2-Furoyl) Paclitaxel and Characterization of an Amino Phenylpropanoyl CoA Transferase. *Biochemistry* **2017**, *56*, 5920–5930, doi:10.1021/acs.biochem.7b00912.
8. Marianacci, O.; Micheletti, G.; Bernardi, L.; Fini, F.; Fochi, M.; Pettersen, D.; Sgarzani, V.; Ricci, A. Organocatalytic Asymmetric Mannich Reactions with *N*-Boc and *N*-Cbz Protected *α*-Amido Sulfones (Boc:Tert-Butoxycarbonyl, Cbz: Benzyloxycarbonyl). *Chem. - Eur. J.* **2007**, *13*, 8338–8351, doi:10.1002/chem.200700908.
9. Lygin, A.V.; de Meijere, A. Synthesis of 1-Substituted Benzimidazoles from *o*-Bromophenyl Isocyanide and Amines. *Eur. J. Org. Chem.* **2009**, 5138–5141, doi:10.1002/ejoc.200900820.
10. Hooper, J.F.; Seo, S.; Truscott, F.R.; Neuhaus, J.D.; Willis, M.C. A-Amino Aldehydes as Readily Available Chiral Aldehydes for Rh- Catalyzed Alkyne Hydroacylation. *J Am Chem Soc* **2016**, 138, 1630–1634.
11. Moumne, R.; Lavielle, S.; Karoyan, P. Efficient Synthesis of β^2-Amino Acid by Homologation of *α*-Amino Acids Involving the Reformatsky Reaction and Mannich-Type Imminium Electrophile. *J. Org. Chem.* **2006**, *71*, 3332–3334, doi:10.1021/jo060316a.
12. Fülöp, F.; Wamhoff, H.; Sohár, P. Imidazo[1′,2′:3,4]Pyrimido[6,1-*a*]Isoquinoline and Pyrimido[1′,2′:3,4]Pyrimido[6,1-a]Isoquinoline Ring Systems by Tandem Cyclization. *Synthesis* **1995**, *1995*, 863–867, doi:10.1055/s-1995-3989.
13. Kóbor, J.; Fülöp, F.; Bernáth, G.; Sohár, P. Synthesis and Stereochemical Investigation of *cis*- and *trans*-1-Substituted 7,8-Dimethoxy-1,4,5,9b-Tetrahydro-2H-Azeto[2,1-*a*]Isoquinolines. *Tetrahedron* **1987**, *43*, 1887–1894, doi:10.1016/S0040-4020(01)81501-5.

2. ^{1}H and ^{13}C NMR spectra of new compounds

Figure S1. ^{1}H and ^{13}C NMR spectra of (*R*)-**1**. HCl

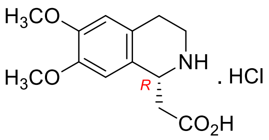

(*R*)-**1** . HCl

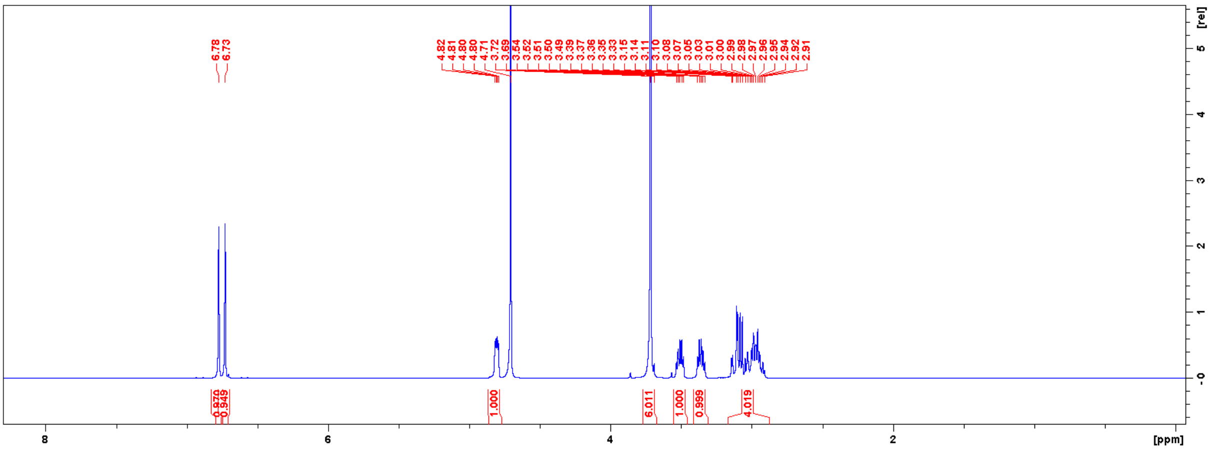

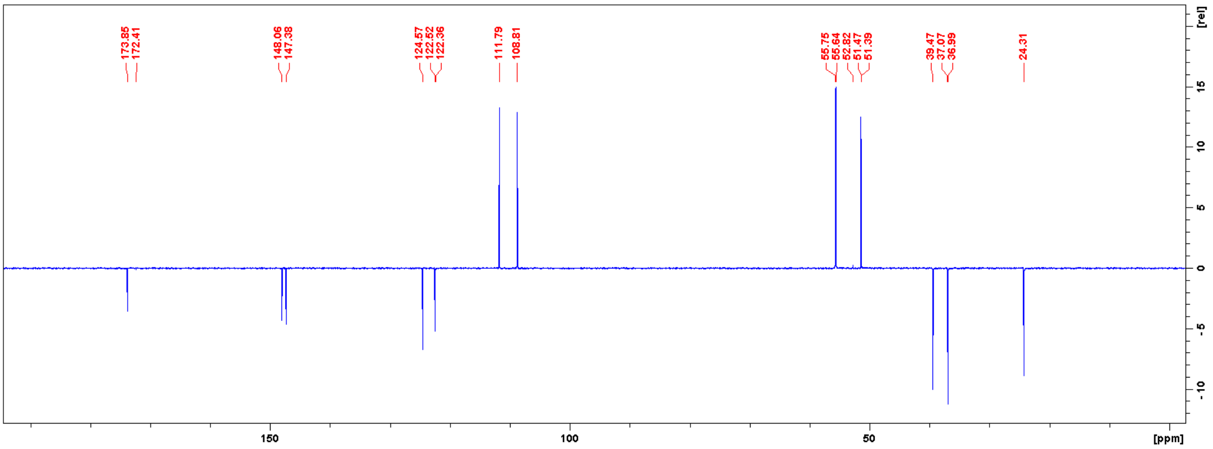

Figure S2. ^{1}H and ^{13}C NMR spectra of (±)-**3** . HCl

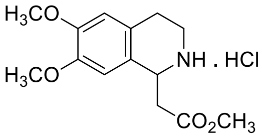

(±)-**3** . HCl

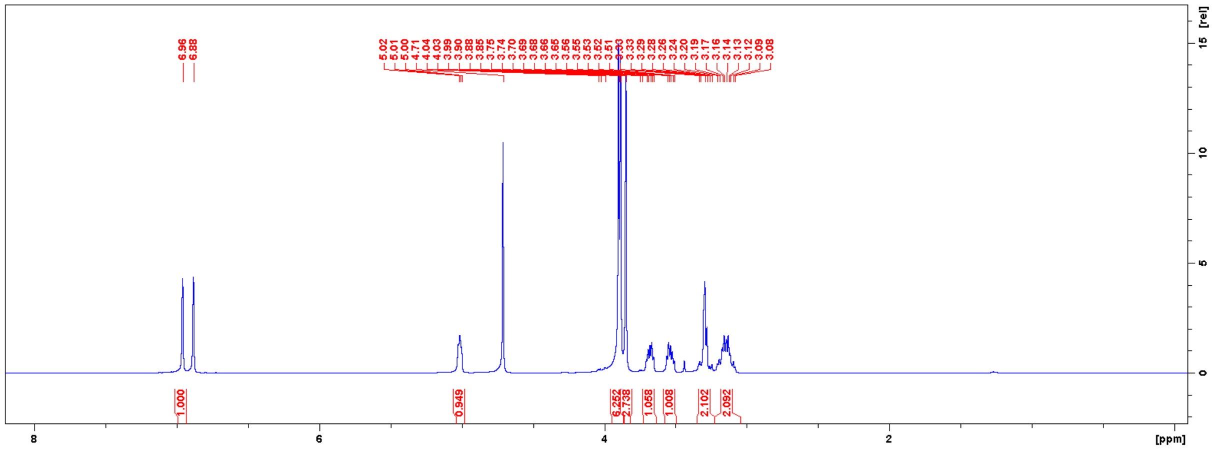

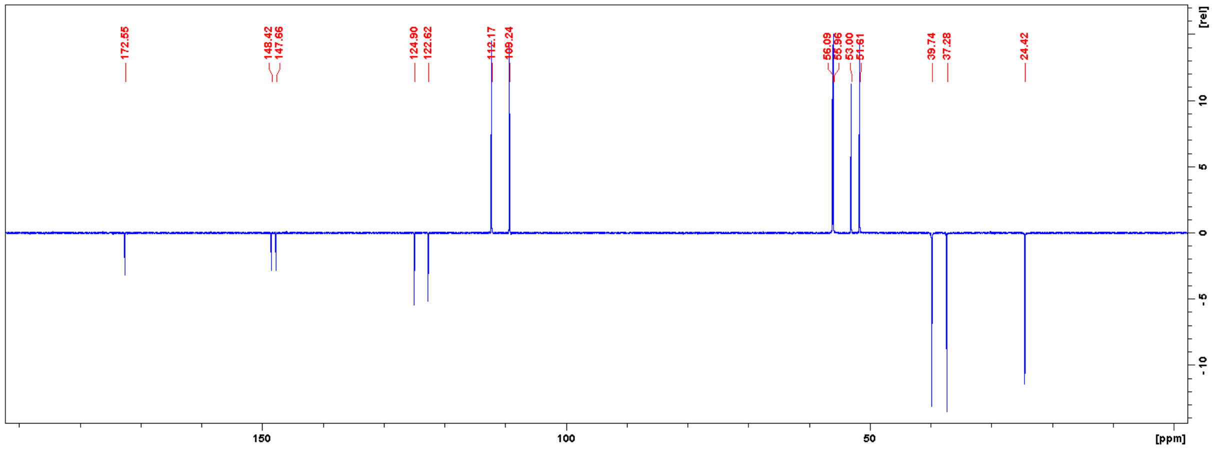

Figure S3. ^{1}H and ^{13}C NMR spectra of (±)-**4**

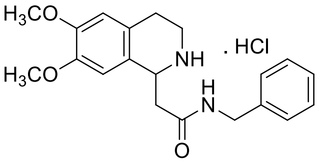

(±)-**4**

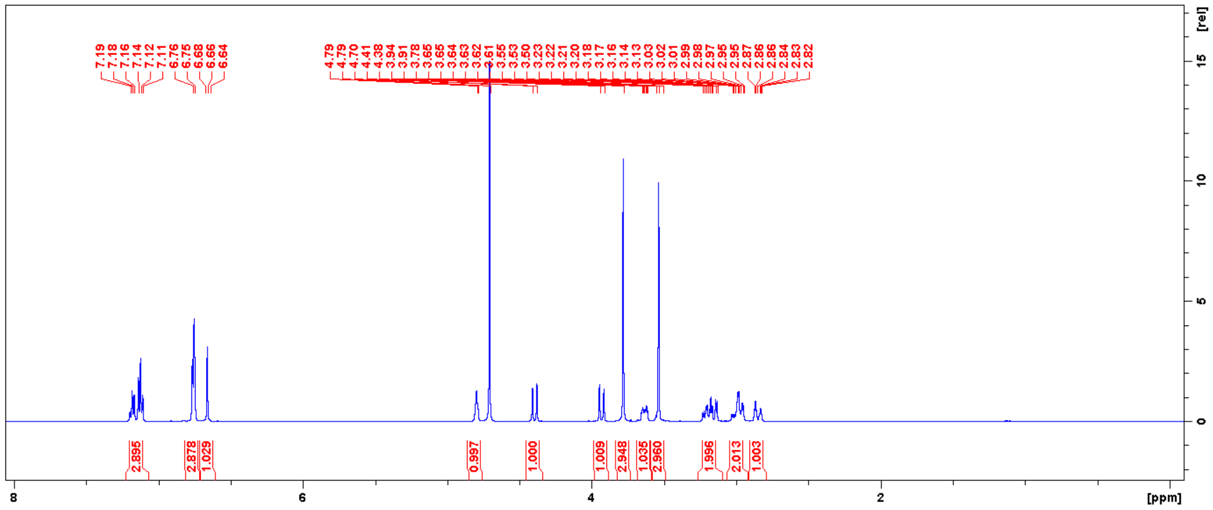

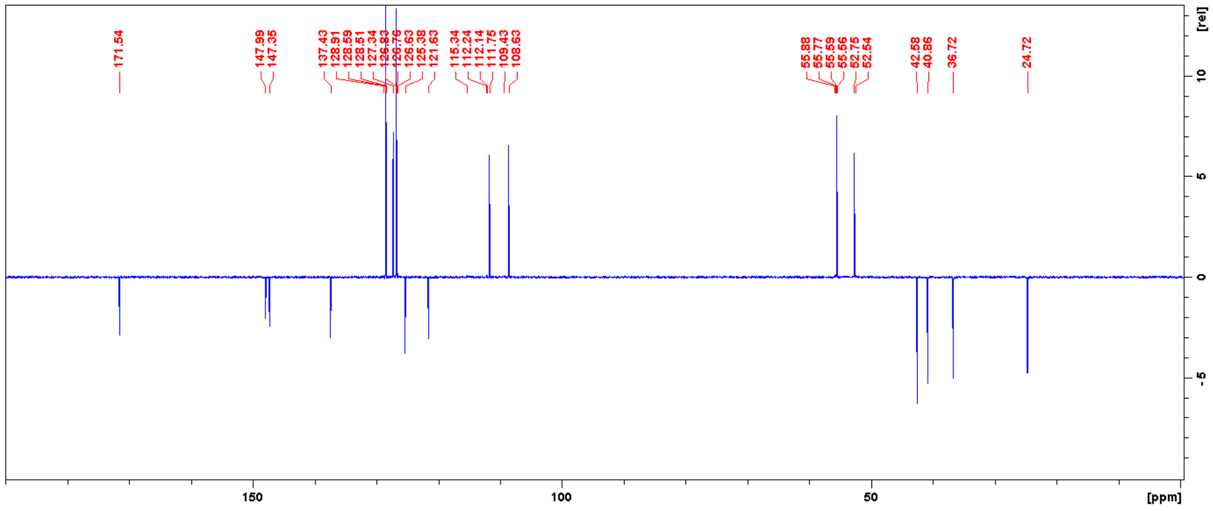

Figure S4. ^{1}H and ^{13}C NMR spectra of (±)-**5**

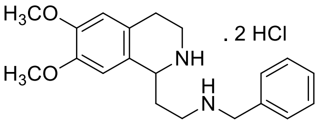

(±)-**5**

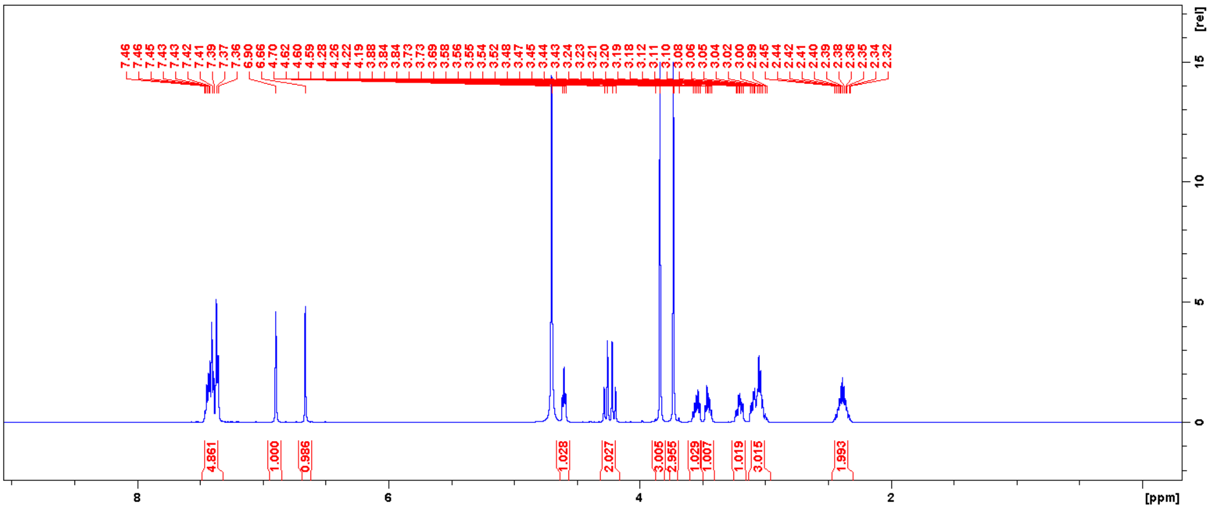

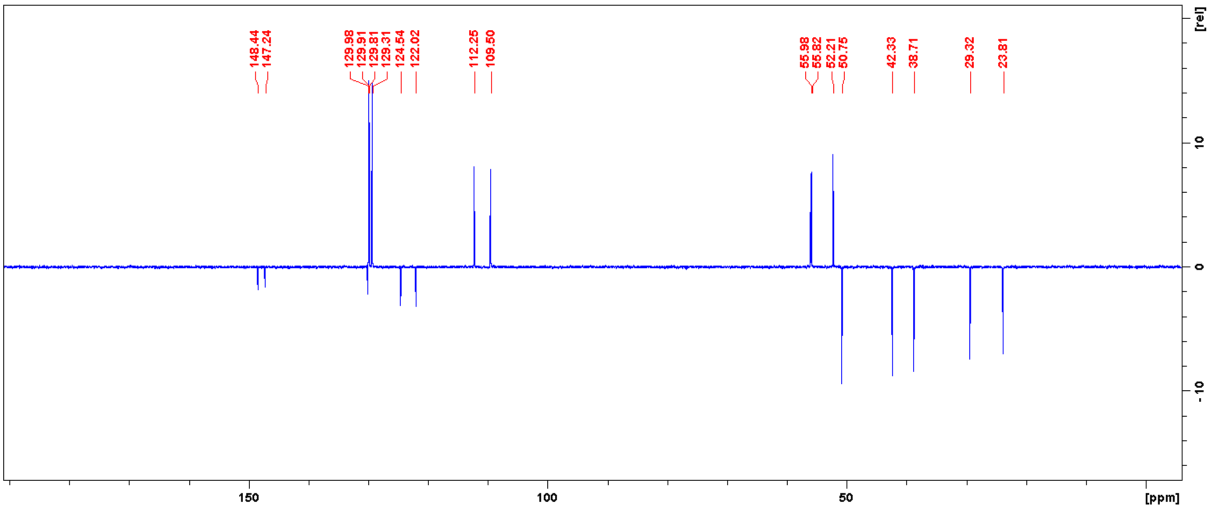

Figure S6. ^{1}H and ^{13}C NMR spectra of **6**

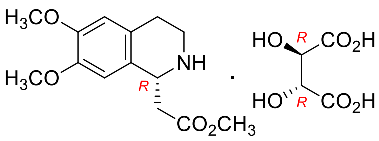

6

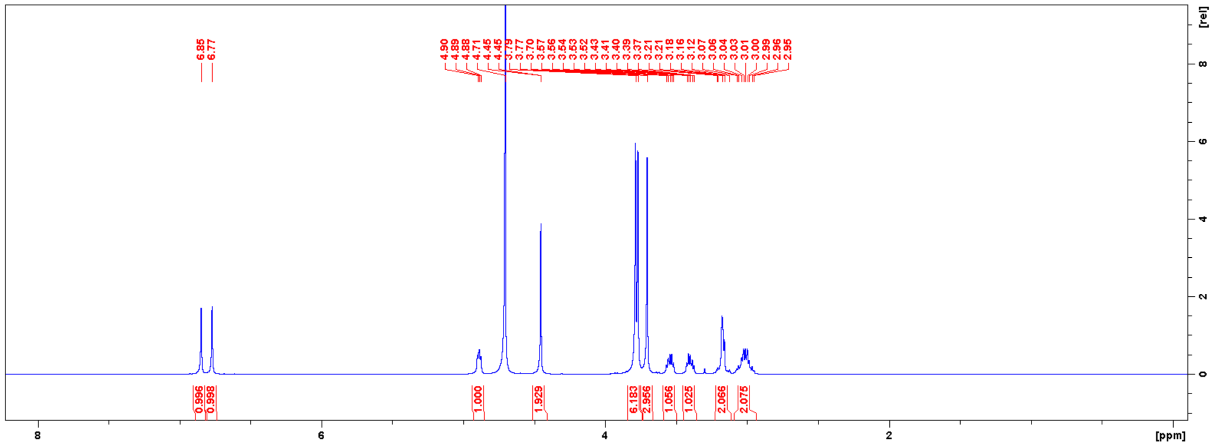

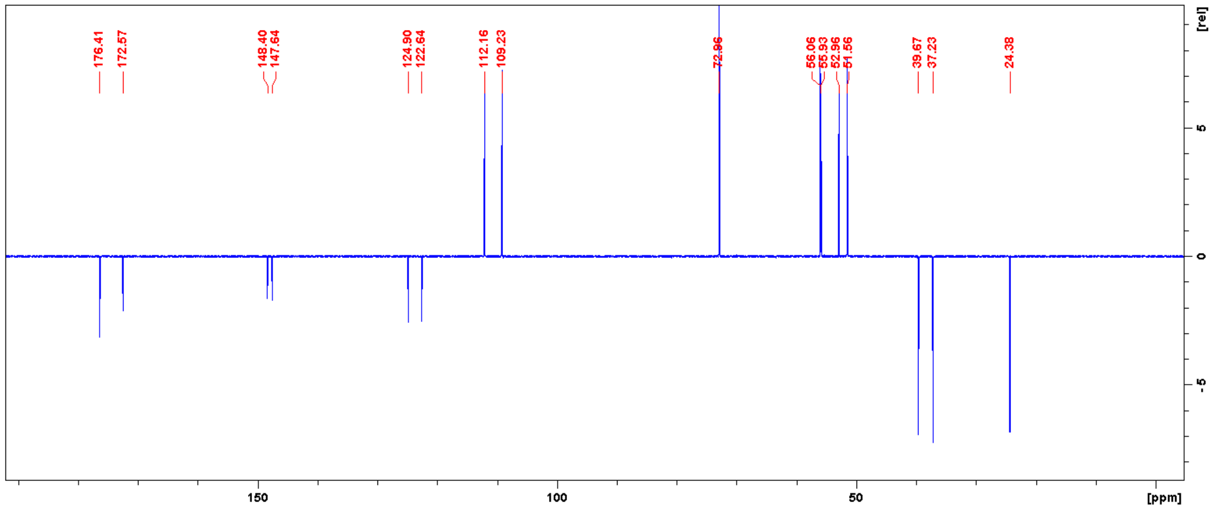

Figure S7. ^{1}H and ^{13}C NMR spectra of (±)-**9**

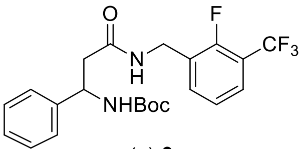

(±)-**9**

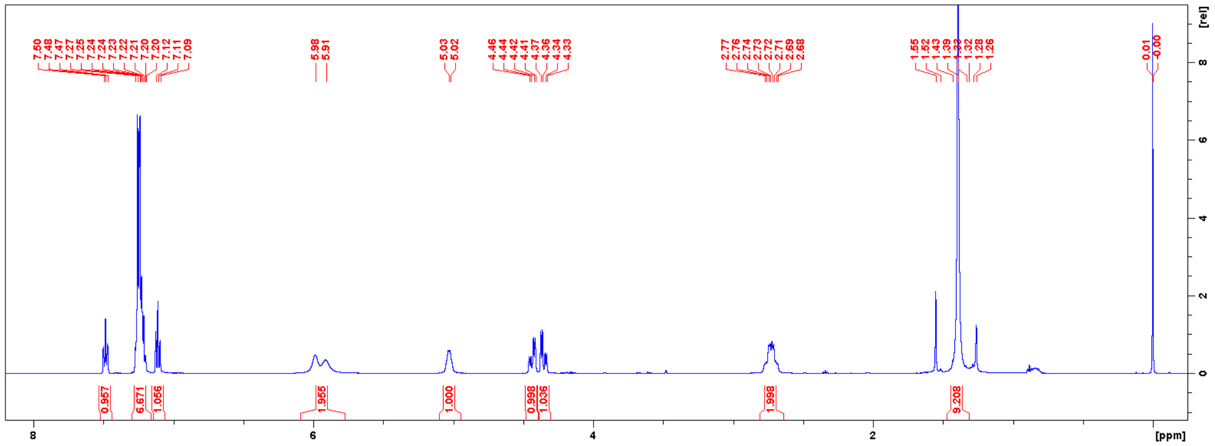

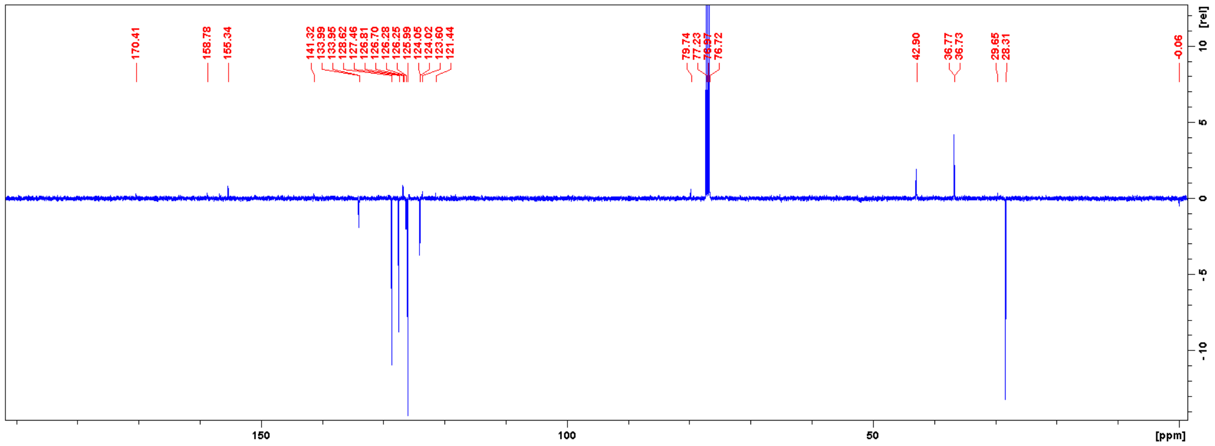

Figure S8. ^{1}H and ^{13}C NMR spectra of (±)-**10**

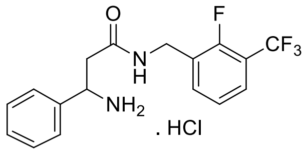

(±)-**10**

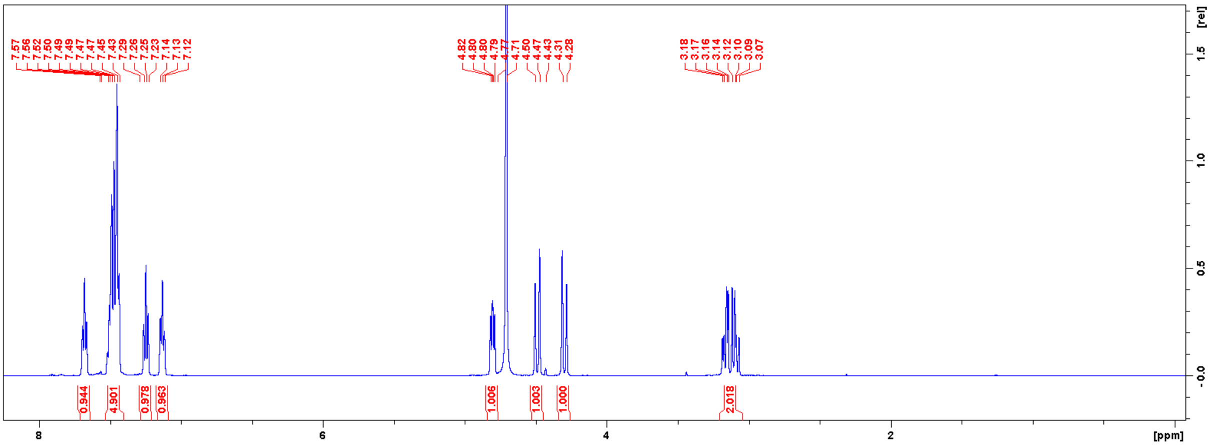

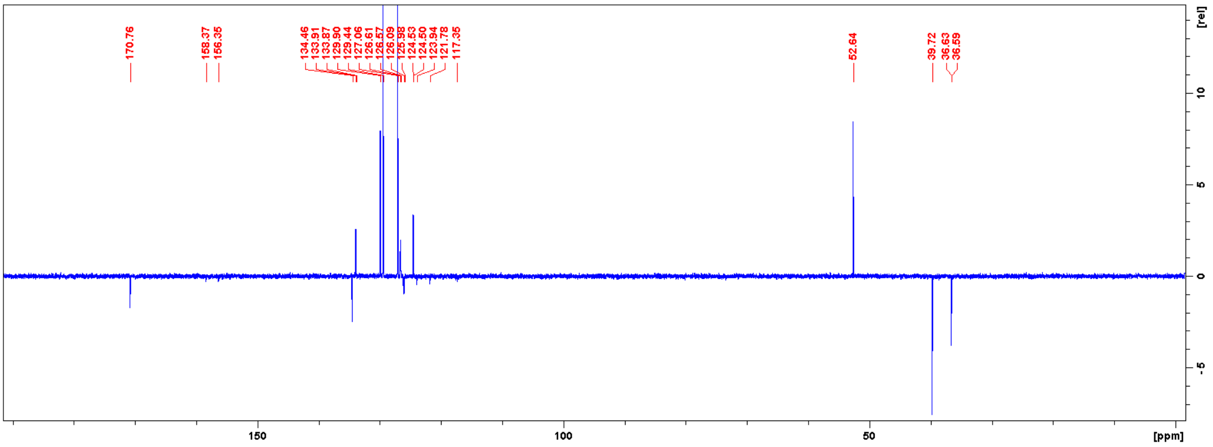

Figure S9. ^{1}H and ^{13}C NMR spectra of (±)-**12**

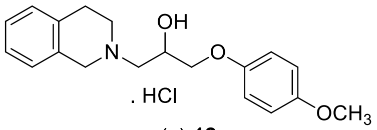

(±)-**12**

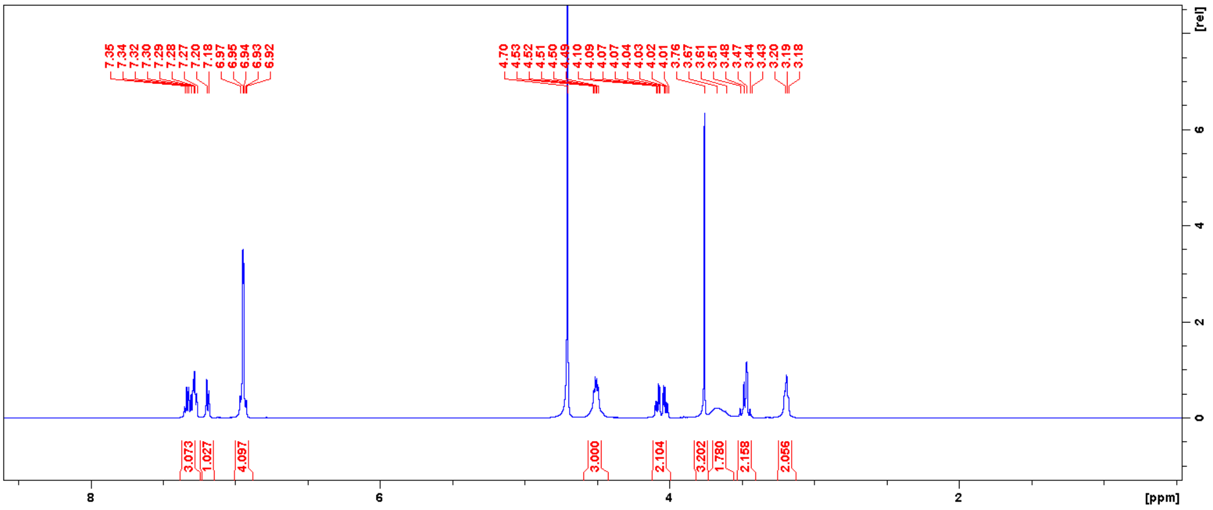

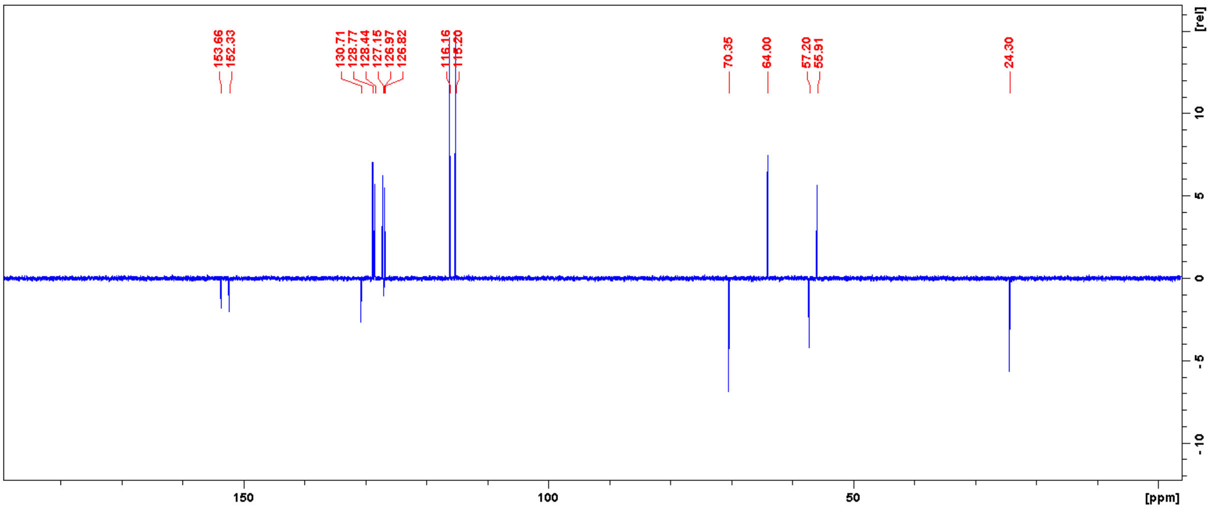

Figure S10. ^{1}H and ^{13}C NMR spectra of (±)-**13**

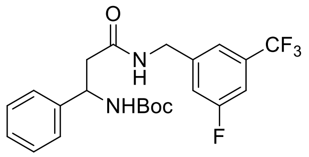

(±)-**13**

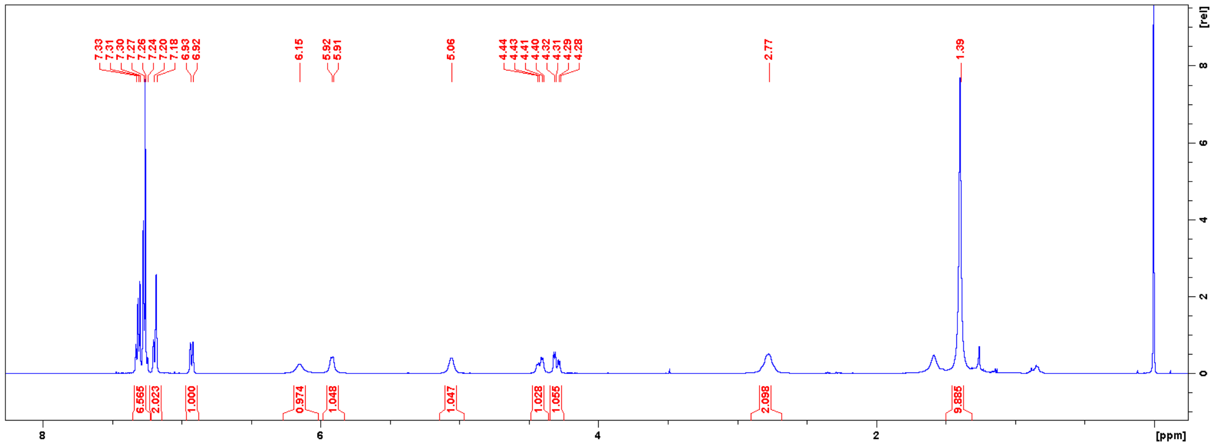

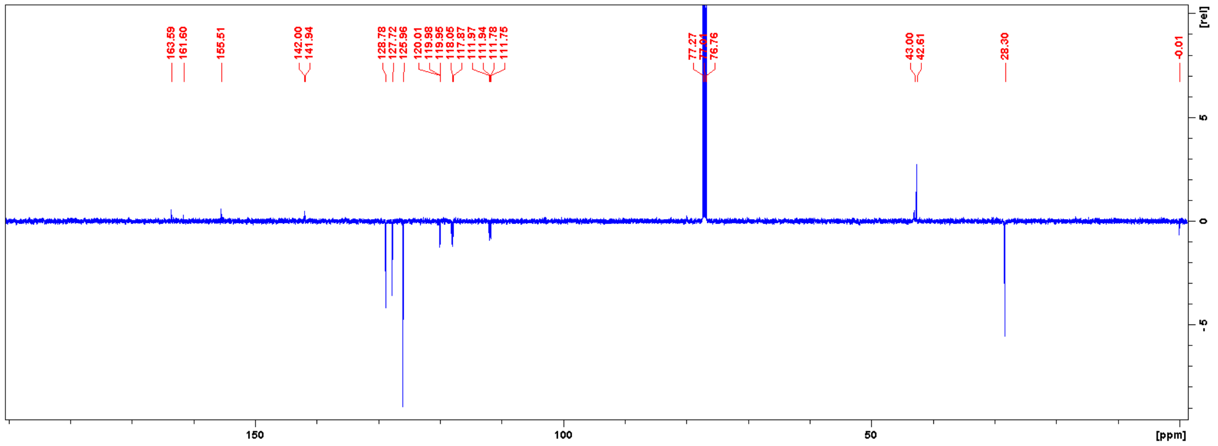

Figure S11. ^{1}H and ^{13}C NMR spectra of (±)-**14**

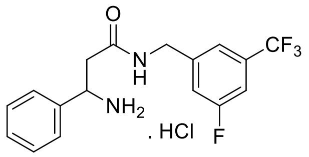

(±)-**14**

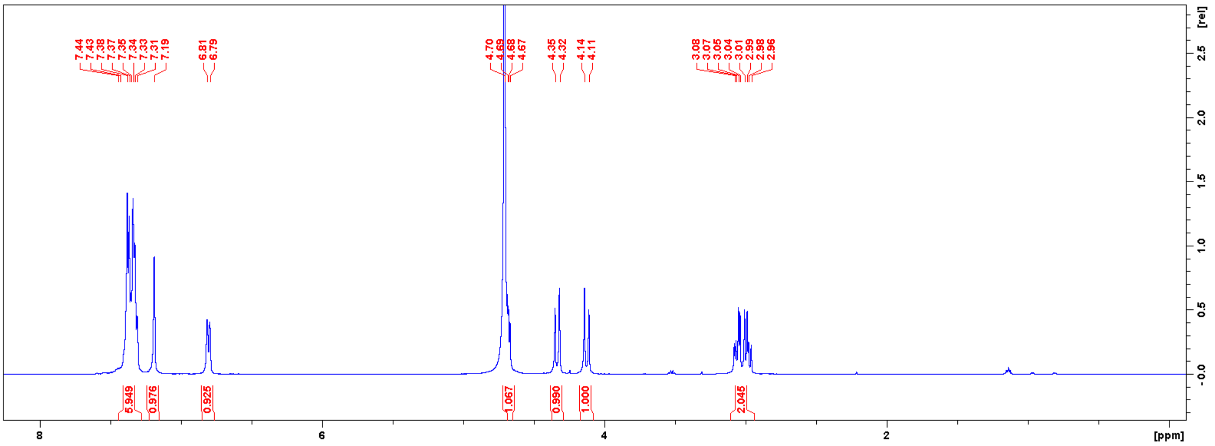

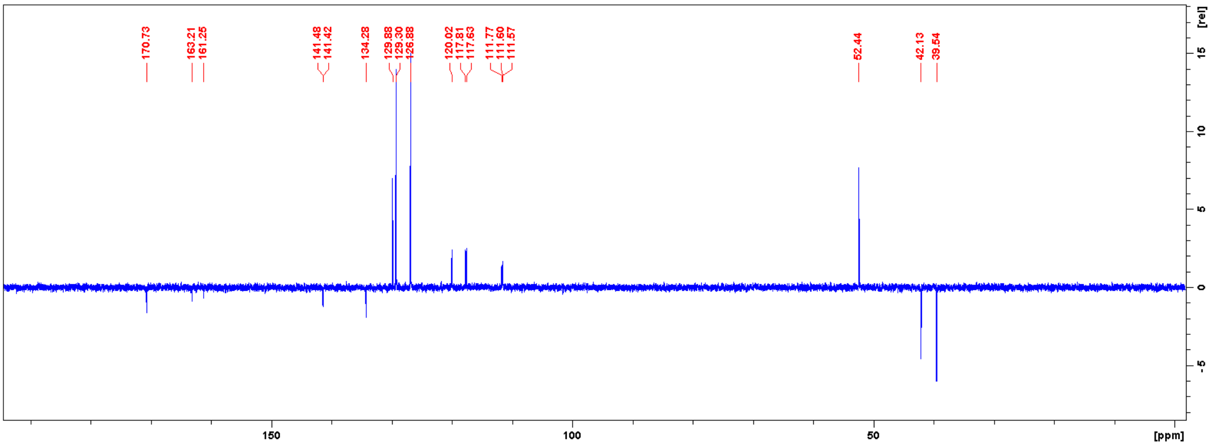

Figure S12. ^{1}H and ^{13}C NMR spectra of (±)-**16**

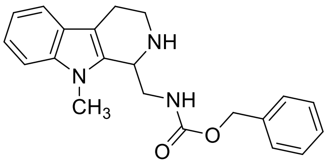

(±)-**16**

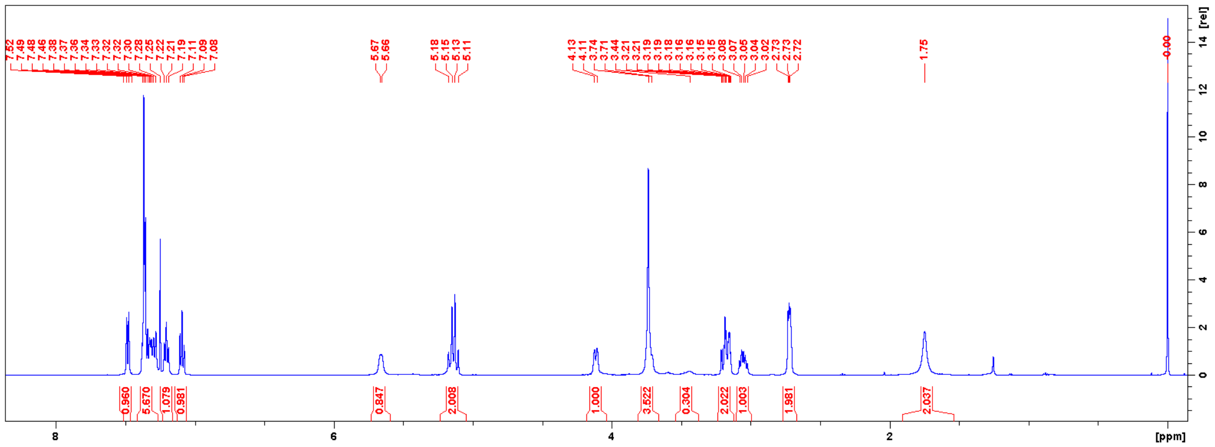

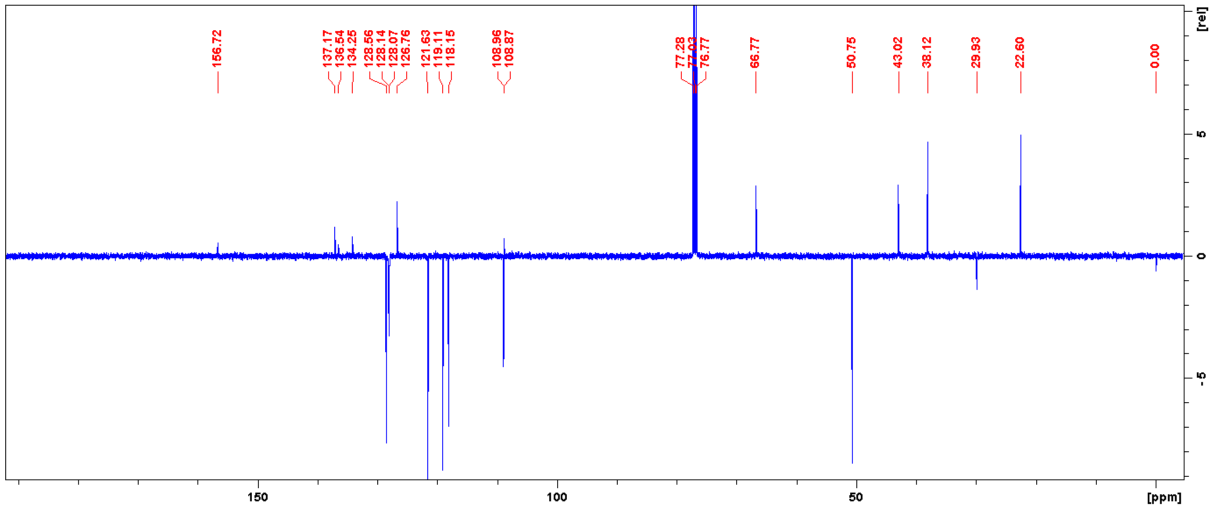

Figure S13. ^{1}H and ^{13}C NMR spectra of (±)-**19**

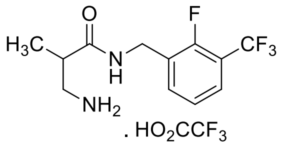

(±)-**19**

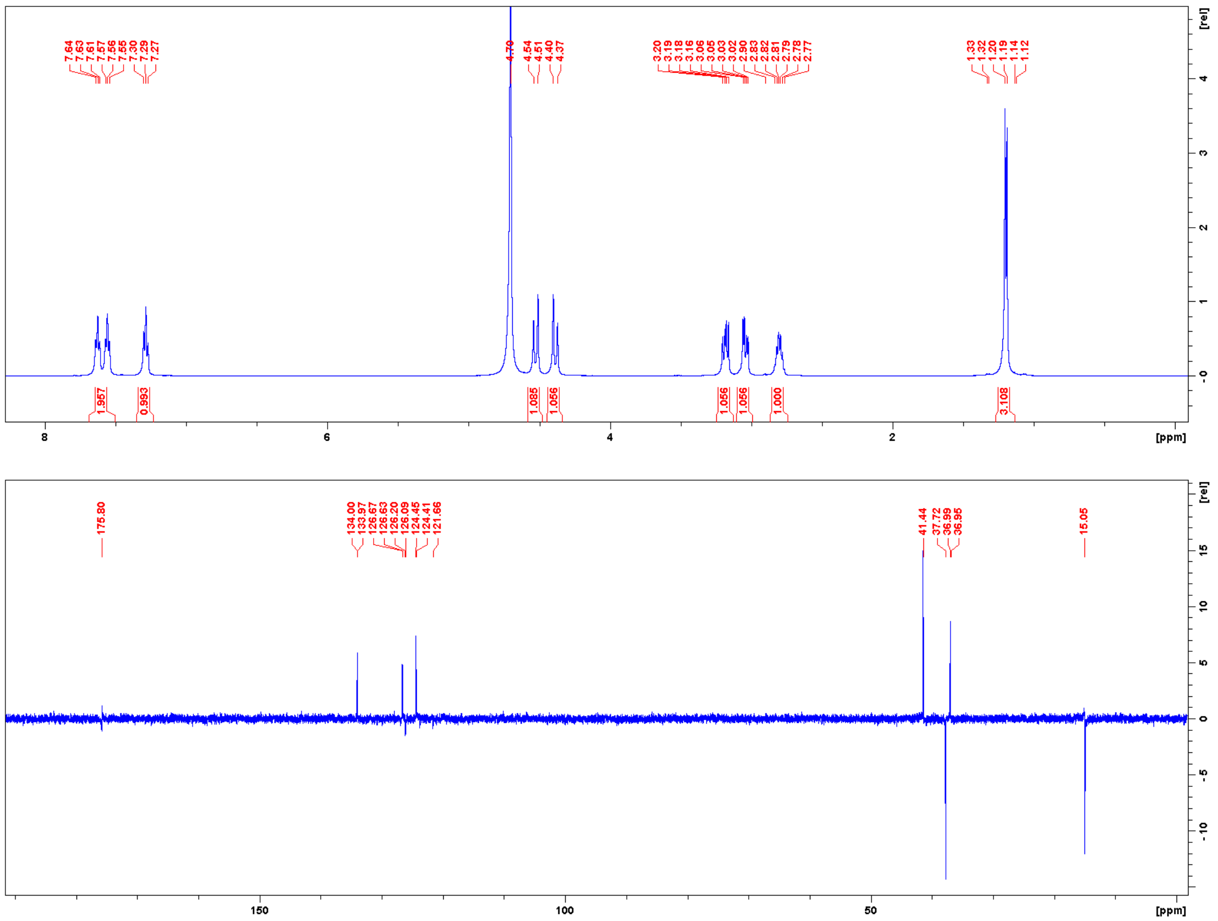

Figure S14. ^{1}H and ^{13}C NMR spectra of (±)-**21**

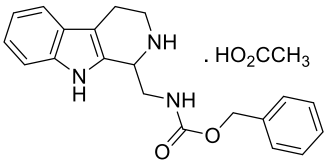

(±)-**21**

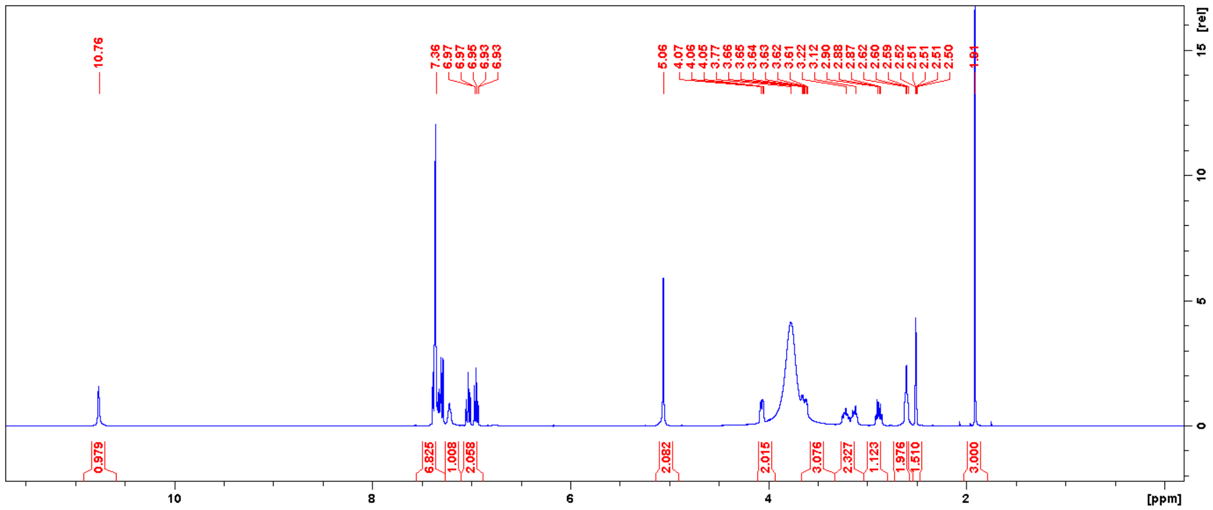

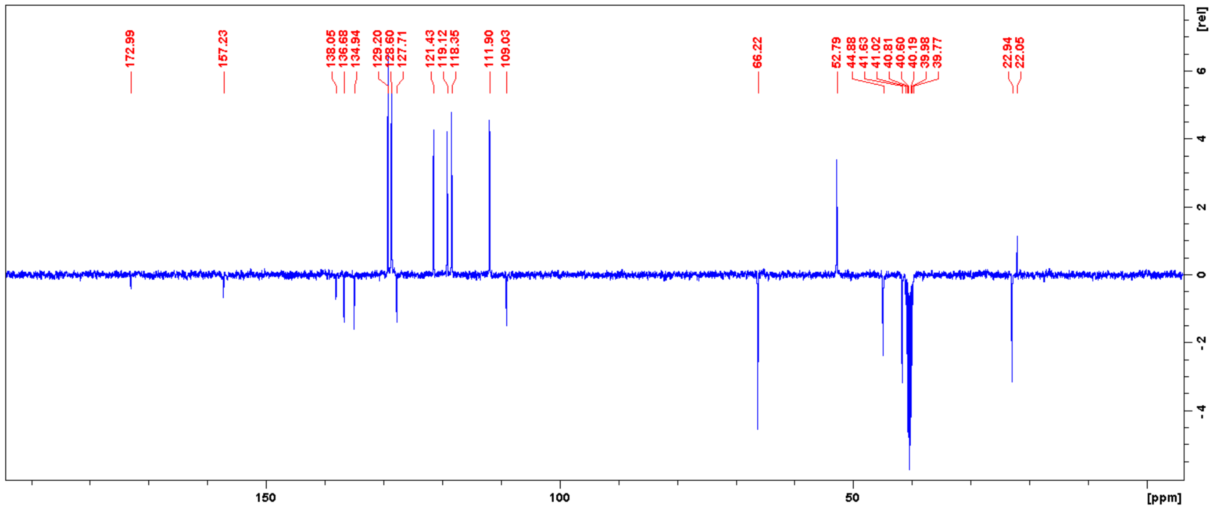

Figure S15. ^{1}H and ^{13}C NMR spectra of (±)-**23**

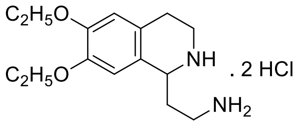

(±)-**23**

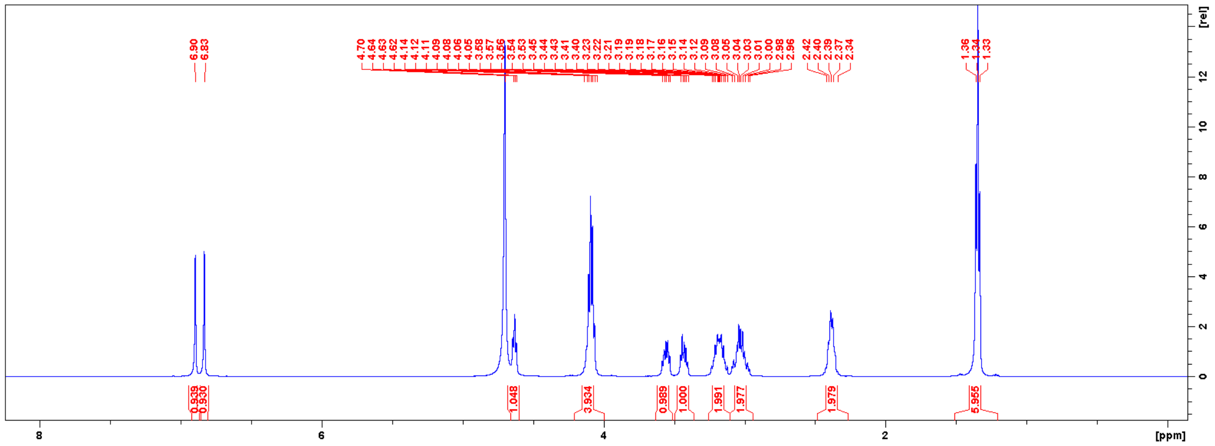

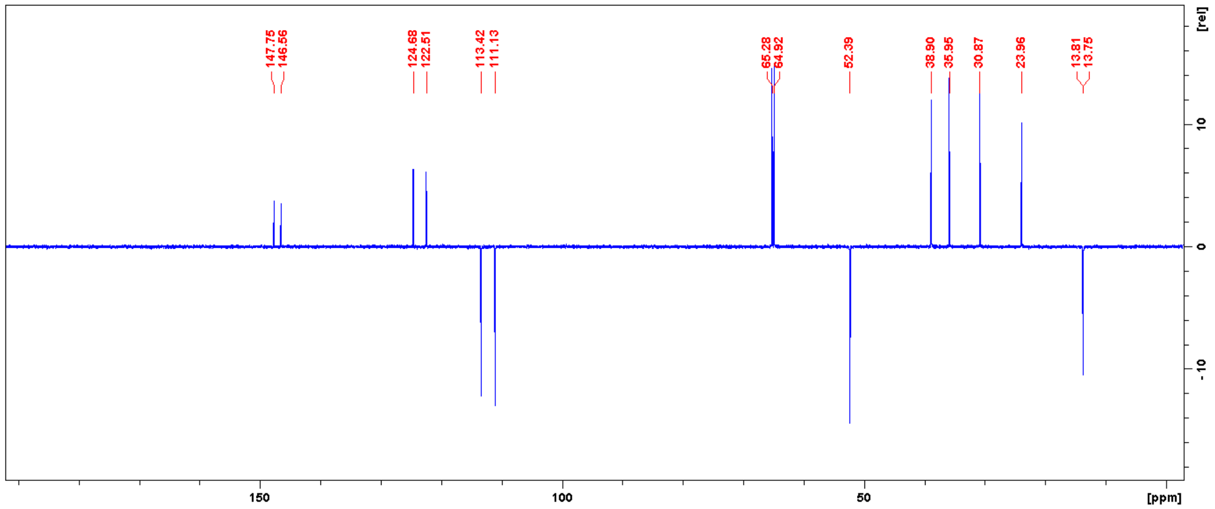

Figure S16. ^{1}H NMR, ^{13}C NMR, and NOESY spectra of (±)-**24**

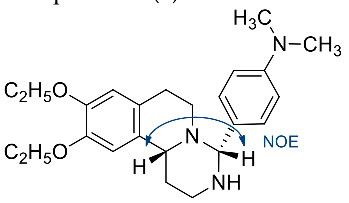

(±)-**24**

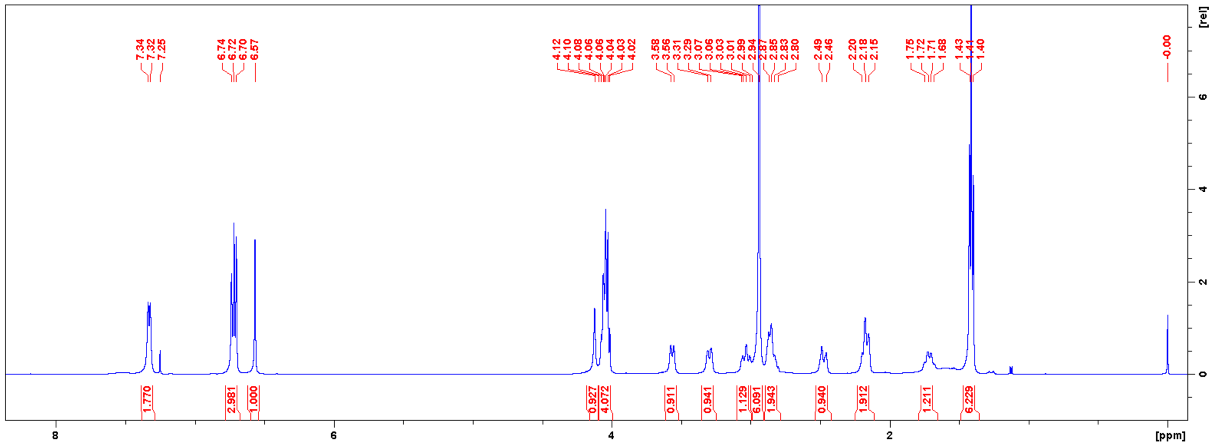

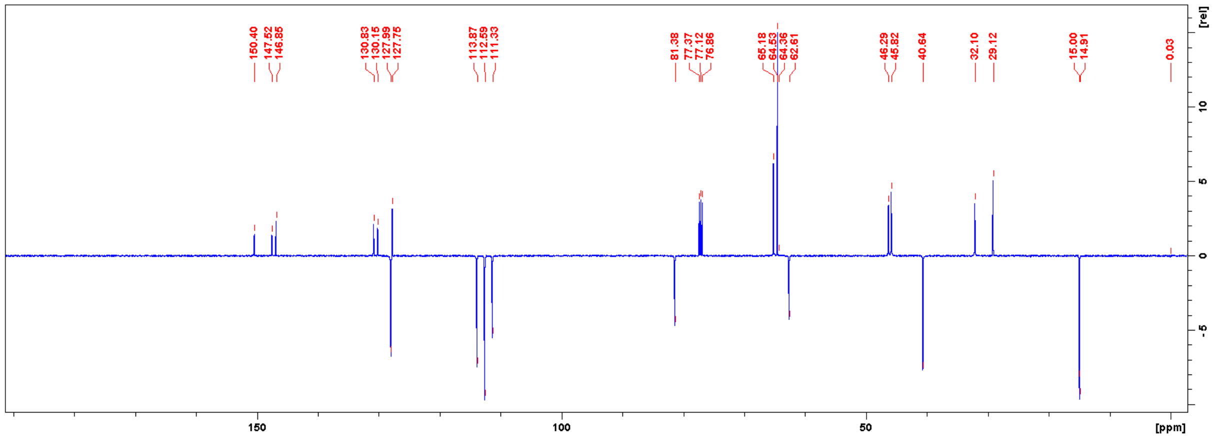

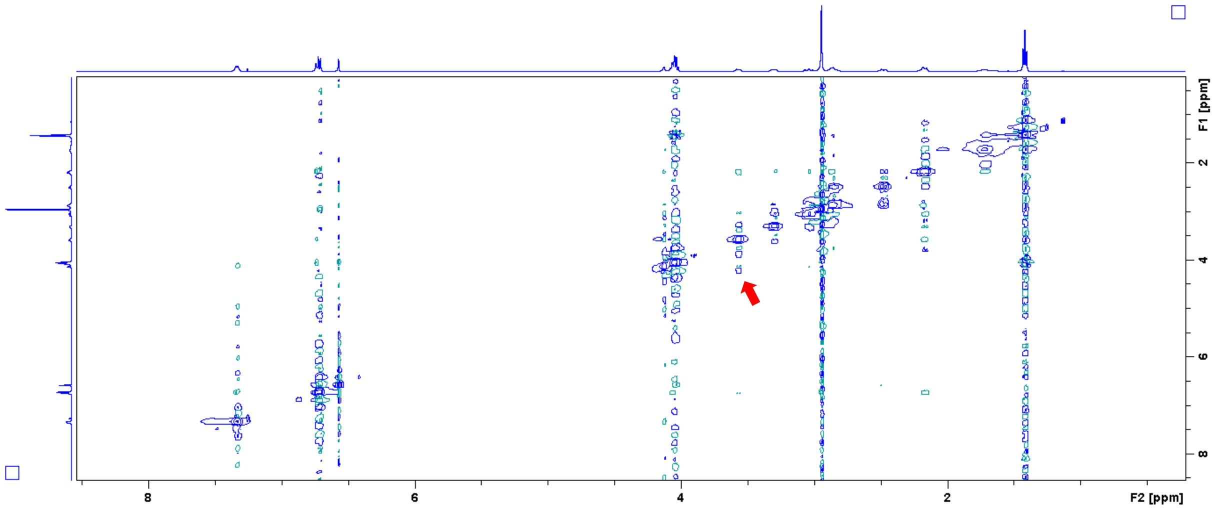

Figure S17. ^{1}H and ^{13}C NMR spectra of **26**

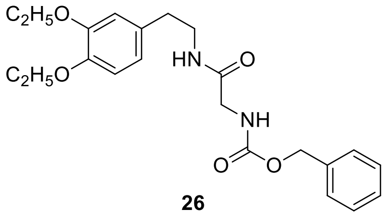

26

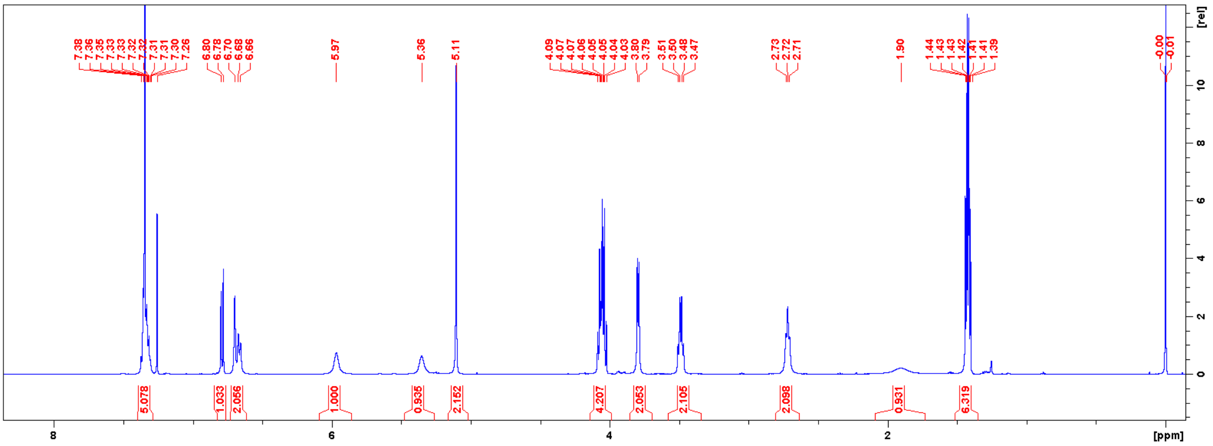

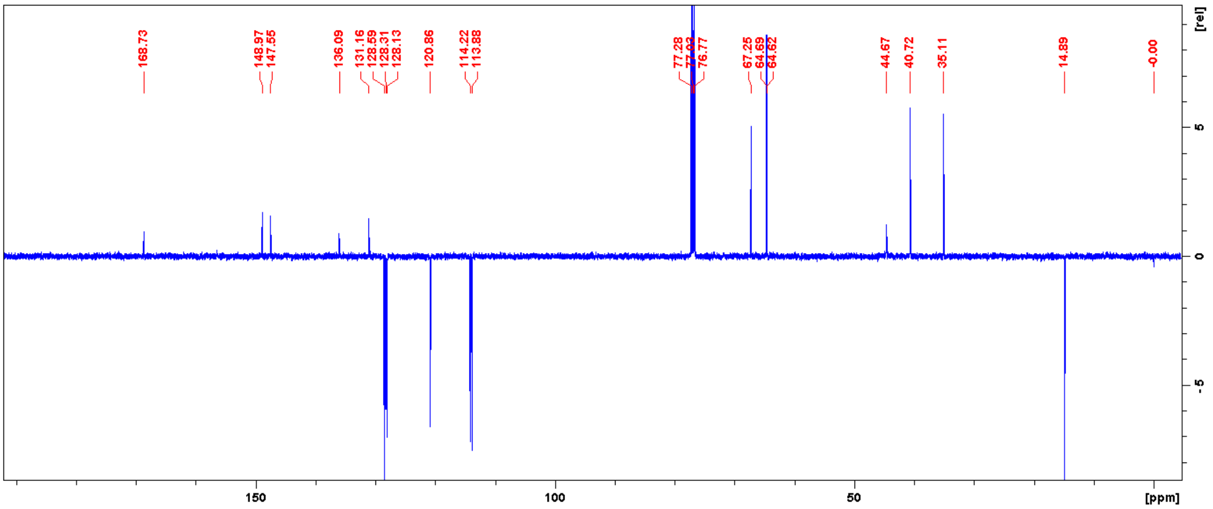

Figure S18. ^{1}H and ^{13}C NMR spectra of **27**

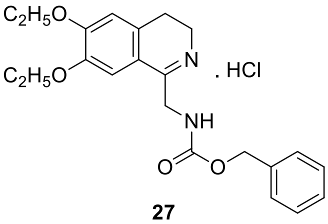

27

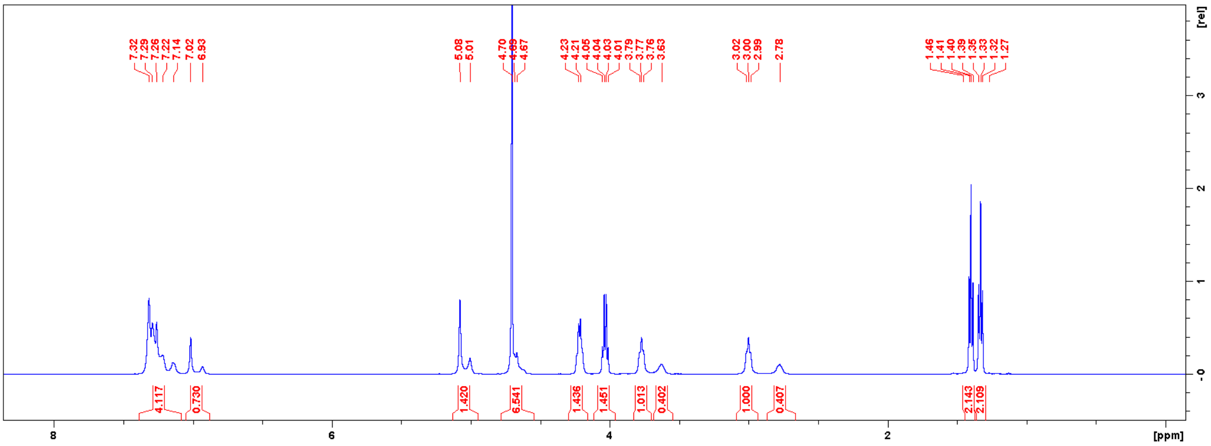

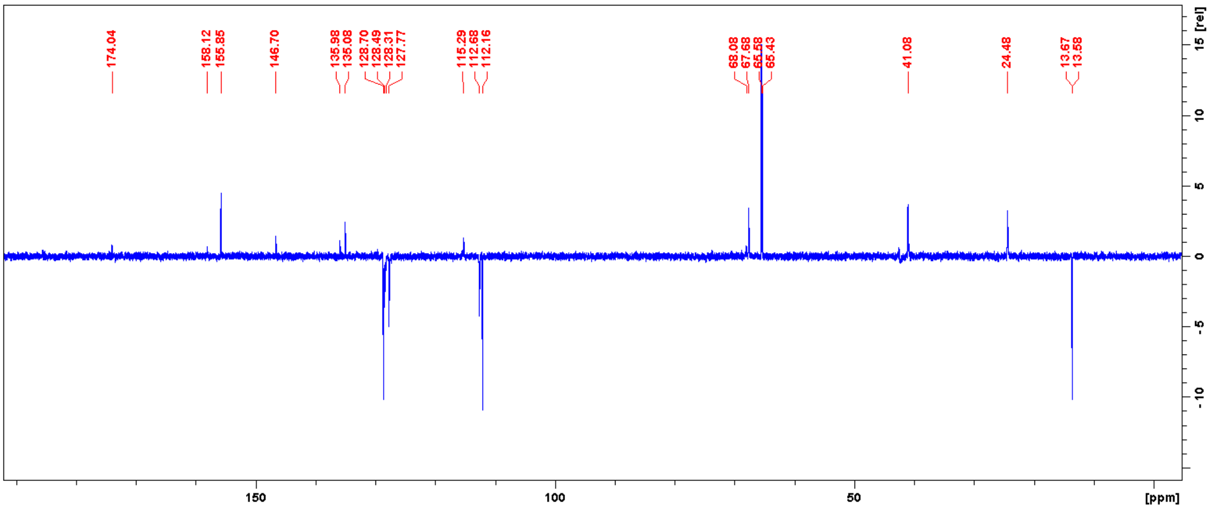

Figure S19. ^{1}H and ^{13}C NMR spectra of (±)-**28**

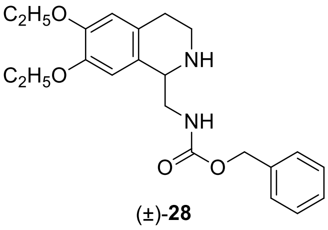

(±)-**28**

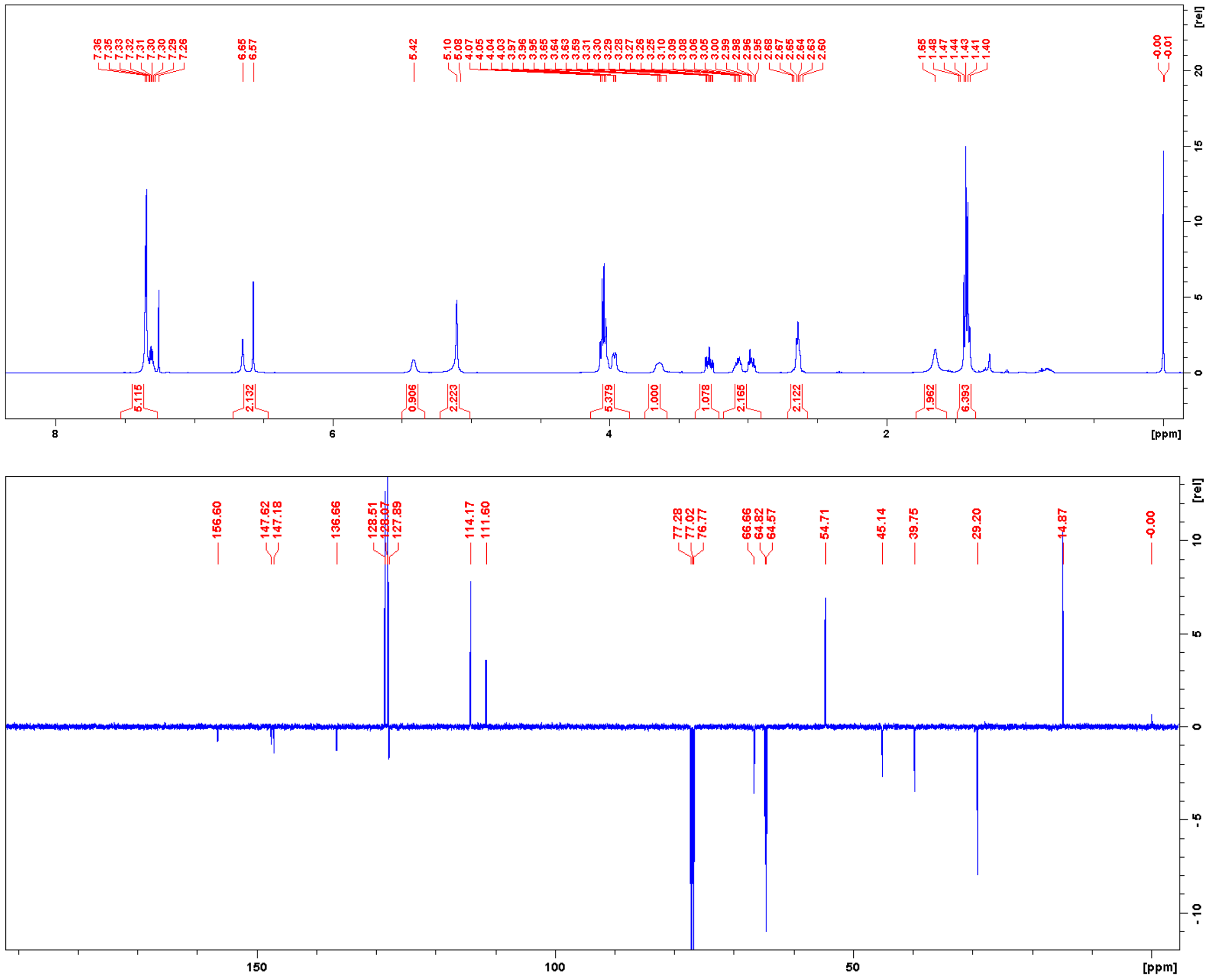

Figure S20. ^{1}H and ^{13}C NMR spectra of (±)-**30**

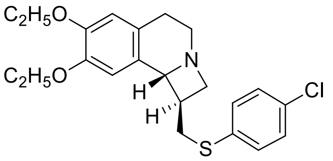

(±)-**30**

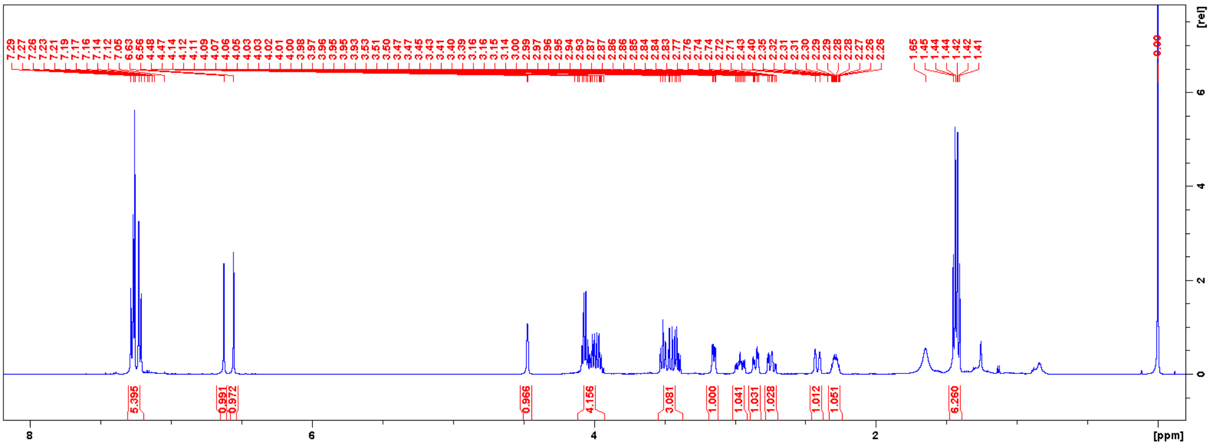

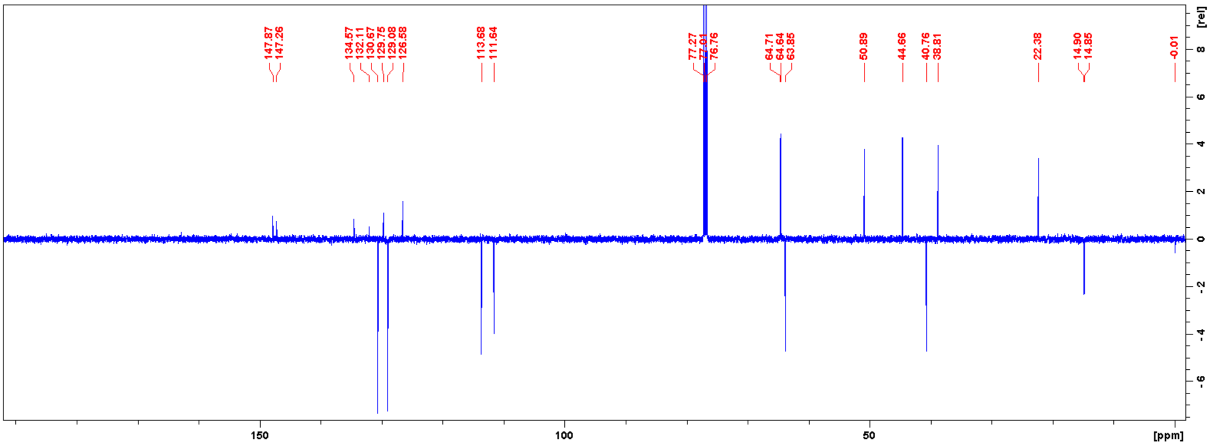

Figure S21. Chiral HPLC chromatograms of compounds (*R*)-**3**, **(*S*)-3**

The *ee* values of (*R*)-**3** and (*S*)-**3** were determined by HPLC using a ChiralPak ODH column. The analytical conditions were as follows: eluent: a mixture of *n*-hexane and isopropyl alcohol (IPA) (90:10) containing 0.1% Et_2N, flow rate: 0.5 mL min–1, detection at 240 nm, retention times: (*S*)-**3**: ca. 45 min, (*R*)-**3**: ca. 60 min.

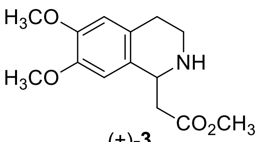

(±)-**3**

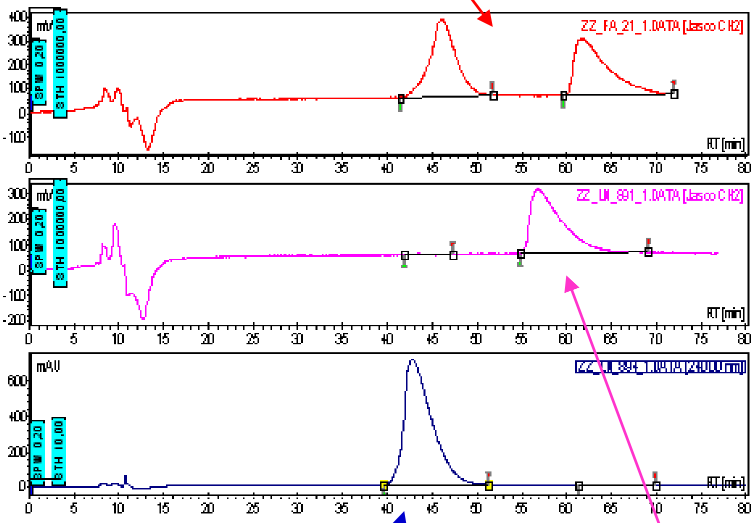

ZZ_FA_21_1.DATA [Jasco CH2]

Index	Name	Area	Area %	Time
		[mV.Min]	[%]	[Min]
1	UNKNOWN	1085,6	51,856	46,035
2	UNKNOWN	1007,9	48,144	61,708
Total		2093,5	100,000	

ZZ_LN_891_1.DATA [Jasco CH2]

Index	Name	Area	Area %	Time
		[mV.Min]	[%]	[Min]
1	UNKNOWN	1,8	0,173	43,600
2	UNKNOWN	1051,5	99,827	56,878
Total		1053,3	100,000	

ZZ_LN_894_1.DATA [240,00 nm]

Index	Name	Area	Area %	Time
		[mAU.Min]	[%]	[Min]
1	UNKNOWN	2580,7	99,619	42,718
2	UNKNOWN	9,9	0,381	65,363
Total		2590,5	100,000	

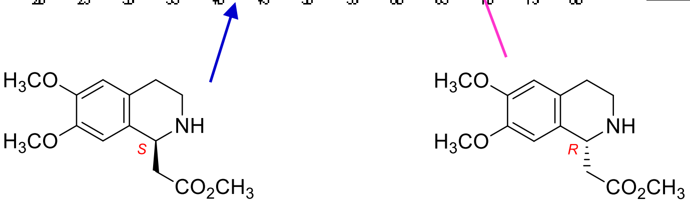

(*S*)-**3** (*R*)-**3**

Figure S22. Chiral HPLC chromatograms of compounds (*R*)-**9**, ***(S)*-9**

The *ee* values of (*R*)-**9** and (*S*)-**9** were determined by HPLC using Phenomenex-IA column. The analytical conditions were as follows: eluent: a mixture of *n*-hexane and isopropyl alcohol (IPA) (95:5), flow rate: 0.5 mL min–1, detection at 210 nm, retention times: (*R*)-**9**: 24.49 min, (*S*)-**9**: 25.73 min).

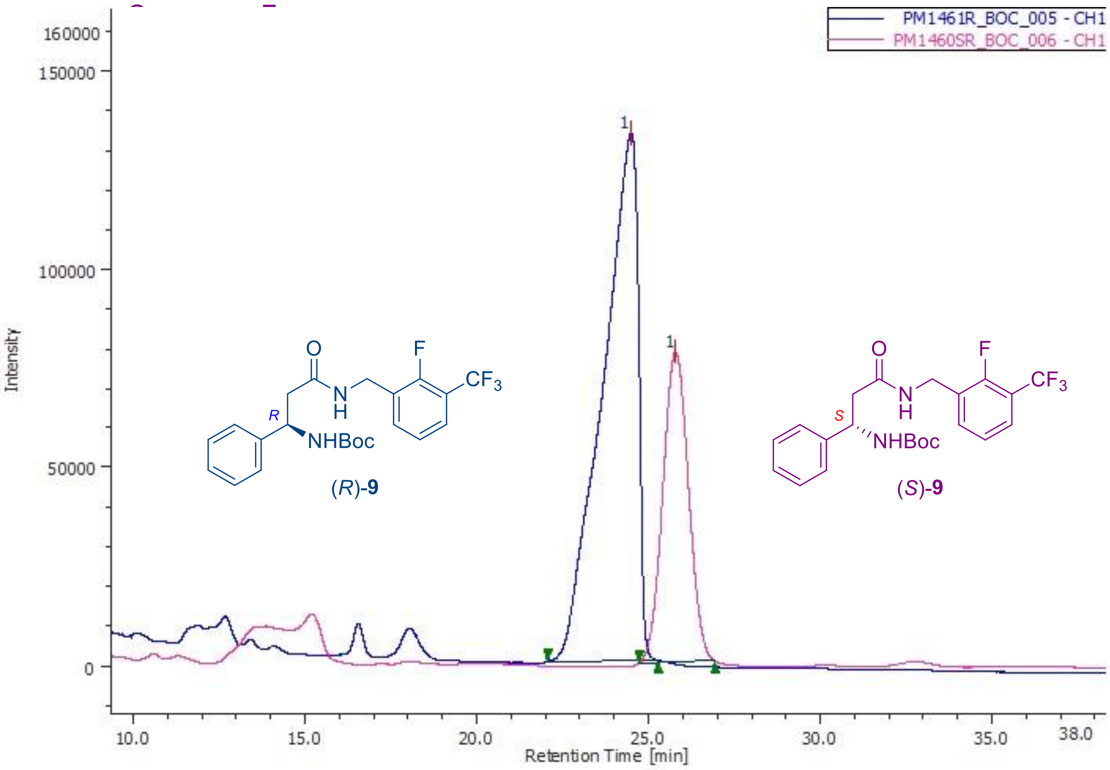

Supplement: Supplementary file 1 [file ijms-22-08112-s001.zip › SM-Synthesis.pdf]
