# Supplementary material for: Novel High Affinity Sigma-1 Receptor Ligands from Minimal Ensemble Docking-Based Virtual Screening
Source: Int J Mol Sci. 2021 Jul 29;22(15):8112. doi: 10.3390/ijms22158112 (PMC8347176; doi:10.3390/ijms22158112)
Supplement: Supplementary file 1 [file ijms-22-08112-s001.zip › SM_Figs.pdf]

# Supplementary information to:

# Novel high affinity sigma-1 receptor ligands from a minimal ensemble docking-based virtual screening

**Sz. Dvorácskó[1], L. Lázár[2], F. Fülöp[2†], M. Palkó[2], Z. Zalán[2], B. Penke[3], L. Fülöp[3*], Cs. Tömböly[1]. F. Bogár[3,4*]**

[1] Institute of Biochemistry, Biological Research Centre, Eötvös Loránd Research Network (ELKH), H-6726 Szeged, Temesvári krt. 62, Hungary
[2] Institute of Pharmaceutical Chemistry, University of Szeged, H-6720 Szeged, Eötvös u. 6, Hungary
[3] Department of Medical Chemistry, University of Szeged, H-6720 Szeged, Dóm Square 8, Hungary
[4] MTA-SZTE Biomimetic Systems Research Group, Eötvös Loránd Research Network (ELKH), H-6720 Szeged, Dóm Square 8, Hungary
* Correspondence: FB: bogar@sol.cc.u-szeged.hu; LF: fulop.livia@med.u-szeged.hu

# FIGURES

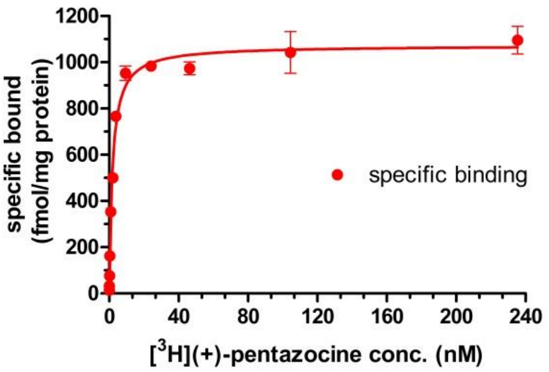


(**a**)

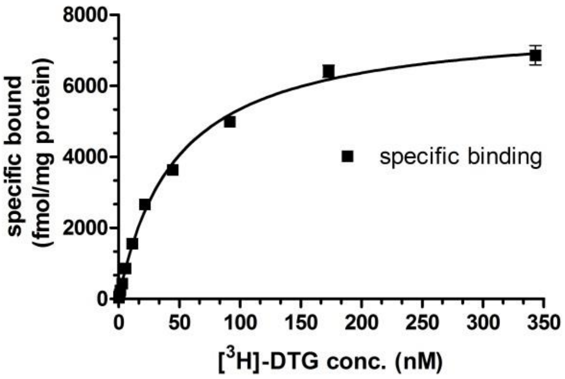


(**b**)

| | [$^3$H]-(+)-pentazocine | [$^3$H]-DTG |
|---|---|---|
| $K_d$ ± S.E.M. (nM) | 1.8 ± 0.17 | 47 ± 2.1 |
| $B_{max}$ ± S.E.M. (fmol/mg) | 1072 ± 20 | 7862 ± 140 |

(**c**)

**Figure S1.** Saturation isotherms of specific binding of (**a**) [$^3$H]-(+)-pentazocine on guinea pig liver membrane homogenate, and (**b**) [$^3$H]-DTG on rat liver membranes in the presence of 100 nM (+)-pentazocine. Nonspecific binding was determined in the presence of 10 μM haloperidol. Specific binding data are presented as means ± S.E.M of at least 3 independent experiments. (**c**) equilibrium binding data of [$^3$H]-(+)-pentazocine and [$^3$H]-DTG, i.e. dissociation equilibrium constant ($K_d$) and receptor density ($B_{max}$) values were calculated by fitting the saturation isotherms.

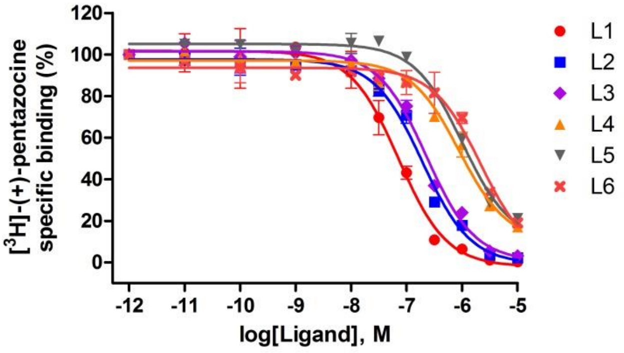


(**a**)

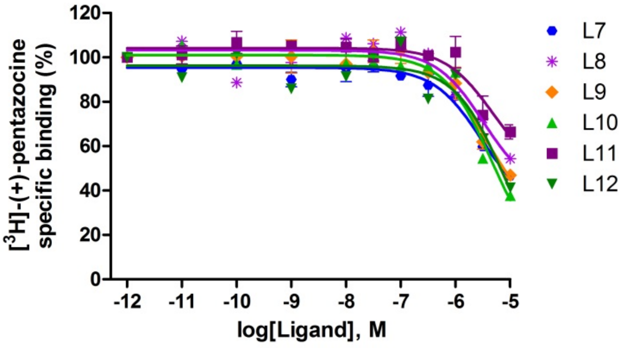


(**b**)

**Figure S2.** Competition of racemic ligands (**a**) L1-L6 and (**b**) L7-L12 with [$^3$H]-(+)-pentazocine binding to S1R sites in guinea pig liver membranes.

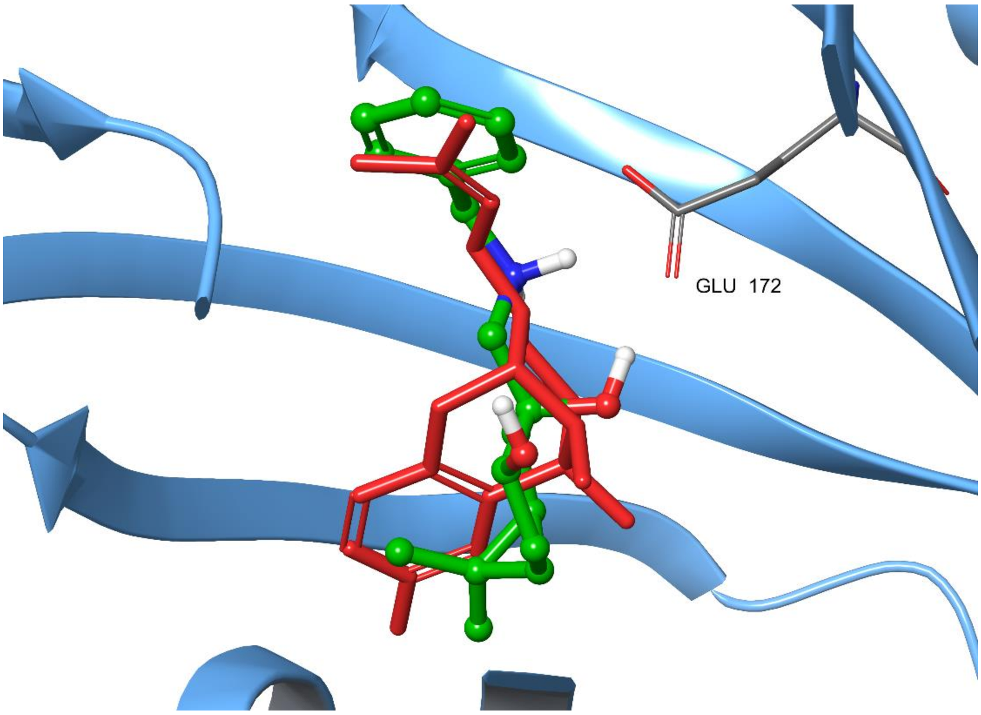


**Figure S3.** Binding pose of L7 (green carbons) and (+)-pentazocine (red) in the binding pocket of S1R (6DK1C, blue cartoon)

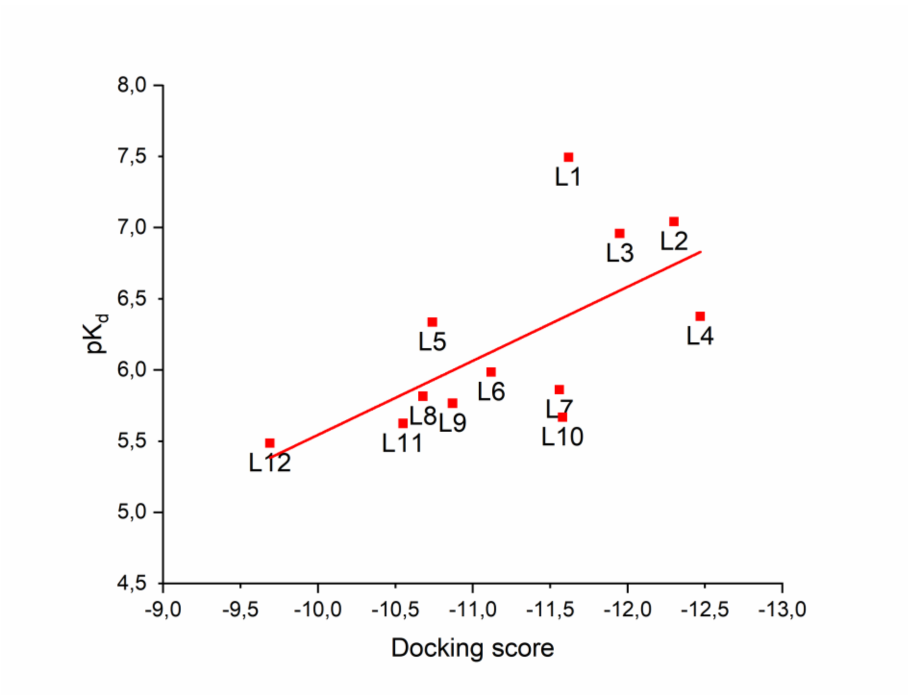


**Figure S4.** The correlation of experimental $pK_d$ values and the docking scores of the active ligands obtained from minimal ensemble virtual screening.

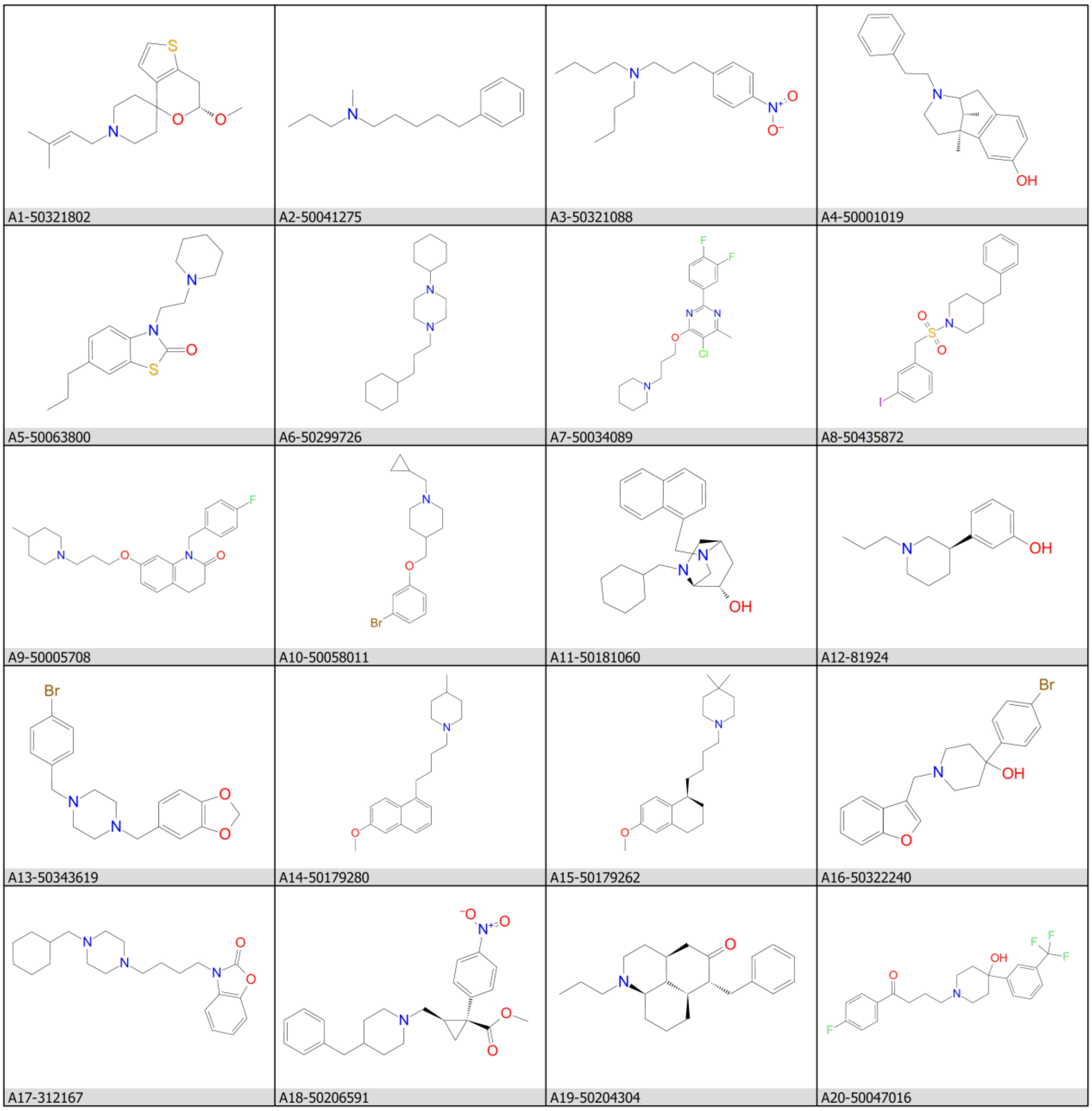


**Figure S5.** Diverse subset of twenty S1R ligands selected from the 190 compounds with $K_i \leq 1$nM collected from the BindingDB. After the ligand name (A-A20) the BindingDB ID is also shown.

| | A1 | A2 | A3 | A4 | A5 | A6 | A7 | A8 | A9 | A10 | A11 | A12 | A13 | A14 | A15 | A16 | A17 | A18 | A19 | A20 |
|---|---|---|---|---|---|---|---|---|---|---|---|---|---|---|---|---|---|---|---|---|
| A1 | 1,00 | 0,10 | 0,10 | 0,14 | 0,09 | 0,06 | 0,04 | 0,06 | 0,07 | 0,07 | 0,08 | 0,04 | 0,07 | 0,06 | 0,10 | 0,06 | 0,04 | 0,10 | 0,07 | 0,08 |
| A2 | 0,10 | 1,00 | 0,14 | 0,15 | 0,14 | 0,05 | 0,05 | 0,06 | 0,13 | 0,09 | 0,09 | 0,07 | 0,09 | 0,09 | 0,09 | 0,11 | 0,05 | 0,13 | 0,12 | 0,08 |
| A3 | 0,10 | 0,14 | 1,00 | 0,11 | 0,11 | 0,06 | 0,03 | 0,07 | 0,08 | 0,09 | 0,08 | 0,04 | 0,08 | 0,06 | 0,08 | 0,10 | 0,05 | 0,09 | 0,08 | 0,08 |
| A4 | 0,14 | 0,15 | 0,11 | 1,00 | 0,13 | 0,08 | 0,05 | 0,07 | 0,08 | 0,09 | 0,13 | 0,06 | 0,13 | 0,06 | 0,11 | 0,08 | 0,05 | 0,10 | 0,11 | 0,08 |
| A5 | 0,09 | 0,14 | 0,11 | 0,13 | 1,00 | 0,07 | 0,03 | 0,06 | 0,13 | 0,09 | 0,11 | 0,06 | 0,13 | 0,07 | 0,11 | 0,07 | 0,04 | 0,13 | 0,13 | 0,08 |
| A6 | 0,06 | 0,05 | 0,06 | 0,08 | 0,07 | 1,00 | 0,07 | 0,13 | 0,06 | 0,07 | 0,08 | 0,06 | 0,09 | 0,07 | 0,10 | 0,06 | 0,14 | 0,06 | 0,09 | 0,06 |
| A7 | 0,04 | 0,05 | 0,03 | 0,05 | 0,03 | 0,07 | 1,00 | 0,07 | 0,06 | 0,04 | 0,06 | 0,11 | 0,08 | 0,05 | 0,09 | 0,03 | 0,16 | 0,04 | 0,05 | 0,03 |
| A8 | 0,06 | 0,06 | 0,07 | 0,07 | 0,06 | 0,13 | 0,07 | 1,00 | 0,05 | 0,10 | 0,07 | 0,05 | 0,08 | 0,08 | 0,11 | 0,05 | 0,08 | 0,05 | 0,06 | 0,07 |
| A9 | 0,07 | 0,13 | 0,08 | 0,08 | 0,13 | 0,06 | 0,06 | 0,05 | 1,00 | 0,07 | 0,09 | 0,07 | 0,11 | 0,10 | 0,07 | 0,08 | 0,07 | 0,15 | 0,16 | 0,08 |
| A10 | 0,07 | 0,09 | 0,09 | 0,09 | 0,09 | 0,07 | 0,04 | 0,10 | 0,07 | 1,00 | 0,09 | 0,05 | 0,10 | 0,10 | 0,11 | 0,05 | 0,05 | 0,08 | 0,09 | 0,12 |
| A11 | 0,08 | 0,09 | 0,08 | 0,13 | 0,11 | 0,08 | 0,06 | 0,07 | 0,09 | 0,09 | 1,00 | 0,07 | 0,11 | 0,06 | 0,11 | 0,06 | 0,06 | 0,10 | 0,10 | 0,08 |
| A12 | 0,04 | 0,07 | 0,04 | 0,06 | 0,06 | 0,06 | 0,11 | 0,05 | 0,07 | 0,05 | 0,07 | 1,00 | 0,09 | 0,05 | 0,06 | 0,11 | 0,14 | 0,06 | 0,07 | 0,05 |
| A13 | 0,07 | 0,09 | 0,08 | 0,13 | 0,13 | 0,09 | 0,08 | 0,08 | 0,11 | 0,10 | 0,11 | 0,09 | 1,00 | 0,07 | 0,12 | 0,08 | 0,08 | 0,10 | 0,12 | 0,10 |
| A14 | 0,06 | 0,09 | 0,06 | 0,06 | 0,07 | 0,07 | 0,05 | 0,08 | 0,10 | 0,10 | 0,06 | 0,05 | 0,07 | 1,00 | 0,08 | 0,12 | 0,05 | 0,06 | 0,06 | 0,06 |
| A15 | 0,10 | 0,09 | 0,08 | 0,11 | 0,11 | 0,10 | 0,09 | 0,11 | 0,07 | 0,11 | 0,11 | 0,06 | 0,12 | 0,08 | 1,00 | 0,06 | 0,10 | 0,08 | 0,07 | 0,07 |
| A16 | 0,06 | 0,11 | 0,10 | 0,08 | 0,07 | 0,06 | 0,03 | 0,05 | 0,08 | 0,05 | 0,06 | 0,11 | 0,08 | 0,12 | 0,06 | 1,00 | 0,09 | 0,08 | 0,05 | 0,09 |
| A17 | 0,04 | 0,05 | 0,05 | 0,05 | 0,04 | 0,14 | 0,16 | 0,08 | 0,07 | 0,05 | 0,06 | 0,14 | 0,08 | 0,05 | 0,10 | 0,09 | 1,00 | 0,06 | 0,06 | 0,05 |
| A18 | 0,10 | 0,13 | 0,09 | 0,10 | 0,13 | 0,06 | 0,04 | 0,05 | 0,15 | 0,08 | 0,10 | 0,06 | 0,10 | 0,06 | 0,08 | 0,08 | 0,06 | 1,00 | 0,16 | 0,10 |
| A19 | 0,07 | 0,12 | 0,08 | 0,11 | 0,13 | 0,09 | 0,05 | 0,06 | 0,16 | 0,09 | 0,10 | 0,07 | 0,12 | 0,06 | 0,07 | 0,05 | 0,06 | 0,16 | 1,00 | 0,08 |
| A20 | 0,08 | 0,08 | 0,08 | 0,08 | 0,08 | 0,06 | 0,03 | 0,07 | 0,08 | 0,12 | 0,08 | 0,05 | 0,10 | 0,06 | 0,07 | 0,09 | 0,05 | 0,10 | 0,08 | 1,00 |

**Figure S6.** Similarity matrix of the active compounds (A1-A20) used in the enrichment studies. The highest cross-similarity is 0.16. Light yellow is used for similarity smaller than 0.1, and dark yellow for the values between 0.1 and 0.16.
